# Evolutionary triangulation: informing genetic association studies with evolutionary evidence

**DOI:** 10.1186/s13040-016-0091-7

**Published:** 2016-04-02

**Authors:** Minjun Huang, Britney E. Graham, Ge Zhang, Reed Harder, Nuri Kodaman, Jason H. Moore, Louis Muglia, Scott M. Williams

**Affiliations:** Department of Genetics, Dartmouth College, Geisel School of Medicine, Hanover, NH USA; Institute for Quantitative Biomedical Sciences, Dartmouth College, Hanover, NH USA; Human Genetics Division, Cincinnati Children’s Hospital Medical Center, Cincinnati, OH USA; Perelman School of Medicine, University of Pennsylvania, Philadelphia, PA USA; Center for Prevention of Preterm Birth, Perinatal Institute, Cincinnati Children’s Hospital Medical Center, and March of Dimes Prematurity Research Center Ohio Collaborative, Cincinnati, OH USA; Present Address: Department of Epidemiology and Biostatistics, Case Western Reserve University, 10900 Euclid Avenue, Cleveland, OH 44106 USA

**Keywords:** Genetic association, Population differentiation, Health disparity, Selection

## Abstract

Genetic studies of human diseases have identified many variants associated with pathogenesis and severity. However, most studies have used only statistical association to assess putative relationships to disease, and ignored other factors for evaluation. For example, evolution is a factor that has shaped disease risk, changing allele frequencies as human populations migrated into and inhabited new environments. Since many common variants differ among populations in frequency, as does disease prevalence, we hypothesized that patterns of disease and population structure, taken together, will inform association studies. Thus, the population distributions of allelic risk variants should reflect the distributions of their associated diseases. Evolutionary Triangulation (ET) exploits this evolutionary differentiation by comparing population structure among three populations with variable patterns of disease prevalence. By selecting populations based on patterns where two have similar rates of disease that differ substantially from a third, we performed a proof of principle analysis for this method. We examined three disease phenotypes, lactase persistence, melanoma, and Type 2 diabetes mellitus. We show that for lactase persistence, a phenotype with a simple genetic architecture, ET identifies the key gene, lactase. For melanoma, ET identifies several genes associated with this disease and/or phenotypes related to it, such as skin color genes. ET was less obviously successful for Type 2 diabetes mellitus, perhaps because of the small effect sizes in known risk loci and recent environmental changes that have altered disease risk. Alternatively, ET may have revealed new genes involved in conferring disease risk for diabetes that did not meet nominal GWAS significance thresholds. We also compared ET to another method used to filter for phenotype associated genes, population branch statistic (PBS), and show that ET performs better in identifying genes known to associate with diseases appropriately distributed among populations. Our results indicate that ET can filter association results to improve our ability to discover disease loci.

**Electronic supplementary material:**

The online version of this article (doi:10.1186/s13040-016-0091-7) contains supplementary material, which is available to authorized users.

## Introduction

As humans moved out of Africa, population patterns of genetic variation changed dramatically, due to both random (e.g., genetic drift that can cause serial founder events) and non-random (environment-specific selection) processes. The histories of the different populations have therefore resulted in substantial differences among populations in allele frequencies at loci throughout the genome [1]. Many, if not most of these differences, will have limited effect on phenotypic variation, but some allelic substitutions may have implications for differences in phenotypic variation among populations that may help inform us in the search for genes of biomedical significance. Specifically, it could be hypothesized that the alleles that affect disease risk in multiple populations should be distributed in ways that are consistent with differences in population prevalence, such that risk alleles should be more frequent in those populations where a given disease is more prevalent and less frequent where the disease is relatively rare.

**Fig. 1:**
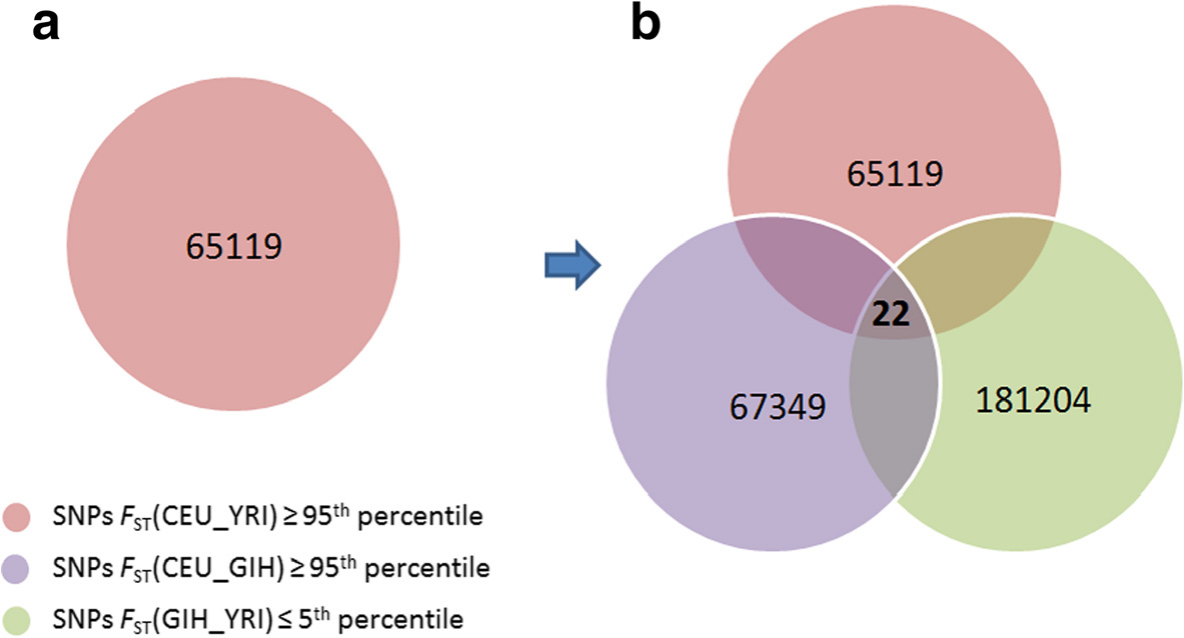
Limiting the number of SNPs by switching from pairwise to three-way comparison. The number of SNPs identified in pairwise versus three-way population comparisons are illustrated by showing each two-analysis and the three-way overlap. The number in each circle indicates the number of SNPs that are identified under a particular *F*_ST_ threshold for a pairwise comparison; the numbers are shown in the lower left legend. For example, **a** indicates there are 65119 SNPs under 95th percentile *F*_ST_ threshold in the comparison between CEU and YRI. **b** shows that there is a significant decrease to 22 SNPs (center triangle) when using a three way comparison under the thresholds of the same stringency

Screening variants for differences in allele frequencies among populations may serve as an effective filter to identify candidates for disease, even in the absence of prior physiological evidence for disease-related function. However, because of the large number of allele frequency differences among any two populations [2], pairwise comparisons between most human populations will generate too large a number of differentiated single nucleotide polymorphisms (SNPs) to be of much practical use; any single comparison is likely to generate too many possible loci, most of which are unlikely to be related to a specific phenotype or class of phenotypes (Fig. 1a). We hypothesize that adding a third population with similar disease prevalence to one of the other two populations being compared increases our ability to define genomic regions of particular interest with respect to disease or phenotypic variation by removing the vast majority of loci or SNPs that, although highly differentiated, are unlikely to associate with phenotypes of interest (Fig. 1b). The intersection of variants that have similar allele frequencies in populations with similar disease prevalences, and different allele frequencies between populations with different prevalences, should yield an enrichment of genes that associate with a given disease. We define such variants as “appropriately distributed” with respect to a given phenotype. Similarly, diseases that we will call “appropriately distributed” are those that have prevalences distributed consistently with the allele frequency patterns of variation.

In this manuscript, we present a heuristic approach that incorporates genetic and epidemiological information – namely, known disease distributions with pairwise allele frequency differences among three populations – to filter association results from other genetic epidemiological analyses, such as genome wide association studies (GWAS). We call this approach Evolutionary Triangulation (ET), as it represents comparisons of three genetically distinct populations, assayed simultaneously. To assess levels of differentiation, we use Wright’s *F*_ST_ [3], which is a metric that provides an estimate of the level of allele frequency differences among populations. To assess the performance and to determine the limits of our approach, we applied ET to a number of phenotypes that vary in presumed genetic architecture, ranging from an essentially Mendelian trait (lactase persistence), to diseases of increasing complexity, i.e., melanoma, representing an oligogenic disease, and fasting glucose/Type 2 diabetes mellitus as a highly complex disease with many genes of small effect.

## Methods

### Index phenotype and population selection

We selected three index phenotypes in this study: lactase persistence, melanoma, and Type 2 diabetes mellitus/fasting glucose, based on prior epidemiological data, most of which were derived from the World Health Organization’s website (http://www.who.int/en/) or current literature (Additional file 1: Table S1). Our selection of phenotypes was further guided by the availability of genetic data from appropriate HapMap populations that coincide with the epidemiological data (Additional file 1: Table S1). For instance, melanoma is rare in South Asians and Africans, but common in Europeans (e.g. ~150 fold greater prevalence than in South Asians), matching the HapMap populations GIH, YRI and CEU. Additionally, these phenotypes represent a range of underlying genetic architectures, allowing us to assess whether ET is applicable to a broad variety of traits, from effectively Mendelian diseases to complex disorders with small effect sizes (e.g., odds ratios as low as 1.04)(Table 1).

**Table 1:** Genetic associations of the 85th/15th threshold ET genes with index diseases/traits (CEU-GIH-YRI)

Index disease	ET genes	5th^a^	10th^a^	Odds ratio^b^
Lactase Persistence	*LCT*	Y	Y	Mendelian
Melanoma/Skin Neoplasms	*OCA2*			3.16 [58]
	*SLC45A2*	Y	Y	2.78 [15]
	*TYRP1*			1.15 [59]
	*XRCC1*			0.60 [60]
Diabetes Mellitus, Type 2/Glucose Intolerance/Insulin Resistance^c^	*ADAMTS9*			1.12 [61]
	*DGKB*			1.04 [62]
	*FTO*			1.17 [63]
	*IDE*			1.28 [64]
	*IGF2BP2*		Y	1.22 [65]
	*IL6*		Y	1.29 [66]
	*SH2B1*		Y	1.16 [67]
	*SREBF1*		Y	1.17 [68]

^a^5th/10th: Y indicates ET genes identified under the 95th/5th or 90th/10th threshold, respectively

^b^Largest reported Odds ratio

^c^Case diagnosis is as defined in each reference

### Triangulation for ET SNPs and mapping to ET genes

We chose representative HapMap populations based on the prevalences of the index phenotypes (Additional file 1: Table S1). We obtained all SNP allele frequency data for unrelated individuals from the selected populations in the International HapMap Project Phase III [4], which included 113 Utah residents with Northern and Western European ethnicity (CEU), 84 Han Chinese from Beijing, China (CHB), 88 Tuscans from Italy (TSI), 88 Gujarati Indians from Houston (GIH), 113 Yoruba from Ibadan, Nigeria (YRI), 86 Japanese in Tokyo, Japan (JPT), and 50 Mexican Americans in Los Angeles, California (MEX). CEU was a proxy for Northern European populations, CHB and JPT for East Asian populations, TSI for Southern European populations, GIH for South Asian populations, MEX for Central American Hispanic populations, and YRI for West African populations.

**Fig. 2:**
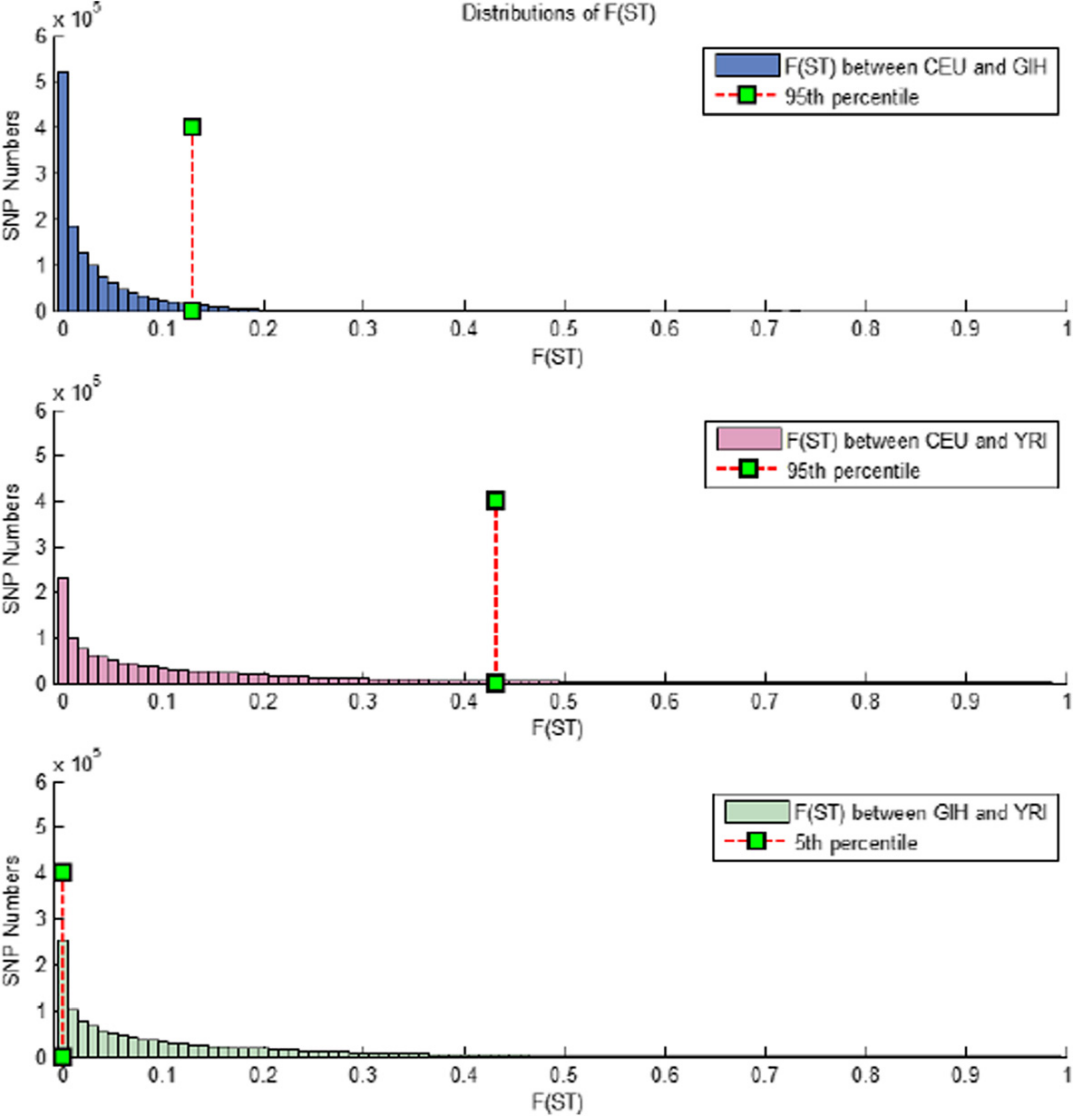
*F*_ST_ distributions among ET comparisons of CEU, GIH and YRI populations. Shows the *F*_ST_ distributions of CEU-GIH, CEU-YRI and GIH-YRI. Similar in the three distributions, most *F*_ST_’s are less than 0.1, which means that most SNPs are not differentiated greatly among population. The red dotted lines indicates the percentile thresholds we use to generate ET SNPs. For example, for CEU-GIH and CEU-YRI we took SNPs having an *F*_ST_ greater than or equal to 95th percentile, which are those to right of the red dotted lines. And for GIH-YRI, we took SNPs having an *F*_ST_ less than or equal to than 5th percentile, which are those to left of the red dotted line. By overlapping these three sets of SNPs, we could generate the ET SNPs

To assess the population genetic differences we calculated unbiased estimates of *F*_ST_ based on HapMap allele frequency data according to the Weir and Cockerham formula [5]. For example, among the CEU, GIH and YRI, as shown in Fig. 1, the *F*_ST_ of each SNP was calculated pairwise. ET SNPs (center triangle, Fig. 1b) were selected according to the overlaps of high *F*_ST_(CEU_GIH), high *F*_ST_(CEU_YRI) and low *F*_ST_(GIH_YRI). Since we did not know *a priori* the appropriate levels of *F*_ST_ similarity/dissimilarity that would enable us to detect genes associated with specific phenotypes, we used different *F*_ST_ thresholds to yield different numbers of ET SNPs. We first chose a highly stringent threshold, with the 95th percentile of *F*_ST_ indicating high differentiation and the 5th indicating a high degree of similarity (Fig. 2). This allowed us to limit the number of ET SNPs suitable for further hand-curated genetic association mining. We then applied more lenient thresholds, namely the 90th percentile and the 85th percentile reflecting high differentiation, and the corresponding 10th and 15th percentile reflecting sufficient similarity. ET genes were defined as genes that were within 100 Kb upstream or downstream of each ET SNP, according to NCBI Build 37. To further explore putative associating loci we also examined the recombination landscape around ET detected SNPs, using Locus Zoom ((http://locuszoom.sph.umich.edu/locuszoom/).

### Genetic association mining and identification of additional phenotypes

Genetic associations of ET genes with diseases were analyzed based on data retrieved from the HuGE Navigator [6] (http://www.cdc.gov/genomics/hugenet/hugenavigator.htm), a human genome epidemiology knowledgebase that incorporates information from PubMed abstracts as its core data source. We defined a “true” association as one for which a gene had: (1) five or more HuGE publications significantly associating it with a phenotype using the Genopedia function and *p* < 0.05 in any combination of studied populations, or (2) GWAS evidence with a *P* value of less than 5 × 10^−8^ reported in HuGE Navigator. Although negative GWAS results have been shown to correlate with geography and *F*_ST_ [7], we chose to be conservative, ignoring negative results as this is a proof of principle.

Additional phenotypes were noted if they too associated with ET genes in the HuGE Navigator database. Other associating diseases were then assessed for their global distributions to assess whether they are also similarly distributed to the index phenotypes (Additional file 1: Table S1).

### Random resampling analysis

To determine whether ET significantly enriched for genes that associated with the index or other appropriately distributed phenotypes, we performed permutation testing. Specifically, for each ET threshold we sampled from the genome the same number of genes that were identified to be associated with appropriately distributed disease using ET. We then verified using the same criteria as for ET how many of these genes were associated with appropriately distributed disease based on the continental ancestry for a given comparison. We used the empirical distribution to determine the *p*- value of the ET analyses. The empirical distributions were determined by sampling the genome 10,000 times without replacement. We established the number of times that the same number or more such genes were found by this random process.

## Results

ET comparisons among populations were chosen to reflect the distribution of several phenotypes, ranging from genetically simple to increasingly complex, that differ in prevalence among HapMap populations. Estimated genetic complexity was based on the number of GWAS identified genes and their effects sizes, extracted from the NHGRI GWAS catalog (Additional file 2: Table S2). The index phenotypes we analyzed were, in order of presumed increasing genetic complexity, lactase persistence, melanoma, and Type 2 diabetes mellitus/fasting glucose. These phenotypes are likely related to evolutionary adaptations to different environments, making the integration of evolutionary data more promising.

### Lactase persistence

Lactase persistence is the ability to digest lactose in adulthood and is the inverse of lactose intolerance [8]. More than 90 % of Northern European individuals have lactase persistence. In contrast, about half of the population of Southern Europe, and most Asians and Africans, have lactase persistence, as milk has been rarely consumed in these regions (Additional file 1: Table S1). Evolutionary evidence indicates that there has been strong selective pressure in populations with animal domestication and adult milk consumption to maintain the ability to metabolize lactose after weaning [9].

The genetics of lactase persistence is relatively simple. The primary gene that affects the phenotype is lactase (*LCT*) in the *LCT*-*MCM6* locus on chromosome 2 [8], and the identified variations are primarily *cis*-acting elements [10]. Interestingly, the variants associated with lactase persistence in European and African pastoralist populations are different, even though they are within the same upstream region of *LCT* [9]. In both cases, the evidence indicates that all variants are under strong selection corresponding to levels of animal milk consumption and pastoralist culture.

**Fig. 3:**
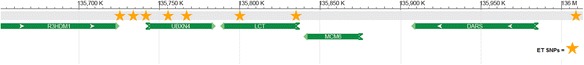
ET SNPs in vicinity of the *LCT* gene. Seven out of eight ET SNPs generated under the 95th/5th percentile threshold are within 100 Kb of *LCT* loci. The eighth is to the right of the *DARS* gene, upstream of the *LCT*-*MCM6* locus. (Coordinate only shows relative distance not indicating exact build 37 coordinates)

We applied ET to lactase persistence using three HapMap populations, CEU, TSI and CHB. Using the 95th percentile threshold to define highly differentiated SNPs between CEU/TSI and CEU/CHB, and the 5th percentile threshold to define similarly distributed SNPs between TSI and CHB, we identified eight ET SNPs and eight ET genes (Additional file 3: Table S3a). Seven of these SNPs were within ± 100 Kb of the *LCT*-*MCM6* region and, although more than 100 kb from *LCT*, the eighth was in the same region (Fig. 3). Of note, the majority of the ET SNPs were bounded by distal recombination hotspots, illustrating how we might refine locus boundaries (Additional file 4: Figure S1).

To assess the sensitivity of ET to varying levels of *F*_ST_ thresholds, we used multiple thresholds ranging from the 80th/20th to the 90th/10th percentile. The significant over-representation of the *LCT*-*MCM6* signal still held under less stringent thresholds. Under the 90th/10th threshold, we obtained 29 ET SNPs and 14 genes (Additional file 3: Table S3b). Twenty of these SNPs were within ± 100 Kb of *LCT*-*MCM6* region. Under the 85th/15th threshold, we generated 33 ET SNPs and 14 genes (Additional file 3: Table S3c). Again, twenty of these SNPs were within ± 100 Kb of *LCT*-*MCM6* region. Even using the 80th percentile threshold, with *F*_ST_(CEU_CHB) and *F*_ST_(CEU_TSI), and the 20th percentile threshold, with *F*_ST_(TSI_CHB), we generated 50 ET SNPs, of which 23 were within ± 100 Kb of *LCT*-*MCM6* region. (Additional file 3: Table S3d). *F*_ST_ values for other thresholds and populations are presented in Additional file 5: Table S4.

Based on the prevalence of lactase persistence in other populations [11] (Additional file 1: Table S1), we also applied ET to CEU, YRI and GIH. Highly differentiated SNPs between CEU and GIH and between CEU and YRI were compared to highly similar SNPs between GIH and YRI. With the 95th/5th threshold, we identified 22 SNPs, of which two were within ± 100 Kb of the *LCT*-*MCM6* region (Additional file 6: Table S5). Additionally, using the 95th/5th threshold, we were able to identify the *LCT*-*MCM6* gene region in comparisons of CEU to CHB and LWK (HapMap East African), both of which do not have a large amount of lactase persistence (data not shown).

### Two way comparison for lactase persistence

**Fig. 4:**
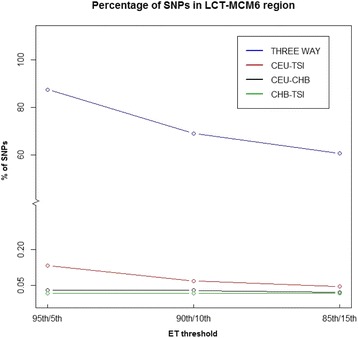
Percentage of ET SNPs within ± 100 Kb of the *LCT*-*MCM* genic region. In all two-way comparisons, only a small proportion (<0.2 %) of the SNPs are within ± 100 Kb of the *LCT*-*MCM* region. For example, the 95th percentile *F*_ST_ threshold of two way comparison between CEU and TSI (red line) generates 68,106 SNPs, only 91 (0.133 %) of which locate within ± 100 Kb of the *LCT*-*MCM* region. In contrast, signals from the three way comparison of *F*_ST_ (ET) more than 60 % of the ET SNPs are within the same region (blue line). The black line represents the pairwise comparisons between CEU and CHB, while the green line represents between CHB and TSI

As we hypothesized that adding a third population will improve the efficiency of filtering for disease related variants, we investigated the number of genes revealed by only examining two-way comparisons to assess the improved precision derived from adding a third population. To do this, we compared the percentage of SNPs that are within ±100 Kb of the *LCT*-*MCM* locus in all two-way *F*_ST_ comparisons vs. the three-way *F*_ST_ comparison (ET). Comparing only CEU to TSI under *F*_ST_ thresholds, ranging from 95th/5th to 85th/15th, we found that for two-way *F*_ST_ comparisons at best 0.13 % of the generated signals were located within the *LCT*-*MCM* region (95th/5th comparison). However, in the three-way *F*_ST_ comparison, more than 60 % of the ET SNPs are located within ±100 Kb of the *LCT*-*MCM* region (Fig. 4). Adding a third population greatly enriched the proportion of variants related to the corresponding phenotype(s), demonstrating that the ET can better resolve associating SNPs/genes.

### Melanoma and other diseases correlated with latitude

Melanoma was our second selected index phenotype. About 40 genes have been associated with melanoma, according to HuGE Navigator, making it substantially more complex than lactase persistence. The prevalence of melanoma varies with latitude [12], a relationship likely due to dark skin being favored in regions of high UV radiation, where it protects from skin damage, while still allowing adequate vitamin D to be synthesized. At the same time, dark skin limits UV degradation of folate in underlying skin layers, and low folate levels are associated with increased risk of neural tube defects in utero [13]. In contrast, light skin is more common at high latitudes where its presence promotes vitamin D biosynthesis in environments with low ultraviolet radiation [14]. Melanoma is relatively common in populations of European descent, but its prevalence is much lower in populations of South Asian and African descent (Additional file 1: Table S1). Thus, applying ET analysis to melanoma using the CEU, YRI and GIH populations (with CEU as the outlier population) fits the population prevalence requirements for ET filtering.

Under the stringent 95th/5th percentile threshold we obtained 22 ET SNPs that mapped to 33 ET genes (Additional file 6: Table S5a). One of these 33 genes, *SLC45A2*, has previously been shown to associate with melanoma [15]. The ET SNP that identified this locus, rs28117, is bounded both proximally and distally by recombination hotspots, thereby providing additional resolution (Additional file 7: Figure S2). Under the 90th/10th percentile threshold, we generated 168 ET SNPs which mapped to 230 ET genes. Under the least stringent 85th/15th percentile threshold, we generated 733 ET SNPs that mapped to 971 ET genes, four of which are known to associate with melanoma (Table 1, Additional file 6: Table S5b, S5c).

This analysis also identified numerous other genes associated with diseases that are distributed appropriately among these three populations. One example is multiple sclerosis (MS), a complex disease with more than 80 genes that have been associated with susceptibility, but each of these variants confers only a very small change in risk. Collectively, these genes explain approximately 20 % of the genetics of MS [16, 17]. Similar to melanoma, the prevalence of MS is associated with latitude; the most prominent correlating factor is ultraviolet radiation/vitamin D [18]. Recently, by showing positive selection on MS associated loci, researchers proposed that MS might have arisen due to pleiotropic effects of host resistance to pathogens over the course of human history, where there has been significant selective pressure acting to increase this resistance to pathogens [19]. Under the 90th/10th percentile threshold, we were able to identify one gene, *IL6*, previously associated with MS. *IL6* is of particular interest as it has been associated with 15 diseases that are similarly distributed, several of which are related to immune function, indicating strong pleiotropic effects (Additional file 8: Table S6).

The ET analysis for melanoma also identified genes associating with many other similarly distributed phenotypes (Additional file 8: Table S6). For example, ET detected *LCT* under the most stringent 95th/5th *F*_ST_ percentile threshold, consistent with the distribution of lactase persistence. Besides lactase persistence, ET genes under the least stringent 85th/15th threshold have also been associated with other Mendelian disorders having appropriate distributions among the three populations, such as oculocutaneous albinism, glucosephosphate dehydrogenase deficiency and Smith-Lemli-Opitz Syndrome. ET was able to capture the key genes that cause these diseases, namely *OCA2*, *G6PD* and *DHCR7*. Other disorders have also been associated with these ET genes, but the association is not as direct. For example, *G6PD* has been associated with favism caused by G6PD deficiency that can affect survival of the malaria parasite, *Plasmodium falciparum* [20]. Malaria is much more common in South Asia and Africa than it is in northern Europe, highlighting another appropriately identified genetic association. ET was also able to capture genes that have been associated with complex diseases having appropriate distributions among the three populations, for example, Vitamin D deficiency, which is strongly correlated with skin color. *DHCR7* and *CYP24A1* have the largest reported effect sizes for Vitamin D deficiency and were found using ET [21]. Interestingly, another ET gene, *BNC2*, has also been associated with hair and skin color in a GWAS study [22]. As melanoma is strongly associated with skin color, we expect that genes affecting skin color would also appear. The evolutionary factors that drive these phenotypes are likely common, emphasizing the ability of ET to detect genes that are pleiotropic. ET genes have also been associated with other complex diseases, such as pulmonary tuberculosis, multiple cancers, and Alzheimer’s disease, all of which have relatively large effect sizes (Additional file 8: Table S6). ET comparisons also allowed us to identify key immune regulators, such as *IFNG* and *IL6*, which associate with many phenotypes.

### Type 2 diabetes mellitus/fasting glucose

Fasting glucose, although measured as a continuous trait, is reflective of Type 2 diabetes mellitus prevalence. Because diabetes case criteria may differ among studies based on variable diagnostic standards we chose to use fasting glucose as our index phenotype, as it is a more objective but highy correlated trait representing the complex genetic architecture of Type 2 diabetes. In the Mexican population, the average fasting glucose level is higher than in Asian and Northern European populations. For this analysis, we used the populations MEX, JPT, and CEU for ET analyses based on current epidemiological data.

We identified no genes that have been associated with Type 2 diabetes mellitus, according to our criteria. Because of this inability to identify appropriate genes we expanded our search to include genes that were within 250 Kb of each ET SNP. Although we discovered no genes associated with Type 2 diabetes mellitus using the most stringent threshold, we did find one gene with the 90th/10th threshold, *BHMT*, that has been putatively associated with diabetes (β = 0.26; *p* value 5.9 × 10^−3^) [23].

Under the 95th/5th percentile threshold, we found only seven ET SNPs which mapped to four genes. None of these four genes had evidence of genetic association with any phenotype, to date. Using the 90th/10th percentile threshold, we identified 164 ET SNPs that mapped to 115 genes. Again, none of these genes were associated with any disease having the expected population distribution. Under the 85th/15th percentile threshold, we identified 407 ET SNPs that mapped to 233 genes. Of these genes, two had prior evidence of association with diseases with the appropriate population distributions. Both genes, *XG* and *NLGN4X*, are related to autism spectrum disorders that are also appropriately distributed among these populations (Additional file 1: Table S1). The *p* value of the most significant SNP in *XG* was 3.79 × 10^−8^ in a family based GWAS [24]. In contrast, in *NLGN4X* the smallest *p *value was 0.024 in a candidate gene study [25]. We also found a gene, *SHROOM2*, that is associated with an Alzheimer’s disease endophenotype, but the GWAS study describing this association only generated a *p* value of 0.0003 [26].

### Enrichment of ET genes relative to randomly sampled genes

To assess whether ET significantly enriched for genes associating with our index and other appropriately distributed phenotypes, we perfomed random resampling of the genome to estimate an empiral *p* value. We then interrogated HuGE Navigator to identify associations and tested whether our randomly sampled genes were associated with either our index phenotypes or other traits similarly distributed among the respective populations. From the CEU, GIH and YRI ET analyses for melanoma, for the most stringent threshold, four out of the 33 identified genes were known to be associated with phenotypes appropriately distributed among the three populations (Table 2). For the next two thresholds, the results were 15 out of 230 genes and 49 out of 971 genes. Our random resampling determined in all three of these cases that ET significantly enriched for genes that associated with appropriately distributed diseases/traits, with the *p*-value being more significant for the less stringent thresholds (Table 2).

**Table 2:** Significant Association of ET Genes (CEU, GIH, YRI) with diseases/traits

Percentile threshold	Number of ET SNPs	Number of ET genes	Number of diseases appropriately distributed among ET populations	Number of ET genes associated with appropriately distributed diseases	Permutation *P* value
95th/5th	22	33	6	4	0.0237
90th/10th	168	230	27	15	0.0111
85th/15th	733	971	42	49	0.0023

### Comparison to population branch statistic

An existing method, Population Branch Statistic (PBS) also integrates evolutionary comparisons among three populations to search for candidate genes and has been successful in identifying genes related to high altitude adaptation [27]. PBS uses two populations with known differences in phenotype prevalence and a third random outlier population to identify genes likely to associate with a predefined trait. Variants that show high allele frequency differentiation in only one of the two pre-selected populations are assumed to be under selection, reflected by high PBS scores. Unlike our method, there is no phenotype prevalence restriction on the outlier population. We compared ET with PBS to determine if adding the phenotypic prevalence in the third population increases our ability to find disease associating genes. To do this, we first chose CEU and YRI as the two pre-selected populations, then ran PBS analysis using all other available HapMap III populations as the outlier. We selected the top 22 SNPs from PBS (based on the most stringent CEU-YRI-GIH comparison from ET) to assess the performance against ET. SNPs with the highest PBS scores were picked for each population and then were mapped to genes within ± 100 Kb (Additional file 9: Table S7). To determine association, we applied the same criteria as described above for our ET analyses. We found that, at most, three genes, *SLC12A1*, *SLC45A2* and *AMACR*, are associated with phenotypes that are appropriately distributed between European and West African populations, two of which had been observed using ET. In other analyses using MEX, TSI, GIH, ASW, LWK and MKK as outlier populations, only one appropriate gene was identified, *SLC12A1*. Using CHD and JPT as outlier populations, we identified no genes associated with differentially distributed disease rates. In contrast, ET was able to identify four genes relevant to diseases (Table 2). It is likely that ET performed better in most cases because we explicitly added a third population with known disease prevalences and this additional information appeared to increase our resolving power.

## Discussion

### ET’s performance is related to effect size

In this manuscript, we addressed whether population differences shaped by evolutionary histories can be used to identify or enrich for genes associated with phenotypes that are differentially distributed. We hypothesized that comparing three populations, using the ET algorithm, can enrich for variants or genes that can be compared to standard association results, helping define those of greater interest with respect to phenotype. This, in essence, is an additional filtering method that may allow us to relax standard association significance thresholds, such that we can identify putative disease associating variants that can be targeted for follow-up, even if they do not reach standard genome-wide association significance. In our analyses, we focused on three different phenotypes that are both differentially distributed and have putatively different genetic architectures. ET’s performance appeared to differ among these phenotypes. For the trait with the simplest genetic architecture, lactase persistence, ET was robust; ET defined the causative genetic region using several population comparisons that matched the distribution of lactase persistence [9, 28]. For likely oligogenic phenotypes such as melanoma, ET performed well. Assuming that the number of GWAS hits in each of these two diseases is correlated with the complexity of the genetic models (one for lactase persistence and 22 for melanoma), we can see that as the effect sizes decrease and the number of associating genes increases, which, presumably, is correlated with increasing complexity of the genetic architecture, ET was less effective in identifying previously known genes [29, 30]. It may also be possible that ET is best suited to detect loci that may not only be associating with diseases that are differentially distributed, but with loci that differentiated due to selection, as is the case with genes for lactase persistence and melanoma. Interestingly, standard GWAS found multiple signals for lactase persistence, but after adjusting for ancestry all except *LCT*, which was identified by ET, became non-significant, demonstrating that ET does not conflate ancestry with association [29].

Regardless of the *F*_ST_ threshold, ET clearly showed an enrichment for melanoma genes compared to the random resampling analysis, emphasizing its utility. It is also interesting that ET did significantly enrich for genes associated with several other diseases that shared the same prevalence pattern and are presumably related to melanoma. Multiple sclerosis, although sharing the same geographic distribution as melanoma, does not appear to be as good a candidate disease for ET analysis. However, MS appears to be more genetically complex based on the number and effect sizes of genes already identified as associating with it (Additional file 2: Table S2). Although we did identify some genes related to MS, we were not able to see an enrichment for them. MS also appears to be more affected by environmental variation, and risk can change with migration during early adolescence, thereby serving as a modifier of genetic risk [31]. Another important finding from ET in the melanoma analyses is the identification of G6PD. This is of particular interest as G6PD deficiency is known to protect from malarial infection [20, 32–34], although it was not found in the GWAS for malaria [35]. We do, however, note that the failure to identify G6PD in the original malaria GWAS is likely an artifact of poor coverage in this genic region as well as low frequency of the protective alleles in the studied populations. Nonetheless, this demonstrates the potential of ET, to not only refine, but in some cases, to identify important signals missed by standard association that are included in publically available databases but absent from genotyping arrays.

For phenotypes that are polygenic, with putative small effect sizes such as fasting glucose or Type 2 diabetes mellitus, ET’s utility appeared to be more limited. Specifically, the role of environment in confounding the ability of ET to identify risk genes may be seen in diabetes, as the prevalence of Type 2 diabetes is increasing, presumably because of the adoption of lifestyles that promote it. Therefore, even if there are strong diabetes genes in a few environments, their action may not be detectable in many modern human societies where the effect of the environment has become preeminent and time has been inadequate to differentiate genes affecting this phenotype. We may, therefore, conclude that as the environment’s recent role in disease increases, the ability of ET to identify key genes will decrease. This should correlate with the length to which a population has been exposed to a new environment. This is not a feature unique to ET, as this will be the case even with standard association approaches. Nonetheless, even though ET and standard association methods differ substantially in their underlying assumptions and metrics, they may still be used to support each other [36]. In cases where there is significant locus heterogeneity among cohorts, ET may be extremely useful in helping to identify interesting associations in subsets of data that are often ignored in large GWAS meta-analyses. Therefore, despite that fact that ET did not enrich for known diabetes genes, it may have enriched for previously undetected diabetes genes, and can be used to provide additional evidence for follow-up. As with other approaches, ET is not as useful for detecting variants that are rare in all populations, as association offers low power in detecting differences. However, we emphasize that all current population-based designs suffer from this limitation and are unlikely to be practical for the statistical detection of rare variants that associate with disease. Family based (linkage) approaches to identifying rare disease associated genes are still the preferred method in these cases.

### ET as an agnostic filter

GWAS have been used to examine almost every common complex disease, and although many SNPs have been dismissed because they do not meet standard multiple testing correction thresholds, it is probable that many of them truly associate with the disease. The detection of these SNPs represents a major challenge in our developing understanding of disease etiology because the current methods produce inflated Type II errors. Thus, although GWAS have been able to discover significant hits in certain diseases, its success is limited in most situations and probably represents the limitations of only using *p* values for biological data mining [37], making association result filtering based solely on *p* value problematic. Therefore, it is important to develop methods that can minimize this type of error while still controlling for the Type I error rate. One way to do this is to integrate knowledge from independent analyses [36]. Integration of several data types has been successful in the analysis of diseases, such as MS [38]. However, pathway-oriented filtering, such as for MS in Baranzini et al., requires prior knowledge of biological functions, which are still poorly understood in many cases. We present an alternative filtering metric based, not on prior biological knowledge, only on evolutionary history that shaped biology. ET also has an advantage over some standard association analyses because, if a variant that increases risk is fixed in a population that has a higher prevalence (i.e., is an etiological agent), it would not be detected in association analyses; however, ET may identify it. A key to the success of ET is having at least three populations, since comparing only two would produce an enormous number of false positives, while adding a third population can reduce this number.

ET only requires knowledge of allele frequencies and disease distributions, but the latter may be a limiting factor for many diseases, but this will change with better surveillance. However, limited surveillance is not the only factor that may affect the utility of ET. For example, variable diagnoses across countries may present an issue as will changing definitions of disease phenotypes over time. The latter will make comparisons over time difficult. The issue of ethnic variation in disease prevalence may, however, be partially ameliorated in coutries that have highly diverse populations, such as the US where data on many continental populations can be collected simultaneously and used to define appropriate ET populations, but this is still not as robust as would be ideal as many populations in such situations are highly admixed. As ET detects differences in local variation the method should still be powerful for the detection of genes that affect disease risk in all populations, i.e. universal genetic risk factors. However, in epistatic models of disease it may not be possible to detect all key genes when local ancestry varies elsewhere in the genome [39].

### Combining F_ST_ with other metrics

In this study, we used only *F*_ST_ as a metric of population differentiation. *F*_ST_ is computationally straightforward and thus makes our model easily applied in any population comparisons. Since genetic differentiation could be due to both selection and random drift, and *F*_ST_ does not explicitly differentiate between these two processes, extensions of ET that include measures of selection may be useful for future analyses. An existing statistic that can be used to provide evidence for selection is iHS [40] (http://haplotter.uchicago.edu/). We investigated iHS patterns in the genomic regions identified in our study using the most stringent *F*_ST_ thresholds among CEU, YRI and GIH. In most regions the iHS scores were larger in CEU than in YRI and ASN (the East Asian HapMap population), indicating stronger selection signals in the CEU population (Additional file 10: Table S8). Clearly, integrating the role of selection into the ET analyses in the future will be an important extension of the method.

Selection may be an important factor in shaping the distribution of disease risk alleles; however, it is not always the mechanism through which important differentiation, which affects phenotypic variation, takes place. One strong example of this is the *LCT* locus, which has been shown by multiple metrics to be under selection in multiple populations and was identified by ET in our study [9, 41]. Another example of a selected locus affecting disease risk that was identified by ET is *APOL1*. This gene has been shown to be under selection in African populations, due to protection against human African trypanosomiasis [42]. Different metrics may be able to pick up different signals indicating selection (e.g., Cross Population Extended Haplotype Homozygosity (XP-EHH) [43], Tajima’s D [44], Fay and Wu’s H [45]). In contrast to the phenotypes with comparatively simple genetic architecture in which selection appears to be important in shaping population structure, the role of selection in Type 2 diabetes mellitus is less clear. Recent studies of Type 2 diabetes mellitus genes have indicated that they are not under selective pressure among multiple human populations [46, 47]. However, another paper studying the distribution of Type 2 diabetes mellitus risk alleles indicates that random drift alone has not shaped diabetes’ genetic risk differences from Africa to East Asia [48], making it difficult to draw strong conclusions at this point. Understanding the role of non-random processes in shaping disease risk will be an important area of research, especially as it affects disease disparity among continental populations in changing environments.

### Possible pleiotropy in ET genes

In our analyses of ET genes and their associated diseases, it became obvious that several of the genes identified are risk factors for multiple diseases. Among the genes from the least stringent ET threshold among CEU, YRI and GIH, we discovered that several genes were associated with multiple diseases and traits (Tables 1, Additional file 8: Table S6). For example, *IL6* has been associated with 15 phenotypes that are appropriately distributed and *IFNG* with seven. Both genes are related to immune response, among other processes. It is possible that selection has been important in changing allele frequency distributions, while at the same time having multiple pleiotropic effects that may or may not have been directly driven by selection. Another example is the genes related to melanoma, as they are also associated with skin color, eye color and albinism (*SLC45A2* [49, 50]), preterm birth (*DHCR7* [51]), and Vitamin D levels (*NADSYN1* [21]). Interestingly, an ET gene from the most stringent threshold, *BNC2*, has also been associated with hair and skin color [22, 52, 53]. In this specific case, it seems that skin color is a confounder of other genetic effects. As humans migrated to higher latitudes with insufficient UV radiation, lighter skin color facilitated more vitamin D synthesis in the skin. However, this increased the risk of melanoma. Although having less than five publications indicating associations, *BNC2* has also been associated with ovarian cancer in two GWAS, both reaching genome wide significance [54, 55]. We do note that our calculations of the number of associating genes, with appropriately distributed phenotypes, were conservative; each gene was counted only once, even if it associated with multiple pleiotropic phenotypes.

### Comparisons to other related methods

Models developed in different studies employ similar evolutionary mindsets to what we have discussed. PBS does not require a specific phenotype prevalence pattern on the third population in the three population comparison, which may make it less powerful than ET in identifying disease associating genes. On the other hand, Hancock et al. [56] reported that correlations between allele frequencies and climate variables can be applied to detect SNPs associating with pigmentation and autoimmune diseases. In this case, they were looking for local selection due to environmental factors chosen *a priori*, whereas ET is not limited to specific factors that give rise to the disease prevalence distribution pattern.

Another method was developed to identify loci covarying with the African Pygmy phenotype based on how much additional information is provided by phenotype to infer the geographic origin compared to information provided without genotypes [57]. However, this method requires the phenotype, which is height, to be known for every individual. In our method, we only need to compare disease risk at the population level.

## Conclusions

Evolutionary thinking can provide important insights for biomedical research; when combined with current approaches commonly employed in human disease studies, it can increase our ability to find key genes or pathways that affect etiology. By taking advantage of both epidemiological differences and population structure, we demonstrated that many genes associating with diseases can be found. This paper presents a proof of principle for this approach, as well as some of its limitations. Clearly, for phenotypes with simple genetic architecture, ET is an extremely powerful approach, but this becomes less practical for traits of increasingly complex architecture. Nonetheless, for several traits, we were able to identify genes with known effects. It is also possible that many more of the ET genes are truly associating, but have not been reported as such, as they do not meet current thresholds for significance. Therefore, we propose that ET can be a useful filter with which to interrogate existing and new association studies for consistent patterns that might lead to the identification of additional genetic risk factors.

## Additional files

Additional file 1: Table S1. Prevalence of phenotypes among continental populations as defined by HapMap samples. (DOCX 23 kb) Additional file 2: Melanoma GWAS hits. Type 2 diabetes mellitus GWAS hits. Multiple sclerosis GWAS hits. (XLSX 54 kb) Additional file 3: Table S3a. SNPs and genes identified using the 95th/5th percentile ET cutoffs among CEU-TSI-CHB. **Table S3b.** SNPs and genes identified using the 90th/10th percentile ET cutoffs among CEU-TSI-CHB. **Table S3c.** SNPs and genes identified using the 85th/15th percentile ET cutoffs among CEU-TSI-CHB. **Table S3d.** SNPs and genes identified using the 80th/20th percentile ET cutoffs among CEU-TSI-CHB. (DOCX 26 kb) Additional file 4: Figure S1. Distribution of recombination hotspots surrounding ET SNP rs12615624, idenitifed by comparing CEU-TSI (high Fst; 95th percentile), CEU-CHB (high Fst; 95th percentile) and TSICHB (low Fst; 5th percentile). (PDF 15 kb) Additional file 5: Table S4. F_ST_ values at each of the percentile thresholds. (DOCX 14 kb) Additional file 6: Table S5a. SNPs and genes identified using the 95^th^/5^th^ percentile ET cutoffs among CEU-GIH-YRI. **Table S5b.** SNPs and genes identified using the 90^th^/10^th^ percentile ET cutoffs among CEU-GIH-YRI. **Table S5c.** SNPs and genes identified using the 85^th^/15^th^ percentile ET cutoffs among CEU-GIH-YRI. (DOCX 96 kb) Additional file 7: Distribution of recombination hotspots surrounding ET SNP rs28117 near melanoma associated gene SLC45A2. (PDF 15 kb) Additional file 8: Table S6. Genetic associations of 85^th^/15^th^ cutoff ET genes with diseases other than index diseases based on CEU-YRI-GIH comparisons^1^. (DOCX 145 kb) Additional file 9: PBS analysis using CEU and YRI as the pre-selected populations and other available HapMap III populations as the outlier populations genes associated with diseases that are appropriately distributed between CEU and YRI genes associated with diseases that are appropriately distributed among CEU, YRI and GIH and identifed with ET genes show up across all PBS analysis. (XLSX 14 kb) Additional file 10: Table S8. SNP-based iHS score of CEU-GIH-YRI ET SNPs under 95^th^/5^th^ percentile cutoff. (DOCX 15 kb)

## References

[1] Shriver MD, Kennedy GC, Parra EJ, Lawson HA, Sonpar V, Huang J, Akey JM, Jones KW. The genomic distribution of population substructure in four populations using 8,525 autosomal SNPs. Human genomics. 2004;1(4):274–86. 10.1186/1479-7364-1-4-274 PMC3525267 15588487

[2] Elhaik E (2012). Empirical distributions of F(ST) from large-scale human polymorphism data. PLoS One.

[3] Wright S (1968). Evolution and the genetics of populations; a treatise.

[4] International HapMap C, Altshuler DM, Gibbs RA, Peltonen L, Altshuler DM, Gibbs RA, Peltonen L, Dermitzakis E, Schaffner SF, Yu F, et al. Integrating common and rare genetic variation in diverse human populations. Nature. 2010;467(7311):52–8. 10.1038/nature09298 PMC3173859 20811451

[5] Weir BS, Cockerham C (1984). Estimating F-statistics for the analysis of population structure. Evolution.

[6] Yu W, Gwinn M, Clyne M, Yesupriya A, Khoury MJ (2008). A navigator for human genome epidemiology. Nat Genet.

[7] Marigorta UM, Lao O, Casals F, Calafell F, Morcillo-Suarez C, Faria R, Bosch E, Serra F, Bertranpetit J, Dopazo H et al. Recent human evolution has shaped geographical differences in susceptibility to disease. BMC Genomics. 2011;12:55. 10.1186/1471-2164-12-55 PMC3039608 21261943

[8] Swallow DM (2003). Genetics of lactase persistence and lactose intolerance. Annu Rev Genet.

[9] Tishkoff SA, Reed FA, Ranciaro A, Voight BF, Babbitt CC, Silverman JS, Powell K, Mortensen HM, Hirbo JB, Osman M (2007). Convergent adaptation of human lactase persistence in Africa and Europe. Nat Genet.

[10] Wang Y, Harvey CB, Pratt WS, Sams VR, Sarner M, Rossi M, Powell K, Mortensen HM, Hirbo JB, Osman M,et al. The lactase persistence/non-persistence polymorphism is controlled by a cis-acting element. Hum Mol Genet. 1995;4(4):657–62. 10.1093/hmg/4.4.657 7543318

[11] Itan Y, Jones BL, Ingram CJ, Swallow DM, Thomas MG (2010). A worldwide correlation of lactase persistence phenotype and genotypes. BMC Evol Biol.

[12] Crombie IK (1979). Racial differences in melanoma incidence. Br J Cancer.

[13] Jablonski NG, Chaplin G (2010). Colloquium paper: human skin pigmentation as an adaptation to UV radiation. Proc Natl Acad Sci U S A.

[14] Loomis WF (1967). Skin-pigment regulation of vitamin-D biosynthesis in man. Science.

[15] Barrett JH, Iles MM, Harland M, Taylor JC, Aitken JF, Andresen PA, Akslen LA, Armstrong BK, Avril MF, Azizi E, et al. Genome-wide association study identifies three new melanoma susceptibility loci. Nat Genet. 2011;43(11):1108–13. 10.1038/ng.959 PMC3251256 21983787

[16] International Multiple Sclerosis Genetics C, Wellcome Trust Case Control C, Sawcer S, Hellenthal G, Pirinen M, Spencer CC, Patsopoulos NA, Moutsianas L, Dilthey A, Su Z, et al. Genetic risk and a primary role for cell-mediated immune mechanisms in multiple sclerosis. Nature. 2011;476(7359):214–9. 10.1038/nature10251 PMC3182531 21833088

[17] International Multiple Sclerosis Genetics C, Beecham AH, Patsopoulos NA, Xifara DK, Davis MF, Kemppinen A, Cotsapas C, Shah TS, Spencer C, Booth D, et al. Analysis of immune-related loci identifies 48 new susceptibility variants for multiple sclerosis. Nat Genet. 2013;45(11):1353–60. 10.1038/ng.2770 PMC3832895 24076602

[18] Simpson S, Blizzard L, Otahal P, Van der Mei I, Taylor B (2011). Latitude is significantly associated with the prevalence of multiple sclerosis: a meta-analysis. J Neurol Neurosurg Psychiatry.

[19] Raj T, Kuchroo M, Replogle JM, Raychaudhuri S, Stranger BE, De Jager PL (2013). Common risk alleles for inflammatory diseases are targets of recent positive selection. Am J Hum Genet.

[20] Luzzatto L (2012). G6PD deficiency and malaria selection. Heredity.

[21] Ahn J, Yu K, Stolzenberg-Solomon R, Simon KC, McCullough ML, Gallicchio L, Jacobs EJ, Ascherio A, Helzlsouer K, Jacobs KB, et al. Genome-wide association study of circulating vitamin D levels. Hum Mol Genet. 2010;19(13):2739–45. 10.1093/hmg/ddq155 PMC2883344 20418485

[22] Eriksson N, Macpherson JM, Tung JY, Hon LS, Naughton B, Saxonov S, Avey L, Wojcicki A, Pe'er I, Mountain J. Web-based, participant-driven studies yield novel genetic associations for common traits. PLoS Genet. 2010;6(6):e1000993. 10.1371/journal.pgen.1000993 PMC2891811 20585627

[23] Xie W, Wood AR, Lyssenko V, Weedon MN, Knowles JW, Alkayyali S, Assimes TL, Quertermous T, Abbasi F, Paananen J, et al. Genetic variants associated with glycine metabolism and their role in insulin sensitivity and type 2 diabetes. Diabetes. 2013;62(6):2141–50. 10.2337/db12-0876 PMC3661655 23378610

[24] Chang SC, Pauls DL, Lange C, Sasanfar R, Santangelo SL (2013). Sex-specific association of a common variant of the XG gene with autism spectrum disorders. American journal of medical genetics Part B, Neuropsychiatric genetics : the official publication of the International Society of Psychiatric Genetics.

[25] Chakrabarti B, Dudbridge F, Kent L, Wheelwright S, Hill-Cawthorne G, Allison C, Banerjee-Basu S, Baron-Cohen S. Genes related to sex steroids, neural growth, and social-emotional behavior are associated with autistic traits, empathy, and Asperger syndrome. Autism research : official journal of the International Society for Autism Research. 2009;2(3):157–77. 10.1002/aur.80 19598235

[26] Meda SA, Narayanan B, Liu J, Perrone-Bizzozero NI, Stevens MC, Calhoun VD, Glahn DC, Shen L, Risacher SL, Saykin AJ, et al. A large scale multivariate parallel ICA method reveals novel imaging-genetic relationships for Alzheimer’s disease in the ADNI cohort. NeuroImage. 2012;60(3):1608–21. 10.1016/j.neuroimage.2011.12.076 PMC3312985 22245343

[27] Yi X, Liang Y, Huerta-Sanchez E, Jin X, Cuo ZX, Pool JE, Xu X, Jiang H, Vinckenbosch N, Korneliussen TS, et al. Sequencing of 50 human exomes reveals adaptation to high altitude. Science. 2010;329(5987):75–8. 10.1126/science.1190371 PMC3711608 20595611

[28] Enattah NS, Sahi T, Savilahti E, Terwilliger JD, Peltonen L, Jarvela I (2002). Identification of a variant associated with adult-type hypolactasia. Nat Genet.

[29] Price AL, Patterson NJ, Plenge RM, Weinblatt ME, Shadick NA, Reich D (2006). Principal components analysis corrects for stratification in genome-wide association studies. Nat Genet.

[30] Gerstenblith MR, Shi J, Landi MT (2010). Genome-wide association studies of pigmentation and skin cancer: a review and meta-analysis. Pigment Cell Melanoma Res.

[31] Ebers GC (2008). Environmental factors and multiple sclerosis. Lancet Neurol.

[32] Howes RE, Piel FB, Patil AP, Nyangiri OA, Gething PW, Dewi M, Hogg MM, Battle KE, Padilla CD, Baird JK, et al. G6PD deficiency prevalence and estimates of affected populations in malaria endemic countries: a geostatistical model-based map. PLoS Med. 2012;9(11):e1001339. 10.1371/journal.pmed.1001339 PMC3496665 23152723

[33] Tishkoff SA, Varkonyi R, Cahinhinan N, Abbes S, Argyropoulos G, Destro-Bisol G, Drousiotou A, Dangerfield B, Lefranc G, Loiselet J, et al. Haplotype diversity and linkage disequilibrium at human G6PD: recent origin of alleles that confer malarial resistance. Science. 2001;293(5529):455–62. 10.1126/science.1061573 11423617

[34] Bienzle U, Ayeni O, Lucas AO, Luzzatto L (1972). Glucose-6-phosphate dehydrogenase and malaria. Greater resistance of females heterozygous for enzyme deficiency and of males with non-deficient variant. Lancet.

[35] Jallow M, Teo YY, Small KS, Rockett KA, Deloukas P, Clark TG, Kivinen K, Bojang KA, Conway DJ, Pinder M, et al. Genome-wide and fine-resolution association analysis of malaria in West Africa. Nat Genet. 2009;41(6):657–65. 10.1038/ng.388 PMC2889040 19465909

[36] Ciesielski T, Pendergrass S, White M, Kodaman N, Sobota R, Huang M, Bartlett J, Li J, Pan Q, Gui J, et al. Diverse convergent evidence in the genetic analysis of complex disease: coordinating omic, informatic, and experimental evidence to better identify and validate risk factors. BioData mining. 2014;7(1):10. 10.1186/1756-0381-7-10 PMC4112852 25071867

[37] Malley JD, Dasgupta A, Moore JH (2013). The limits of p-values for biological data mining. BioData mining.

[38] Baranzini SE, Galwey NW, Wang J, Khankhanian P, Lindberg R, Pelletier D, Wu W, Uitdehaag BM, Kappos L, Gene MSAC et al. Pathway and network-based analysis of genome-wide association studies in multiple sclerosis. Hum Mol Genet. 2009;18(11):2078–90. 10.1093/hmg/ddp120 PMC2678928 19286671

[39] Greene CS, Penrod NM, Williams SM, Moore JH (2009). Failure to replicate a genetic association may provide important clues about genetic architecture. PLoS One.

[40] Voight BF, Kudaravalli S, Wen X, Pritchard JK (2006). A map of recent positive selection in the human genome. PLoS Biol.

[41] Wagh K, Bhatia A, Alexe G, Reddy A, Ravikumar V, Seiler M, Boemo M, Yao M, Cronk L, Naqvi A, et al. Lactase persistence and lipid pathway selection in the Maasai. PLoS One. 2012;7(9):e44751. 10.1371/journal.pone.0044751 PMC3461017 23028602

[42] Ko WY, Rajan P, Gomez F, Scheinfeldt L, An P, Winkler CA, Froment A, Nyambo TB, Omar SA, Wambebe C, et al. Identifying Darwinian selection acting on different human APOL1 variants among diverse African populations. Am J Hum Genet. 2013;93(1):54–66. 10.1016/j.ajhg.2013.05.014 PMC3710747 23768513

[43] Sabeti PC, Varilly P, Fry B, Lohmueller J, Hostetter E, Cotsapas C, Xie X, Byrne EH, McCarroll SA, Gaudet R, et al. Genome-wide detection and characterization of positive selection in human populations. Nature. 2007;449(7164):913–8. 10.1038/nature06250 PMC2687721 17943131

[44] Tajima F (1989). Statistical method for testing the neutral mutation hypothesis by DNA polymorphism. Genetics.

[45] Fay JC, Wu CI (2000). Hitchhiking under positive Darwinian selection. Genetics.

[46] Southam L, Soranzo N, Montgomery SB, Frayling TM, McCarthy MI, Barroso I, Zeggini E (2009). Is the thrifty genotype hypothesis supported by evidence based on confirmed type 2 diabetes- and obesity-susceptibility variants?. Diabetologia.

[47] Ayub Q, Moutsianas L, Chen Y, Panoutsopoulou K, Colonna V, Pagani L, Prokopenko I, Ritchie GR, Tyler-Smith C, McCarthy MI, et al. Revisiting the thrifty gene hypothesis via 65 loci associated with susceptibility to type 2 diabetes. Am J Hum Genet. 2014;94(2):176–85. 10.1016/j.ajhg.2013.12.010 PMC3928649 24412096

[48] Corona E, Chen R, Sikora M, Morgan AA, Patel CJ, Ramesh A, Bustamante CD, Butte AJ. Analysis of the genetic basis of disease in the context of worldwide human relationships and migration. PLoS Genet. 2013;9(5):e1003447. 10.1371/journal.pgen.1003447 PMC3662561 23717210

[49] Beleza S, Johnson NA, Candille SI, Absher DM, Coram MA, Lopes J, Campos J, Araujo, II, Anderson TM, Vilhjalmsson BJ, et al. Genetic architecture of skin and eye color in an African-European admixed population. PLoS Genet. 2013;9(3):e1003372. 10.1371/journal.pgen.1003372 PMC3605137 23555287

[50] Stokowski RP, Pant PVK, Dadd T, Fereday A, Hinds DA, Jarman C, Filsell W, Ginger RS, Green MR, van der Ouderaa FJ, et al. A genomewide association study of skin pigmentation in a South Asian population. Am J Hum Genet. 2007;81(6):1119–32. 10.1086/522235 PMC2276347 17999355

[51] Bream EN, Leppellere CR, Cooper ME, Dagle JM, Merrill DC, Christensen K, Simhan HN, Fong CT, Hallman M, Muglia LJ, et al. Candidate gene linkage approach to identify DNA variants that predispose to preterm birth. Pediatr Res. 2013;73(2):135–41. 10.1038/pr.2012.166 PMC3740714 23168575

[52] Visser M, Palstra RJ, Kayser M (2014). Human skin color is influenced by an intergenic DNA polymorphism regulating transcription of the nearby BNC2 pigmentation gene. Hum Mol Genet.

[53] Jacobs LC, Wollstein A, Lao O, Hofman A, Klaver CC, Uitterlinden AG, Nijsten T, Kayser M, Liu F. Comprehensive candidate gene study highlights UGT1A and BNC2 as new genes determining continuous skin color variation in Europeans. Hum Genet. 2013;132(2):147–58. 10.1007/s00439-012-1232-9 23052946

[54] Goode EL, Chenevix-Trench G, Song H, Ramus SJ, Notaridou M, Lawrenson K, Widschwendter M, Vierkant RA, Larson MC, Kjaer SK, et al. A genome-wide association study identifies susceptibility loci for ovarian cancer at 2q31 and 8q24. Nat Genet. 2010;42(10):874–9. 10.1038/ng.668 PMC3020231 20852632

[55] Song H, Ramus SJ, Tyrer J, Bolton KL, Gentry-Maharaj A, Wozniak E, Anton-Culver H, Chang-Claude J, Cramer DW, DiCioccio R, et al. A genome-wide association study identifies a new ovarian cancer susceptibility locus on 9p22.2. Nat Genet. 2009;41(9):996–1000. 10.1038/ng.424 PMC2844110 19648919

[56] Hancock AM, Witonsky DB, Alkorta-Aranburu G, Beall CM, Gebremedhin A, Sukernik R, Utermann G, Pritchard JK, Coop G, Di Rienzo A. Adaptations to climate-mediated selective pressures in humans. PLoS Genet. 2011;7(4):e1001375. 10.1371/journal.pgen.1001375 PMC3080864 21533023

[57] Mendizabal I, Marigorta UM, Lao O, Comas D (2012). Adaptive evolution of loci covarying with the human African Pygmy phenotype. Hum Genet.

[58] Yoshizawa J, Abe Y, Oiso N, Fukai K, Hozumi Y, Nakamura T, Narita T, Motokawa T, Wakamatsu K, Ito S, et al. Variants in melanogenesis-related genes associate with skin cancer risk among Japanese populations. J Dermatol. 2014;41(4):296–302. 10.1111/1346-8138.12432 24617981

[59] Gudbjartsson DF, Sulem P, Stacey SN, Goldstein AM, Rafnar T, Sigurgeirsson B, Benediktsdottir KR, Thorisdottir K, Ragnarsson R, Sveinsdottir SG, et al. ASIP and TYR pigmentation variants associate with cutaneous melanoma and basal cell carcinoma. Nat Genet. 2008;40(7):886–91. 10.1038/ng.161 18488027

[60] Nelson HH, Kelsey KT, Mott LA, Karagas MR (2002). The XRCC1 Arg399Gln polymorphism, sunburn, and non-melanoma skin cancer: evidence of gene-environment interaction. Cancer Res.

[61] Cauchi S, Ezzidi I, El Achhab Y, Mtiraoui N, Chaieb L, Salah D, Nejjari C, Labrune Y, Yengo L, Beury D, et al. European genetic variants associated with type 2 diabetes in North African Arabs. Diab Metab. 2012;38(4):316–23. 10.1016/j.diabet.2012.02.003 22463974

[62] Fontaine-Bisson B, Renstrom F, Rolandsson O, Magic, Payne F, Hallmans G, Barroso I, Franks PW. Evaluating the discriminative power of multi-trait genetic risk scores for type 2 diabetes in a northern Swedish population. Diabetologia. 2010;53(10):2155–62. 10.1007/s00125-010-1792-y PMC2931645 20571754

[63] Qian Y, Liu S, Lu F, Li H, Dong M, Lin Y, Gong J, Jin G, et al. Genetic variant in fat mass and obesity-associated gene associated with type 2 diabetes risk in Han Chinese. BMC Genet. 2013;14:86. 10.1186/1471-2156-14-86 PMC3848839 24053193

[64] Lam VK, Ma RC, Lee HM, Hu C, Park KS, Furuta H, Wang Y, Tam CH, Sim X, Ng DP, et al. Genetic associations of type 2 diabetes with islet amyloid polypeptide processing and degrading pathways in asian populations. PLoS One. 2013;8(6):e62378. 10.1371/journal.pone.0062378 PMC3679113 23776430

[65] Zhang SM, Xiao JZ, Ren Q, Han XY, Tang Y, Yang WY, Ji LN. Replication of association study between type 2 diabetes mellitus and IGF2BP2 in Han Chinese population. Chin Med J. 2013;126(21):4013–8. 24229666

[66] Yin YW, Sun QQ, Zhang BB, Hu AM, Liu HL, Wang Q, Zeng YH, Xu RJ, Zhang ZD, Zhang ZG. Association between the interleukin-6 gene −572 C/G polymorphism and the risk of type 2 diabetes mellitus: a meta-analysis of 11,681 subjects. Ann Hum Genet. 2013;77(2):106–14. 10.1111/ahg.12003 23289913

[67] Sandholt CH, Vestmar MA, Bille DS, Borglykke A, Almind K, Hansen L, Sandbaek A, Lauritzen T, Witte D, Jorgensen T, et al. Studies of metabolic phenotypic correlates of 15 obesity associated gene variants. PLoS One. 2011;6(9):e23531. 10.1371/journal.pone.0023531 PMC3166286 21912638

[68] Grarup N, Stender-Petersen KL, Andersson EA, Jorgensen T, Borch-Johnsen K, Sandbaek A, Lauritzen T, Schmitz O, Hansen T, Pedersen O. Association of variants in the sterol regulatory element-binding factor 1 (SREBF1) gene with type 2 diabetes, glycemia, and insulin resistance: a study of 15,734 Danish subjects. Diabetes. 2008;57(4):1136–42. 10.2337/db07-1534 18192539

