# Supplementary material for: Evolutionary triangulation: informing genetic association studies with evolutionary evidence
Source: BioData Min. 2016 Apr 2;9:12. doi: 10.1186/s13040-016-0091-7 (PMC4818851; doi:10.1186/s13040-016-0091-7)
Supplement: Additional file 1: Table S1. — Prevalence of phenotypes among continental populations as defined by HapMap samples. (DOCX 23 kb) [file 13040_2016_91_MOESM1_ESM.docx]

| **Phenotypes** | **Outlier** | **Common** | **Common** | **Data source** | |
| --- | --- | --- | --- | --- | --- |
| **Lactase Persistence** | CEU (80-95%) | TSI (30-80%) | CHB (0-10%) | http://milk.procon.org/sourcefiles/lactase_persistence.pdf | |
|  | CEU (80-95%) | YRI (10-30%) | GIH (30-70%) | http://milk.procon.org/sourcefiles/lactase_persistence.pdf | |
| **Melanoma/Skin Neoplasms/Carcinoma, Basal Cell/Carcinoma, Squamous Cell** | CEU (29.2 per 100,000 people) | YRI (0.48 per 100,000 people) | GIH (0.2 per 100,000 people) | http://www.cancerresearchuk.org/cancer-info/cancerstats/world/incidence/ | |
| **Diabetes Mellitus, Type 2/Insulin Resistance/Glucose Intolerance** | CEU (low) | GIH (high) | YRI (high) | http://gamapserver.who.int/gho/interactive_charts/ncd/risk_factors/blood_glucose_prevalence/atlas.html | |
|  | MEX (high) 14-20% | CEU (4-6%) | JPT (4-6%) | http://gamapserver.who.int/gho/interactive_charts/ncd/risk_factors/blood_glucose_prevalence/atlas.html | |
|  |  |  |  |  | |
| Albinism, Oculocutaneous | CEU (low) | YRI (high) | GIH (High) | http://documents.irevues.inist.fr/bitstream/handle/2042/48475/1/08-2012-OculocutaneousAlbinismID10022.pdf | |
| alpha-Thalassemia | CEU (low) | GIH (high) | YRI (high) | http://www.ojrd.com/content/5/1/13 | |
| Alzheimer Disease | CEU (6.2) | GIH (3.9) | YRI (2.6) | http://www.alz.co.uk/research/files/WorldAlzheimerReport-ExecutiveSummary.pdf | |
|  | MEX (high) | CEU | JPT | http://www.alzheimersanddementia.com/article/S1552-5260(12)02531-9/fulltext | |
| Anemia, Sickle Cell | CEU (low) | YRI (high) | GIH (high) | http://www.nhlbi.nih.gov/health/health-topics/topics/sca/atrisk.html | |
| Antiphospholipid Syndrome | CEU (54%) | GIH (27%) | YRI (27%) | http://ard.bmj.com/content/64/12/1671.full.pdf | |
| Aortic Aneurysm, Abdominal | CEU (high) | GIH (low) | YRI (low) | http://www.sciencedirect.com/science/article/pii/S0741521410013029 | |
| Arthritis, Juvenile Rheumatoid | CEU (high) | GIH (low) | YRI (low) | http://www.ncbi.nlm.nih.gov/pubmed/17530723 | |
| Arthritis, Rheumatoid | CEU (0.44%) | GIH (0.16%) | YRI (low) | http://ard.bmj.com/content/early/2014/02/18/annrheumdis-2013-204627.full.pdf+html | |
| Asthma | CEU (≥10.1%) | GIH (2.5-5.0%) | YRI(5.1-7.5%) | http://www.ginasthma.org/local/uploads/files/GINABurdenReport_1.pdf | |
| Atrial Fibrillation | CEU (8.0%) | GIH (3.9%) | YRI (3.8%) | http://www.ncbi.nlm.nih.gov/pubmed/21053705 | |
| Autistic Disorder | CEU (1/63) | GIH (1/81) | GIH (1/81) | http://www.cdc.gov/mmwr/preview/mmwrhtml/ss6302a1.htm?s_cid=ss6302a1_w | |
|  | MEX (1-4/1000) 10-40/10000 | CEU (157/10000 (UK)) | JPT 48 cases of ASD per 10,000 (aprrox 5/1000) (88.5/10000 of incedence over seven years) | http://link.springer.com/referenceworkentry/10.1007%2F978-1-4614-4788-7_162 | |
| Brain Neoplasms | CEU (5.26 per 100,000 people) | GIH (1.64 per 100,000 people) | YRI (0.39 per 100,000 people) | http://www.cancerresearchuk.org/cancer-info/cancerstats/world/incidence/ | |
| Breast Neoplasms | CEU (94.99 per 100,000 people) | GIH (25.76 per 100,000 people) | YRI (50.45 per 100,000 people) | http://www.cancerresearchuk.org/cancer-info/cancerstats/world/incidence/ | |
| Lung Neoplasms/Carcinoma, Non-Small-Cell Lung/Carcinoma, Small Cell | CEU (29.95 per 100,000 people) | GIH (6.92 per 100,000 people) | YRI (1.07 per 100,000 people) | http://www.cancerresearchuk.org/cancer-info/cancerstats/world/incidence/ | |
| Carcinoma, Renal Cell/Kidney Neoplasms | CEU (8.21 per 100,000 people) | GIH (0.91 per 100,000 people) | YRI (0.64 per 100,000 people) | http://www.cancerresearchuk.org/cancer-info/cancerstats/world/incidence/ | |
| Carotid Stenosis | CEU (high) | GIH (low) | YRI (low) | http://www.ncbi.nlm.nih.gov/pubmed/23177534 | |
| Cataract | CEU (high) | GIH (low) | YRI (low) | https://www.nei.nih.gov/eyedata/cataract.asp | |
| Brain Ischemia/Stroke | European (low) | GIH (high) | YRI (high) | http://www.nhs.uk/Conditions/Stroke/Pages/Whosatriskpage.aspx | |
| Brucellosis | CEU (<2 per 1,000,000 people) | GIH (possibly endemic, no data) | YRI (possibly endemic, no data) | http://www.sciencedirect.com/science/article/pii/S1473309906703826 | |
| Colitis, Ulcerative/Crohn disease/Inflammatory Bowel Diseases | CEU (high) | GIH (low) | YRI (low) | http://www.ncbi.nlm.nih.gov/pubmed/23518811 | |
| Colonic Neoplasms/Colorectal Neoplasms/Rectal Neoplasms | CEU (30.16 per 100,000 people) | GIH (6.09 per 100,000 people) | YRI (4.21 per 100,000 people) | http://www.cancerresearchuk.org/cancer-info/cancerstats/world/incidence/ | |
| Diabetes Mellitus, Type 1 | CEU (high) | GIH (low) | YRI (low) | http://gamapserver.who.int/gho/interactive_charts/ncd/risk_factors/blood_glucose_prevalence/atlas.html | |
| Diabetic Nephropathies | CEU (high) | GIH (low) | YRI (low) | http://care.diabetesjournals.org/content/26/8/2392/T3.expansion.html | |
| Celiac disease | CEU (high) | GIH (low) | YRI (low) | http://www.ncbi.nlm.nih.gov/pubmed/22850429 | |
| Endometrial Neoplasms | CEU (high) | GIH (low) | YRI (low) | http://aje.oxfordjournals.org/content/165/3/262.long | |
| Epstein-Barr Virus Infections | White (26%) | Blacks (74%) | Asians (62%) | http://cid.oxfordjournals.org/content/early/2014/05/11/cid.ciu342.full.pdf+html | |
| Glioma/Brain Neoplasms | CEU (5.26 per 100,000 people) | GIH (1.64 per 100,000 people) | YRI (0.39 per 100,000 people) | http://www.cancerresearchuk.org/cancer-info/cancerstats/world/incidence/ | |
| Glucose 6 phosphate Dehydrogenase Deficiency | CEU (low) | GIH (high) | YRI (high) | http://www.hopkinsmedicine.org/healthlibrary/conditions/hematology_and_blood_disorders/g6pd_glucose-6-phosphate_dehydrogenase_deficiency_85,P00091/ | |
| Graves’ Disease | CEU (low) | GIH (high) | YRI (high) | http://www.ncbi.nlm.nih.gov/pubmed/24737370 | |
| Hepatitis B | CEU (low) | GIH (high) | YRI (high) | http://www.who.int/csr/disease/hepatitis/whocdscsrlyo20022/en/index1.html | |
| Hodgkin Disease (multiple names) | CEU (2.34 per 100,000 people) | GIH (0.7 per 100,000 people) | YRI (0.67 per 100,000 people) | http://www.cancerresearchuk.org/cancer-info/cancerstats/world/incidence/ | |
| Hypertrophy, Left Ventricular | CEU (low) | GIH (high) | YRI (high) | http://www.nature.com/jhh/journal/v27/n5/full/jhh201250a.html | |
| Kidney Diseases/Kidney Failure, Chronic/Renal Insufficiency, Chronic | CEU (low) | GIH (high) | YRI (high) | http://www.nhs.uk/Livewell/Kidneyhealth/Pages/BlackandAsiankidneyhealth.aspx | |
| Leukemia/Leukemia, Lymphocytic, Acute/Leukemia, Myeloid | CEU (7.47 per 100,000 people) | GIH (2.79 per 100,000 people) | YRI (1.5 per 100,000 people) | http://www.cancerresearchuk.org/cancer-info/cancerstats/world/incidence/ | |
| Lupus Erythematosus, Systemic | CEU (4.3 per 100,000 people) | GIH (20.7 per 100,000 people) | YRI (25.8 per 100,000 people) | http://onlinelibrary.wiley.com/doi/10.1002/art.1780380415/pdf | |
| Lymphoma, Non-Hodgkin | CEU (10.1 per 100,000 people) | GIH (2.19 per 100,000 people) | YRI (3.24 per 100,000 people) | | http://www.cancerresearchuk.org/cancer-info/cancerstats/world/incidence/ |
| Malaria/Malaria, Falciparum | CEU (low) | GIH (high) | YRI (high) | http://www.who.int/gho/malaria/en/ | |
| Multiple Sclerosis | CEU (0.164%) | GIH (0.007%) | YRI (0.001%) | http://www.msif.org/about-us/advocacy/atlas/ (estimated based on 'Epidemiology of MS' and 'number of people with MS' in respective regions Search 2013 data) | |
| Obstetric Labor, Premature/Premature Birth | CEU (<10 per 100 live births) | GIH (10-15 per 100 live births) | YRI (10-15 per 100 live births) | http://www.marchofdimes.com/mission/global-preterm.aspx | |
| Opioid-Related Disorders | CEU (1.2%) | GIH (0.3) | YRI (0.6%) | http://www.ncbi.nlm.nih.gov/pubmed/22065533 | |
| Ovarian Neoplasms | CEU (11.74 per 100,000 people) | GIH (4.86 per 100,000 people) | YRI (3.06 per 100,000 people) | http://www.cancerresearchuk.org/cancer-info/cancerstats/world/incidence/ | |
| Pain Threshold | CEU (high) | GIH (low) | YRI (low) | http://link.springer.com/chapter/10.1007/978-1-60327-465-4_17 | |
| Pancreatic Neoplasms | CEU (6.27 per 100,000 people) | GIH (1.16 per 100,000 people) | YRI (1.83 per 100,000 people) | http://www.cancerresearchuk.org/cancer-info/cancerstats/world/incidence/ | |
| Panic Disorder | CEU (OR vs. YRI 1.701, OR vs. GIH 2.475) | GIH | YRI | http://onlinelibrary.wiley.com/doi/10.1111/j.1755-5949.2009.00092.x/pdf | |
| Parkinson Disease | CEU (high) | GIH (OR vs. CEU 0.62) | YRI (OR vs. CEU 0.58) | http://www.ncbi.nlm.nih.gov/pmc/articles/PMC2865395/ | |
| Prostatic Neoplasms | CEU (107.0 per 100,000 people) | GIH (4.16 per 100,000 people) | YRI (19.8 per 100,000 people) | http://www.cancerresearchuk.org/cancer-info/cancerstats/world/incidence/ | |
| Pulmonary Disease, Chronic Obstructive | CEU (high) | GIH (low) | YRI (low) | http://www.cdc.gov/copd/ | |
| Smith-Lemli-Opitz Syndrome | CEU (high) | GIH (low) | YRI (low) | http://www.ncbi.nlm.nih.gov/pubmed/11503168 | |
| Tuberculosis/Tuberculosis, Pulmonary | CEU (low) | GIH (high) | YRI (high) | http://www.who.int/mediacentre/factsheets/fs104/en/ | |
| Urinary Bladder Neoplasms | CEU (5.75 per 100,000 people) | GIH (1.61 per 100,000 people) | YRI (1.04 per 100,000 people) | http://www.cancerresearchuk.org/cancer-info/cancerstats/world/incidence/ | |
| Uterine Cervical Neoplasms/Uterine Neoplasms | CEU (7.14 per 100,000 people) | GIH (21.99 per 100,000 people) | YRI (28.98 per 100,000 people) | http://www.cancerresearchuk.org/cancer-info/cancerstats/world/incidence/ | |
| Vitamin D Deficiency | CEU (6%) | GIH (44%) | YRI (12.5%) | http://www.ncbi.nlm.nih.gov/pubmed/23140614 | |

Phenotypes in bold are index phenotypes.
