# Supplementary material for: Evolutionary triangulation: informing genetic association studies with evolutionary evidence
Source: BioData Min. 2016 Apr 2;9:12. doi: 10.1186/s13040-016-0091-7 (PMC4818851; doi:10.1186/s13040-016-0091-7)
Supplement: Additional file 3: Table S3a. — SNPs and genes identified using the 95th/5th percentile ET cutoffs among CEU-TSI-CHB. Table S3b. SNPs and genes identified using the 90th/10th percentile ET cutoffs among CEU-TSI-CHB. Table S3c. SNPs and genes identified using the 85th/15th percentile ET cutoffs among CEU-TSI-CHB. Table S3d. SNPs and genes identified using the 80th/20th percentile ET cutoffs among CEU-TSI-CHB. (DOCX 26 kb) [file 13040_2016_91_MOESM3_ESM.docx]

**Table S3a. SNPs and genes identified using the 95^th^/5^th^ percentile ET cutoffs among CEU-TSI-CHB**

| Marker | Chromosome | Base pair position (Build 37) | Genes within 100 Kb |
| --- | --- | --- | --- |
| rs6739713 | 2 | 136488978 | *R3HDM1,MIR128-1,UBXN4,LCT,LOC100507600* |
| rs10202489 | 2 | 136494186 | *R3HDM1,MIR128-1,UBXN4,LCT,LOC100507600* |
| rs1438307 | 2 | 136499166 | *R3HDM1,MIR128-1,UBXN4,LCT,LOC100507600,MCM6* |
| rs3213889 | 2 | 136511575 | *R3HDM1,MIR128-1,UBXN4,LCT,LOC100507600,MCM6* |
| rs9287442 | 2 | 136522710 | *R3HDM1,MIR128-1,UBXN4,LCT,LOC100507600,MCM6* |
| rs2322660 | 2 | 136557319 | *R3HDM1,UBXN4,LCT,LOC100507600,MCM6* |
| rs2236783 | 2 | 136594158 | *UBXN4,LCT,LOC100507600,MCM6,DARS* |
| rs309137 | 2 | 136765951 | *DARS,LOC101928243* |

**Table S3b. SNPs and genes identified using the 90^th^/10^th^ percentile ET cutoffs among CEU-TSI-CHB**

| Marker | Chromosome | Base pair position (Build 37) | Genes within 100 Kb |
| --- | --- | --- | --- |
| rs16831243 | 2 | 135762344 | *CCNT2-AS1,CCNT2,MAP3K19,RAB3GAP1* |
| rs7606415 | 2 | 135762980 | *CCNT2-AS1,CCNT2,MAP3K19,RAB3GAP1* |
| rs13390171 | 2 | 135777504 | *CCNT2,MAP3K19,RAB3GAP1* |
| rs10445686 | 2 | 135893372 | *RAB3GAP1,ZRANB3* |
| rs2278731 | 2 | 136396300 | *R3HDM1,MIR128-1* |
| rs1446586 | 2 | 136407445 | *R3HDM1,MIR128-1,UBXN4* |
| rs6753232 | 2 | 136436317 | *R3HDM1,MIR128-1,UBXN4* |
| rs6739713 | 2 | 136488978 | *R3HDM1,MIR128-1,UBXN4,LCT,LOC100507600* |
| rs10202489 | 2 | 136494186 | *R3HDM1,MIR128-1,UBXN4,LCT,LOC100507600* |
| rs1438307 | 2 | 136499166 | *R3HDM1,MIR128-1,UBXN4,LCT,LOC100507600,MCM6* |
| rs3213889 | 2 | 136511575 | *R3HDM1,MIR128-1,UBXN4,LCT,LOC100507600,MCM6* |
| rs9287442 | 2 | 136522710 | *R3HDM1,MIR128-1,UBXN4,LCT,LOC100507600,MCM6* |
| rs2278544 | 2 | 136546110 | *R3HDM1,UBXN4,LCT,LOC100507600,MCM6* |
| rs2322659 | 2 | 136555659 | *R3HDM1,UBXN4,LCT,LOC100507600,MCM6* |
| rs2322660 | 2 | 136557319 | *R3HDM1,UBXN4,LCT,LOC100507600,MCM6* |
| rs9636213 | 2 | 136589278 | *UBXN4,LCT,LOC100507600,MCM6,DARS* |
| rs3754689 | 2 | 136590746 | *UBXN4,LCT,LOC100507600,MCM6,DARS* |
| rs2236783 | 2 | 136594158 | *UBXN4,LCT,LOC100507600,MCM6,DARS* |
| rs309152 | 2 | 136657252 | *LCT,LOC100507600,MCM6,DARS,LOC101928243* |
| rs309165 | 2 | 136667301 | *LCT,LOC100507600,MCM6,DARS,LOC101928243* |
| rs309170 | 2 | 136672775 | *LCT,LOC100507600,MCM6,DARS,LOC101928243* |
| rs309172 | 2 | 136674028 | *LCT,LOC100507600,MCM6,DARS,LOC101928243* |
| rs309158 | 2 | 136680534 | *LCT,LOC100507600,MCM6,DARS,LOC101928243* |
| rs309161 | 2 | 136688749 | *LCT,MCM6,DARS,LOC101928243* |
| rs3112496 | 2 | 136711064 | *MCM6,DARS,LOC101928243* |
| rs309142 | 2 | 136715324 | *MCM6,DARS,LOC101928243* |
| rs660002 | 2 | 136723504 | *MCM6,DARS,LOC101928243* |
| rs309137 | 2 | 136765951 | *DARS,LOC101928243* |
| rs2176717 | 2 | 136899859 | *CXCR4* |

**Table S3c. SNPs and genes identified using the 85^th^/15^th^ percentile ET cutoffs among CEU-TSI-CHB**

| Marker | Chromosome | Base pair position (Build 37) | Genes within 100 Kb |
| --- | --- | --- | --- |
| rs16831243 | 2 | 135762344 | *CCNT2-AS1,CCNT2,MAP3K19,RAB3GAP1* |
| rs7606415 | 2 | 135762980 | *CCNT2-AS1,CCNT2,MAP3K19,RAB3GAP1* |
| rs13390171 | 2 | 135777504 | *CCNT2,MAP3K19,RAB3GAP1* |
| rs10445686 | 2 | 135893372 | *RAB3GAP1,ZRANB3* |
| rs2278731 | 2 | 136396300 | *R3HDM1,MIR128-1* |
| rs1446586 | 2 | 136407445 | *R3HDM1,MIR128-1,UBXN4* |
| rs6753232 | 2 | 136436317 | *R3HDM1,MIR128-1,UBXN4* |
| rs7592050 | 2 | 136459227 | *R3HDM1,MIR128-1,UBXN4,LCT* |
| rs2304366 | 2 | 136467119 | *R3HDM1,MIR128-1,UBXN4,LCT* |
| rs6739713 | 2 | 136488978 | *R3HDM1,MIR128-1,UBXN4,LCT,LOC100507600* |
| rs10202489 | 2 | 136494186 | *R3HDM1,MIR128-1,UBXN4,LCT,LOC100507600* |
| rs1438307 | 2 | 136499166 | *R3HDM1,MIR128-1,UBXN4,LCT,LOC100507600,MCM6* |
| rs3213889 | 2 | 136511575 | *R3HDM1,MIR128-1,UBXN4,LCT,LOC100507600,MCM6* |
| rs9287442 | 2 | 136522710 | *R3HDM1,MIR128-1,UBXN4,LCT,LOC100507600,MCM6* |
| rs2278544 | 2 | 136546110 | *R3HDM1,UBXN4,LCT,LOC100507600,MCM6* |
| rs2322659 | 2 | 136555659 | *R3HDM1,UBXN4,LCT,LOC100507600,MCM6* |
| rs9636213 | 2 | 136589278 | *UBXN4,LCT,LOC100507600,MCM6,DARS* |
| rs3754689 | 2 | 136590746 | *UBXN4,LCT,LOC100507600,MCM6,DARS* |
| rs309152 | 2 | 136657252 | *LCT,LOC100507600,MCM6,DARS,LOC101928243* |
| rs309165 | 2 | 136667301 | *LCT,LOC100507600,MCM6,DARS,LOC101928243* |
| rs309170 | 2 | 136672775 | *LCT,LOC100507600,MCM6,DARS,LOC101928243* |
| rs309172 | 2 | 136674028 | *LCT,LOC100507600,MCM6,DARS,LOC101928243* |
| rs309158 | 2 | 136680534 | *LCT,LOC100507600,MCM6,DARS,LOC101928243* |
| rs309161 | 2 | 136688749 | *LCT,MCM6,DARS,LOC101928243* |
| rs3112496 | 2 | 136711064 | *MCM6,DARS,LOC101928243* |
| rs309142 | 2 | 136715324 | *MCM6,DARS,LOC101928243* |
| rs660002 | 2 | 136723504 | *MCM6,DARS,LOC101928243* |
| rs309137 | 2 | 136765951 | *DARS,LOC101928243* |
| rs2176717 | 2 | 136899859 | *CXCR4* |
| rs11692725 | 2 | 136929883 | *CXCR4* |
| rs12691876 | 2 | 136963794 | *CXCR4* |
| rs882300 | 2 | 136976255 |  |
| rs3951770 | 2 | 137544376 |  |

**Table S3d. SNPs and genes identified using the 80^th^/20^th^ percentile ET cutoffs among CEU-TSI-CHB**

| Marker | Chromosome | Base pair Position (Build 37) | Genes within 100 Kb |
| --- | --- | --- | --- |
| rs655472 | 2 | 135290221 | *MGAT5,TMEM163* |
| rs666614 | 2 | 135290453 | *MGAT5,TMEM163* |
| rs1568121 | 2 | 135406606 | *TMEM163* |
| rs16831243 | 2 | 135762344 | *CCNT2-AS1,CCNT2,MAP3K19,RAB3GAP1* |
| rs10174462 | 2 | 135762429 | *CCNT2-AS1,CCNT2,MAP3K19,RAB3GAP1* |
| rs10197646 | 2 | 135762656 | *CCNT2-AS1,CCNT2,MAP3K19,RAB3GAP1* |
| rs7606415 | 2 | 135762980 | *CCNT2-AS1,CCNT2,MAP3K19,RAB3GAP1* |
| rs7582173 | 2 | 135763318 | *CCNT2-AS1,CCNT2,MAP3K19,RAB3GAP1* |
| rs13390171 | 2 | 135777504 | *CCNT2,MAP3K19,RAB3GAP1* |
| rs10445686 | 2 | 135893372 | *RAB3GAP1,ZRANB3* |
| rs961360 | 2 | 136393658 | *R3HDM1,MIR128-1* |
| rs2278731 | 2 | 136396300 | *R3HDM1,MIR128-1* |
| rs1446586 | 2 | 136407445 | *R3HDM1,MIR128-1,UBXN4* |
| rs6753232 | 2 | 136436317 | *R3HDM1,MIR128-1,UBXN4* |
| rs7592050 | 2 | 136459227 | *R3HDM1,MIR128-1,UBXN4,LCT* |
| rs2304366 | 2 | 136467119 | *R3HDM1,MIR128-1,UBXN4,LCT* |
| rs6739713 | 2 | 136488978 | *R3HDM1,MIR128-1,UBXN4,LCT,LOC100507600* |
| rs10202489 | 2 | 136494186 | *R3HDM1,MIR128-1,UBXN4,LCT,LOC100507600* |
| rs1438307 | 2 | 136499166 | *R3HDM1,MIR128-1,UBXN4,LCT,LOC100507600,MCM6* |
| rs3213889 | 2 | 136511575 | *R3HDM1,MIR128-1,UBXN4,LCT,LOC100507600,MCM6* |
| rs9287442 | 2 | 136522710 | *R3HDM1,MIR128-1,UBXN4,LCT,LOC100507600,MCM6* |
| rs2278544 | 2 | 136546110 | *R3HDM1,UBXN4,LCT,LOC100507600,MCM6* |
| rs2322659 | 2 | 136555659 | *R3HDM1,UBXN4,LCT,LOC100507600,MCM6* |
| rs2322660 | 2 | 136557319 | *R3HDM1,UBXN4,LCT,LOC100507600,MCM6* |
| rs9636213 | 2 | 136589278 | *UBXN4,LCT,LOC100507600,MCM6,DARS* |
| rs3754689 | 2 | 136590746 | *UBXN4,LCT,LOC100507600,MCM6,DARS* |
| rs2236783 | 2 | 136594158 | *UBXN4,LCT,LOC100507600,MCM6,DARS* |
| rs3754686 | 2 | 136603276 | *UBXN4,LCT,LOC100507600,MCM6,DARS* |
| rs309152 | 2 | 136657252 | *LCT,LOC100507600,MCM6,DARS,LOC101928243* |
| rs309165 | 2 | 136667301 | *LCT,LOC100507600,MCM6,DARS,LOC101928243* |
| rs309170 | 2 | 136672775 | *LCT,LOC100507600,MCM6,DARS,LOC101928243* |
| rs309172 | 2 | 136674028 | *LCT,LOC100507600,MCM6,DARS,LOC101928243* |
| rs309158 | 2 | 136680534 | *LCT,LOC100507600,MCM6,DARS,LOC101928243* |
| rs309161 | 2 | 136688749 | *LCT,MCM6,DARS,LOC101928243* |
| rs3112496 | 2 | 136711064 | *MCM6,DARS,LOC101928243* |
| rs309142 | 2 | 136715324 | *MCM6,DARS,LOC101928243* |
| rs660002 | 2 | 136723504 | *MCM6,DARS,LOC101928243* |
| rs309137 | 2 | 136765951 | *DARS,LOC101928243* |
| rs2176717 | 2 | 136899859 | *CXCR4* |
| rs11692725 | 2 | 136929883 | *CXCR4* |
| rs12691876 | 2 | 136963794 | *CXCR4* |
| rs1519528 | 2 | 136973781 | *CXCR4* |
| rs882300 | 2 | 136976255 |  |
| rs10204606 | 2 | 137165497 |  |
| rs12691887 | 2 | 137173367 |  |
| rs3951770 | 2 | 137544376 |  |
| rs382259 | 6 | 32209027 | *PRRT1,LOC100507547,PPT2,PPT2-EGFL8,EGFL8,AGPAT1,MIR6721,RNF5,RNF5P1,MIR6833,AGER,PBX2,GPSM3,NOTCH4,C6orf10* |
| rs419132 | 6 | 32210799 | *PRRT1,LOC100507547,PPT2,PPT2-EGFL8,EGFL8,AGPAT1,MIR6721,RNF5,RNF5P1,MIR6833,AGER,PBX2,GPSM3,NOTCH4,C6orf10* |
| rs427037 | 6 | 32212264 | *PRRT1,LOC100507547,PPT2,PPT2-EGFL8,EGFL8,AGPAT1,MIR6721,RNF5,RNF5P1,MIR6833,AGER,PBX2,GPSM3,NOTCH4,C6orf10* |
| rs5966453 | 23 | 144272488 | *SPANXN1* |
