# Supplementary material for: Evolutionary triangulation: informing genetic association studies with evolutionary evidence
Source: BioData Min. 2016 Apr 2;9:12. doi: 10.1186/s13040-016-0091-7 (PMC4818851; doi:10.1186/s13040-016-0091-7)
Supplement: Additional file 4: Figure S1. — Distribution of recombination hotspots surrounding ET SNP rs12615624, idenitifed by comparing CEU-TSI (high Fst; 95th percentile), CEU-CHB (high Fst; 95th percentile) and TSICHB (low Fst; 5th percentile). (PDF 15 kb) [file 13040_2016_91_MOESM4_ESM.pdf]

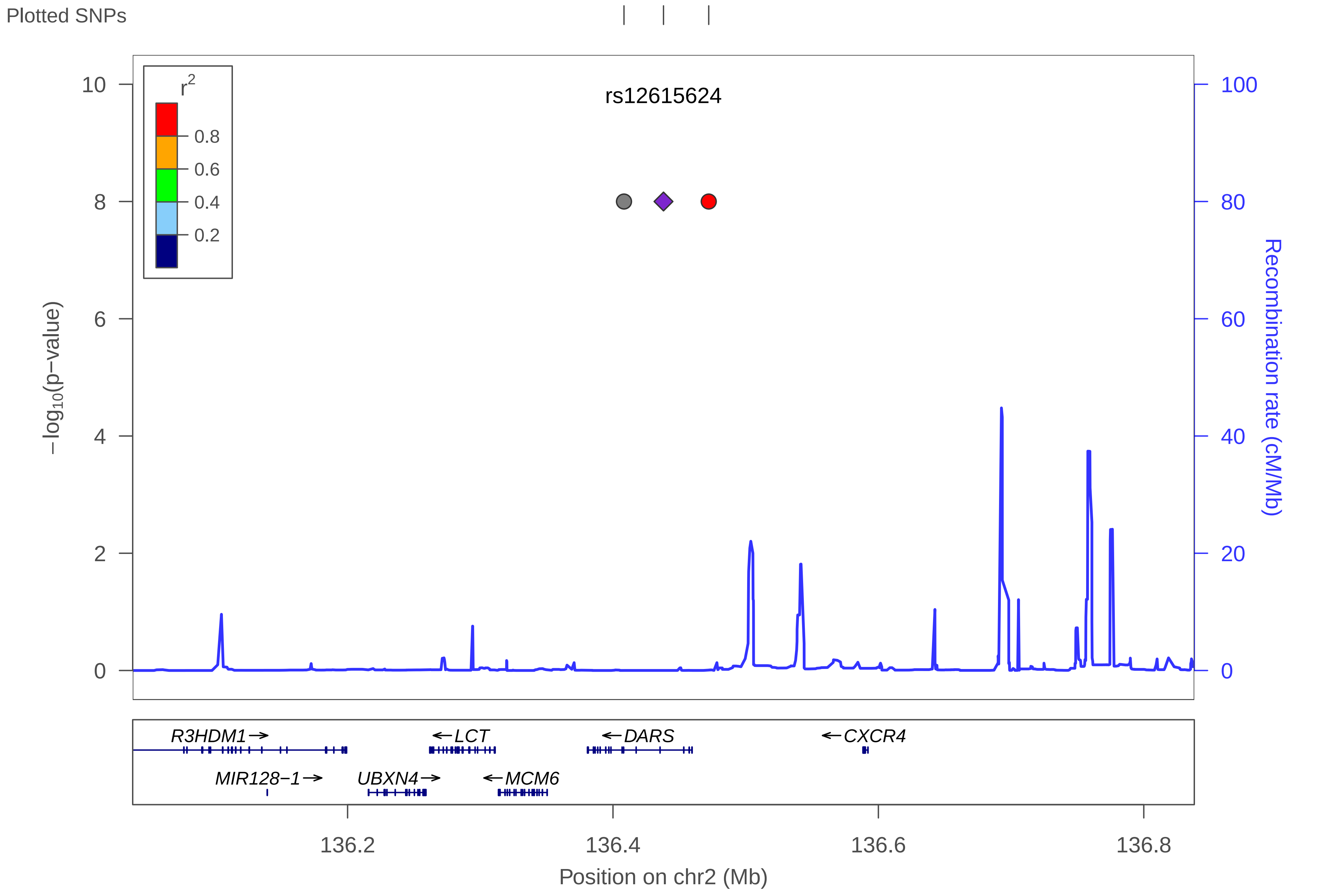

Plotted SNPs
rs12615624
r2
0.8
0.6
0.4
0.2
−log10(p−value)
Recombination rate (cM/Mb)
R3HDM1→
←LCT
←DARS
←CXCR4
MIR128−1→
UBXN4→
←MCM6
136.2
136.4
136.6
136.8
Position on chr2 (Mb)

date:	Sun Dec 6 18:23:23 2015
build:	hg18
display range:	chr2:136038073−136838073 [136038073−136838073]
hilite range:	0 − 0 [0 − 0]
reference SNP:	chr2:136438073
number of SNPs plotted:	3
max P−Value:	1E−8 [chr2:136408295]
min P−Value:	1E−8 [chr2:136408295]

Make more plots at http://csg.sph.umich.edu/locuszoom/
