# Supplementary material for: Evolutionary triangulation: informing genetic association studies with evolutionary evidence
Source: BioData Min. 2016 Apr 2;9:12. doi: 10.1186/s13040-016-0091-7 (PMC4818851; doi:10.1186/s13040-016-0091-7)
Supplement: Additional file 5: Table S4. — FST values at each of the percentile thresholds. (DOCX 14 kb) [file 13040_2016_91_MOESM5_ESM.docx]

**Table S4. F_ST_ values at each of the percentile thresholds**

1. **CEU-TSI-CHB (lactose intolerance)**

|  | 95th/5th | 90th/10th | 85th/15th | 80th/20th |
| --- | --- | --- | --- | --- |
| Fst(CEU_TSI) | 0.0276 | 0.018 | 0.0125 | 0.009 |
| Fst(CEU_CHB) | 0.3298 | 0.247 | 0.1976 | 0.1611 |
| Fst(TSI_CHB) | 0 | 0 | 0 | 0.0017 |

1. **CEU-GIH-YRI (melanoma)**

|  | 95th/5th | 90th/10th | 85th/15th |
| --- | --- | --- | --- |
| Fst(CEU_GIH) | 0.1294 | 0.0922 | 0.0708 |
| Fst(CEU_YRI) | 0.4317 | 0.3335 | 0.2706 |
| Fst(GIH_YRI) | 0 | 0 | 7.65E-04 |

1. **MEX-JPT-CEU (Type 2 diabetes mellitus)**

|  | 95th/5th | 90th/10th | 85th/15th |
| --- | --- | --- | --- |
| Fst(MEX_JPT) | 0.245 | 0.1776 | 0.1385 |
| Fst(MEX_CEU) | 0.1314 | 0.0884 | 0.0654 |
| Fst(JPT_CEU) | 0 | 0 | 0 |
