# Supplementary material for: Evolutionary triangulation: informing genetic association studies with evolutionary evidence
Source: BioData Min. 2016 Apr 2;9:12. doi: 10.1186/s13040-016-0091-7 (PMC4818851; doi:10.1186/s13040-016-0091-7)
Supplement: Additional file 6: Table S5a. — SNPs and genes identified using the 95th/5th percentile ET cutoffs among CEU-GIH-YRI. Table S5b. SNPs and genes identified using the 90th/10th percentile ET cutoffs among CEU-GIH-YRI. Table S5c. SNPs and genes identified using the 85th/15th percentile ET cutoffs among CEU-GIH-YRI. (DOCX 96 kb) [file 13040_2016_91_MOESM6_ESM.docx]

**Table S5a. SNPs and genes identified using the 95^th^/5^th^ percentile ET cutoffs among CEU-GIH-YRI**

| Marker | Chromosome | Base pair position (Build 37) | Genes within 100 Kb |
| --- | --- | --- | --- |
| rs309164 | 2 | 136691825 | *LCT,MCM6,DARS,LOC101928243* |
| rs12615624 | 2 | 136721603 | *MCM6,DARS,LOC101928243* |
| rs309134 | 2 | 136755684 | *DARS,LOC101928243* |
| rs28117 | 5 | 33962770 | *ADAMTS12,RXFP3,SLC45A2,AMACR,C1QTNF3-AMACR,C1QTNF3* |
| rs10511625 | 9 | 16693969 | *BNC2* |
| rs10511624 | 9 | 16695690 | *BNC2* |
| rs1888207 | 9 | 16696187 | *BNC2* |
| rs10738446 | 9 | 16696510 | *BNC2* |
| rs10962542 | 9 | 16698234 | *BNC2* |
| rs10810593 | 9 | 16699008 | *BNC2* |
| rs2297176 | 9 | 16706012 | *BNC2* |
| rs12791871 | 11 | 71164544 | *FLJ42102,DHCR7,NADSYN1,MIR6754,KRTAP5-7,KRTAP5-8,KRTAP5-9* |
| rs7944926 | 11 | 71165625 | *FLJ42102,DHCR7,NADSYN1,MIR6754,KRTAP5-7,KRTAP5-8,KRTAP5-9* |
| rs12785878 | 11 | 71167449 | *FLJ42102,DHCR7,NADSYN1,MIR6754,KRTAP5-7,KRTAP5-8,KRTAP5-9* |
| rs2707160 | 23 | 23923684 | *APOO,CXorf58,KLHL15* |
| rs3813165 | 23 | 24005086 | *APOO,CXorf58,KLHL15,EIF2S3* |
| rs5970824 | 23 | 24041718 | *CXorf58,KLHL15,EIF2S3* |
| rs11094949 | 23 | 24104646 | *KLHL15,EIF2S3,ZFX-AS1,ZFX* |
| rs5986763 | 23 | 25261728 |  |
| rs5986769 | 23 | 25276181 |  |
| rs2079521 | 23 | 30320215 | *MAGEB2,MAGEB3,MAGEB4,MAGEB1,NR0B1* |
| rs3012658 | 23 | 71566745 | *PIN4,RPS4X,CITED1,HDAC8* |

**Table S5b. SNPs and genes identified using the 90^th^/10^th^ percentile ET cutoffs among CEU-GIH-YRI**

| Marker | Chromosome | Base pair position (Build 37) | Genes within 100 Kb |
| --- | --- | --- | --- |
| rs1555745 | 1 | 39483129 | *RHBDL2,AKIRIN1,NDUFS5,MACF1* |
| rs1555741 | 1 | 39493121 | *RHBDL2,AKIRIN1,NDUFS5,MACF1* |
| rs7554809 | 1 | 39503878 | *RHBDL2,AKIRIN1,NDUFS5,MACF1* |
| rs607945 | 1 | 47212694 | *ATPAF1,TEX38,EFCAB14-AS1,EFCAB14,CYP4B1,CYP4Z2P* |
| rs594387 | 1 | 47213386 | *ATPAF1,TEX38,EFCAB14-AS1,EFCAB14,CYP4B1,CYP4Z2P* |
| rs12738201 | 1 | 75275749 | *CRYZ,TYW3* |
| rs35263946 | 1 | 116064403 |  |
| rs2153904 | 1 | 205642790 | *MFSD4,ELK4,SLC45A3,NUCKS1,RAB7L1* |
| rs1481356 | 1 | 218908668 |  |
| rs1524007 | 2 | 13699487 |  |
| rs7422512 | 2 | 18439946 |  |
| rs6707939 | 2 | 28487706 | *BRE,LOC100505716* |
| rs2005181 | 2 | 28549757 | *BRE,LOC100505716,FLJ31356,FOSL2* |
| rs6732543 | 2 | 28562703 | *BRE,LOC100505716,FLJ31356,FOSL2* |
| rs539588 | 2 | 135212329 | *MGAT5,TMEM163* |
| rs544050 | 2 | 135212819 | *MGAT5,TMEM163* |
| rs2053726 | 2 | 135610154 | *ACMSD,CCNT2-AS1,CCNT2* |
| rs12469941 | 2 | 135629927 | *ACMSD,CCNT2-AS1,CCNT2,MAP3K19* |
| rs4954193 | 2 | 135642180 | *ACMSD,CCNT2-AS1,CCNT2,MAP3K19* |
| rs12469411 | 2 | 135664556 | *ACMSD,CCNT2-AS1,CCNT2,MAP3K19* |
| rs1374289 | 2 | 135694379 | *ACMSD,CCNT2-AS1,CCNT2,MAP3K19* |
| rs1530559 | 2 | 135755629 | *ACMSD,CCNT2-AS1,CCNT2,MAP3K19,RAB3GAP1* |
| rs309164 | 2 | 136691825 | *LCT,MCM6,DARS,LOC101928243* |
| rs12615624 | 2 | 136721603 | *MCM6,DARS,LOC101928243* |
| rs309134 | 2 | 136755684 | *DARS,LOC101928243* |
| rs13392503 | 2 | 137431134 |  |
| rs6433083 | 2 | 169499314 | *CERS6,MIR4774* |
| rs7591406 | 2 | 169499335 | *CERS6,MIR4774* |
| rs1834271 | 2 | 169506219 | *CERS6,MIR4774* |
| rs2293554 | 2 | 202131587 | *CFLAR,CASP10,CASP8,ALS2CR12* |
| rs1682914 | 3 | 3844751 | *LRRN1* |
| rs4073664 | 3 | 4682456 | *ITPR1* |
| rs520418 | 3 | 172138843 | *FNDC3B,GHSR,TNFSF10* |
| rs9864104 | 3 | 185357531 | *LIPH,SENP2,IGF2BP2,C3orf65* |
| rs11133274 | 4 | 54299380 | *SCFD2,FIP1L1,LNX1,LNX1-AS1* |
| rs6810557 | 4 | 54351687 | *FIP1L1,LNX1,LNX1-AS1* |
| rs6554109 | 4 | 54352035 | *FIP1L1,LNX1,LNX1-AS1* |
| rs6827408 | 4 | 61177831 |  |
| rs2604209 | 4 | 89621072 | *HERC3,NAP1L5,FAM13A-AS1,FAM13A* |
| rs2914255 | 5 | 23465827 | *PRDM9* |
| rs28117 | 5 | 33962770 | *ADAMTS12,RXFP3,SLC45A2,AMACR,C1QTNF3-AMACR,C1QTNF3* |
| rs4429894 | 5 | 109499203 |  |
| rs7748599 | 6 | 38363213 | *BTBD9* |
| rs4711546 | 6 | 38366186 | *BTBD9* |
| rs7771618 | 6 | 111131329 | *CDK19,AMD1* |
| rs1474347 | 7 | 22768124 | *IL6,TOMM7* |
| rs799618 | 7 | 110485378 | *IMMP2L* |
| rs12673425 | 7 | 113541607 | *PPP1R3A* |
| rs6974649 | 7 | 130813097 | *LINC-PINT,MKLN1* |
| rs6947916 | 7 | 130815342 | *LINC-PINT,MKLN1* |
| rs17751178 | 8 | 10116730 | *MSRA* |
| rs10504155 | 8 | 54429080 |  |
| rs1483538 | 8 | 54766847 | *ATP6V1H,RGS20* |
| rs7827611 | 8 | 54788251 | *ATP6V1H,RGS20,TCEA1* |
| rs1384797 | 8 | 54793928 | *ATP6V1H,RGS20,TCEA1* |
| rs6980924 | 8 | 62156050 | *CLVS1* |
| rs9643526 | 8 | 62167697 | *CLVS1* |
| rs3852344 | 8 | 62207427 | *CLVS1* |
| rs4301437 | 8 | 125460073 | *TMEM65,TRMT12,RNF139-AS1,RNF139,TATDN1,MIR6844,NDUFB9* |
| rs10810590 | 9 | 16689291 | *BNC2* |
| rs10511625 | 9 | 16693969 | *BNC2* |
| rs10511624 | 9 | 16695690 | *BNC2* |
| rs1888207 | 9 | 16696187 | *BNC2* |
| rs10738446 | 9 | 16696510 | *BNC2* |
| rs10962542 | 9 | 16698234 | *BNC2* |
| rs10810593 | 9 | 16699008 | *BNC2* |
| rs2297176 | 9 | 16706012 | *BNC2* |
| rs10810611 | 9 | 16756377 | *BNC2* |
| rs220366 | 10 | 24592764 | *KIAA1217,MIR603* |
| rs11193407 | 10 | 109161645 |  |
| rs10787024 | 10 | 109171971 |  |
| rs972353 | 11 | 61717715 | *FADS2,FADS3,MIR6746,RAB3IL1,BEST1,FTH1* |
| rs2736597 | 11 | 61718036 | *FADS2,FADS3,MIR6746,RAB3IL1,BEST1,FTH1* |
| rs1800007 | 11 | 61719387 | *FADS2,FADS3,MIR6746,RAB3IL1,BEST1,FTH1* |
| rs586616 | 11 | 64707978 | *CDC42BPG,EHD1,MIR192,MIR194-2,ATG2A,MIR6750,MIR6749,PPP2R5B,GPHA2,C11orf85,BATF2,ARL2-SNX15,ARL2,MIR6879,SNX15* |
| rs596321 | 11 | 64713485 | *EHD1,MIR192,MIR194-2,ATG2A,MIR6750,MIR6749,PPP2R5B,GPHA2,C11orf85,BATF2,ARL2-SNX15,ARL2,MIR6879,SNX15,SAC3D1,NAALADL1* |
| rs602404 | 11 | 64726701 | *EHD1,MIR192,MIR194-2,ATG2A,MIR6750,MIR6749,PPP2R5B,GPHA2,C11orf85,BATF2,ARL2-SNX15,ARL2,MIR6879,SNX15,SAC3D1,NAALADL1* |
| rs654772 | 11 | 64731438 | *EHD1,MIR192,MIR194-2,ATG2A,MIR6750,MIR6749,PPP2R5B,GPHA2,C11orf85,BATF2,ARL2-SNX15,ARL2,MIR6879,SNX15,SAC3D1,NAALADL1* |
| rs659122 | 11 | 64738420 | *EHD1,MIR192,MIR194-2,ATG2A,MIR6750,MIR6749,PPP2R5B,GPHA2,C11orf85,BATF2,ARL2-SNX15,ARL2,MIR6879,SNX15,SAC3D1,NAALADL1* |
| rs12791871 | 11 | 71164544 | *FLJ42102,DHCR7,NADSYN1,MIR6754,KRTAP5-7,KRTAP5-8,KRTAP5-9* |
| rs7944926 | 11 | 71165625 | *FLJ42102,DHCR7,NADSYN1,MIR6754,KRTAP5-7,KRTAP5-8,KRTAP5-9* |
| rs12785878 | 11 | 71167449 | *FLJ42102,DHCR7,NADSYN1,MIR6754,KRTAP5-7,KRTAP5-8,KRTAP5-9* |
| rs10793294 | 11 | 77996403 | *KCTD21,USP35,GAB2* |
| rs10047420 | 11 | 115329152 | *CADM1* |
| rs11183053 | 12 | 45850656 | *ANO6* |
| rs7964616 | 12 | 45893296 | *ANO6* |
| rs1410816 | 13 | 32094193 |  |
| rs7322922 | 13 | 72319865 | *DACH1* |
| rs2414433 | 15 | 56044189 | *PRTG,NEDD4* |
| rs191368 | 15 | 64348858 | *DAPK2,FAM96A,SNX1,SNX22,PPIB* |
| rs1531163 | 15 | 75113083 | *CYP1A1,CYP1A2,CSK,MIR4513,LMAN1L,CPLX3,ULK3,MIR6882,SCAMP2,MPI,FAM219B,COX5A* |
| rs7163390 | 15 | 75280426 | *MPI,FAM219B,COX5A,RPP25,SCAMP5,PPCDC* |
| rs6495135 | 15 | 75295030 | *FAM219B,COX5A,RPP25,SCAMP5,PPCDC* |
| rs7174129 | 15 | 75305040 | *COX5A,RPP25,SCAMP5,PPCDC* |
| rs7342601 | 15 | 75306795 | *COX5A,RPP25,SCAMP5,PPCDC* |
| rs7342591 | 15 | 75306863 | *COX5A,RPP25,SCAMP5,PPCDC* |
| rs7500910 | 16 | 12385491 | *SNX29* |
| rs1542421 | 16 | 17270944 | *XYLT1* |
| rs7190310 | 16 | 17271862 | *XYLT1* |
| rs8044999 | 16 | 28974658 | *SH2B1,ATP2A1,LOC100289092,RABEP2,CD19,NFATC2IP,MIR4517,SPNS1,LAT* |
| rs9924445 | 16 | 86746818 |  |
| rs3818717 | 17 | 17707105 | *RAI1,SMCR5,SREBF1,MIR6777,MIR33B,TOM1L2* |
| rs11657423 | 17 | 17728574 | *RAI1,SMCR5,SREBF1,MIR6777,MIR33B,TOM1L2* |
| rs9907246 | 17 | 17770965 | *RAI1,SMCR5,SREBF1,MIR6777,MIR33B,TOM1L2* |
| rs950966 | 17 | 17783748 | *RAI1,SREBF1,MIR6777,MIR33B,TOM1L2,LRRC48* |
| rs4925123 | 17 | 17784374 | *RAI1,SREBF1,MIR6777,MIR33B,TOM1L2,LRRC48* |
| rs11650649 | 17 | 17790319 | *RAI1,SREBF1,MIR6777,MIR33B,TOM1L2,LRRC48* |
| rs8070128 | 17 | 17804725 | *RAI1,SREBF1,MIR6777,MIR33B,TOM1L2,LRRC48* |
| rs4925129 | 17 | 17807186 | *RAI1,SREBF1,MIR6777,MIR33B,TOM1L2,LRRC48* |
| rs8078138 | 17 | 17811251 | *RAI1,SREBF1,MIR6777,MIR33B,TOM1L2,LRRC48* |
| rs7222480 | 17 | 17818817 | *SREBF1,TOM1L2,LRRC48* |
| rs5002487 | 17 | 17832460 | *SREBF1,TOM1L2,LRRC48,ATPAF2* |
| rs8080823 | 17 | 17864048 | *TOM1L2,LRRC48,ATPAF2,GID4* |
| rs2955382 | 17 | 17947710 | *TOM1L2,LRRC48,ATPAF2,GID4,DRG2,MYO15A* |
| rs4925138 | 17 | 17992793 | *LRRC48,ATPAF2,GID4,DRG2,MYO15A,ALKBH5* |
| rs854813 | 17 | 18003845 | *LRRC48,ATPAF2,GID4,DRG2,MYO15A,ALKBH5* |
| rs8064539 | 17 | 18903576 | *PRPSAP2,SLC5A10,FAM83G,GRAP* |
| rs7211874 | 17 | 18904830 | *PRPSAP2,SLC5A10,FAM83G,GRAP* |
| rs5935245 | 23 | 12162126 | *FRMPD4* |
| rs1476595 | 23 | 13255459 | *GS1-600G8.3,ATXN3L,LINC01203* |
| rs2363130 | 23 | 23367895 | *PTCHD1-AS,PTCHD1* |
| rs2707160 | 23 | 23923684 | *APOO,CXorf58,KLHL15* |
| rs2520229 | 23 | 23939679 | *APOO,CXorf58,KLHL15* |
| rs3813165 | 23 | 24005086 | *APOO,CXorf58,KLHL15,EIF2S3* |
| rs5970824 | 23 | 24041718 | *CXorf58,KLHL15,EIF2S3* |
| rs11094949 | 23 | 24104646 | *KLHL15,EIF2S3,ZFX-AS1,ZFX* |
| rs7877712 | 23 | 24145844 | *EIF2S3,ZFX-AS1,ZFX* |
| rs2040577 | 23 | 24257499 | *ZFX-AS1,ZFX,SUPT20HL2* |
| rs2040576 | 23 | 24260078 | *ZFX-AS1,ZFX,SUPT20HL2* |
| rs1947879 | 23 | 25218185 |  |
| rs6526422 | 23 | 25220716 |  |
| rs5986763 | 23 | 25261728 |  |
| rs5986769 | 23 | 25276181 |  |
| rs5944148 | 23 | 25776639 |  |
| rs2079521 | 23 | 30320215 | *MAGEB2,MAGEB3,MAGEB4,MAGEB1,NR0B1* |
| rs6632458 | 23 | 36031808 | *CXorf22,LOC101928564,CHDC2* |
| rs6632464 | 23 | 36057261 | *CXorf22,LOC101928564,CHDC2* |
| rs5991678 | 23 | 43396447 |  |
| rs5936657 | 23 | 68347670 | *PJA1,LINC00269* |
| rs6525292 | 23 | 68534053 |  |
| rs12010107 | 23 | 71503284 | *PIN4,ERCC6L,RPS4X,CITED1,HDAC8* |
| rs2984344 | 23 | 71523614 | *PIN4,ERCC6L,RPS4X,CITED1,HDAC8* |
| rs3012658 | 23 | 71566745 | *PIN4,RPS4X,CITED1,HDAC8* |
| rs5987542 | 23 | 102798895 | *RAB40A,TCEAL4,TCEAL3,TCEAL1* |
| rs6413651 | 23 | 108332533 |  |
| rs4893413 | 23 | 108339383 |  |
| rs5985679 | 23 | 108363694 |  |
| rs6642615 | 23 | 108513405 |  |
| rs1205524 | 23 | 108544388 | *GUCY2F* |
| rs471900 | 23 | 108545876 | *GUCY2F* |
| rs504284 | 23 | 108547100 | *GUCY2F* |
| rs669418 | 23 | 108563970 | *GUCY2F* |
| rs668981 | 23 | 108564053 | *GUCY2F* |
| rs618821 | 23 | 108580639 | *GUCY2F* |
| rs5911899 | 23 | 116595019 |  |
| rs2843525 | 23 | 123696648 | *TENM1* |
| rs2858405 | 23 | 123716255 | *TENM1* |
| rs5976361 | 23 | 138469749 | *SRD5A1P1* |
| rs3848924 | 23 | 138486733 | *SRD5A1P1* |
| rs2367651 | 23 | 138486815 | *SRD5A1P1* |
| rs3859961 | 23 | 138487372 | *SRD5A1P1* |
| rs3903643 | 23 | 138487531 | *SRD5A1P1* |
| rs3903645 | 23 | 138487695 | *SRD5A1P1* |
| rs4083445 | 23 | 138488800 | *SRD5A1P1* |
| rs5908107 | 23 | 141136519 |  |
| rs6571298 | 23 | 152704232 | *ZNF275,ZFP92,TREX2,HAUS7,BGN,ATP2B3* |
| rs4646263 | 23 | 153140449 | *PLXNB3,SRPK3,IDH3G,SSR4,PDZD4,L1CAM,AVPR2,ARHGAP4,NAA10,RENBP,HCFC1,HCFC1-AS1,TMEM187* |
| rs5945384 | 23 | 153260414 | *AVPR2,ARHGAP4,NAA10,RENBP,HCFC1,HCFC1-AS1,TMEM187,MIR3202-1,MIR3202-2,IRAK1,MIR718,MECP2* |

**Table S5c. SNPs and genes identified using the 85^th^/15^th^ percentile ET cutoffs among CEU-GIH-YRI**

| Marker | Chromosome | Base pair position (Build 37) | Genes within 100 Kb |
| --- | --- | --- | --- |
| rs761087 | 1 | 11656684 | PTCHD2,LOC101929181,FBXO2,FBXO44,FBXO6,MAD2L2,DRAXIN |
| rs10779763 | 1 | 11690766 | PTCHD2,LOC101929181,FBXO2,FBXO44,FBXO6,MAD2L2,DRAXIN |
| rs2335768 | 1 | 11691161 | PTCHD2,LOC101929181,FBXO2,FBXO44,FBXO6,MAD2L2,DRAXIN |
| rs4846022 | 1 | 11697182 | PTCHD2,LOC101929181,FBXO2,FBXO44,FBXO6,MAD2L2,DRAXIN,AGTRAP |
| rs3013047 | 1 | 13830577 | PRAMEF20,PRAMEF21,LRRC38,PDPN |
| rs942841 | 1 | 39383166 | RRAGC,MYCBP,GJA9-MYCBP,GJA9,RHBDL2,AKIRIN1 |
| rs1555745 | 1 | 39483129 | RHBDL2,AKIRIN1,NDUFS5,MACF1 |
| rs1555741 | 1 | 39493121 | RHBDL2,AKIRIN1,NDUFS5,MACF1 |
| rs7554809 | 1 | 39503878 | RHBDL2,AKIRIN1,NDUFS5,MACF1 |
| rs839769 | 1 | 43872067 | TIE1,MPL,CDC20,ELOVL1,MIR6734,MED8,SZT2,MIR6735,HYI |
| rs2782646 | 1 | 43892189 | MPL,CDC20,ELOVL1,MIR6734,MED8,SZT2,MIR6735,HYI |
| rs2782648 | 1 | 43903589 | MPL,CDC20,ELOVL1,MIR6734,MED8,SZT2,MIR6735,HYI,PTPRF |
| rs2782650 | 1 | 43912590 | MPL,CDC20,ELOVL1,MIR6734,MED8,SZT2,MIR6735,HYI,PTPRF |
| rs2251804 | 1 | 43917409 | MPL,CDC20,ELOVL1,MIR6734,MED8,SZT2,MIR6735,HYI,PTPRF |
| rs607945 | 1 | 47212694 | ATPAF1,TEX38,EFCAB14-AS1,EFCAB14,CYP4B1,CYP4Z2P |
| rs594387 | 1 | 47213386 | ATPAF1,TEX38,EFCAB14-AS1,EFCAB14,CYP4B1,CYP4Z2P |
| rs6696001 | 1 | 54614145 | TMEM59,TCEANC2,MIR4781,CDCP2,CYB5RL,MRPL37,SSBP3,SSBP3-AS1 |
| rs10493362 | 1 | 65116332 | CACHD1,MIR4794,RAVER2 |
| rs11208492 | 1 | 65128375 | CACHD1,MIR4794,RAVER2 |
| rs11208493 | 1 | 65128395 | CACHD1,MIR4794,RAVER2 |
| rs11208496 | 1 | 65130601 | CACHD1,MIR4794,RAVER2 |
| rs7513574 | 1 | 65135341 | CACHD1,MIR4794,RAVER2 |
| rs6697088 | 1 | 67044724 | SGIP1,MIR3117 |
| rs6656912 | 1 | 67083671 | SGIP1,MIR3117 |
| rs9662943 | 1 | 67121132 | SGIP1,MIR3117,TCTEX1D1 |
| rs4655650 | 1 | 67142286 | SGIP1,MIR3117,TCTEX1D1 |
| rs11487952 | 1 | 75195812 | ERICH3,CRYZ,TYW3 |
| rs12738201 | 1 | 75275749 | CRYZ,TYW3 |
| rs6693427 | 1 | 77477794 | ST6GALNAC5,MIR7156,PIGK |
| rs497281 | 1 | 114720431 | SYT6 |
| rs35263946 | 1 | 116064403 |  |
| rs2495395 | 1 | 151662113 | SNX27,CELF3,RIIAD1,MRPL9,OAZ3,TDRKH |
| rs11588419 | 1 | 162831317 | DDR2,HSD17B7,C1orf110 |
| rs9426876 | 1 | 168259677 | TIPRL,SFT2D2,ANKRD36BP1,TBX19,MIR557 |
| rs502612 | 1 | 170641803 | PRRX1 |
| rs593479 | 1 | 170642899 | PRRX1 |
| rs12090950 | 1 | 201429381 | TNNT2,LAD1,TNNI1,PHLDA3,CSRP1,RPS10P7 |
| rs515384 | 1 | 201464807 | LAD1,TNNI1,PHLDA3,CSRP1,RPS10P7 |
| rs6661833 | 1 | 202037007 | TIMM17A,RNPEP,MIR6740,ELF3,GPR37L1,ARL8A,PTPN7 |
| rs11576867 | 1 | 205064676 | NFASC,CNTN2,TMEM81,RBBP5,DSTYK |
| rs2153904 | 1 | 205642790 | MFSD4,ELK4,SLC45A3,NUCKS1,RAB7L1 |
| rs11120481 | 1 | 215240411 | KCNK2 |
| rs1557174 | 1 | 215694431 | KCTD3 |
| rs14137 | 1 | 215793834 | KCTD3,USH2A |
| rs973997 | 1 | 218890950 |  |
| rs1481356 | 1 | 218908668 |  |
| rs271738 | 1 | 234662890 | TARBP1,LOC100506795,IRF2BP2 |
| rs1543561 | 1 | 236180108 | NID1 |
| rs2872977 | 2 | 4997979 |  |
| rs6605281 | 2 | 5000802 |  |
| rs2693818 | 2 | 6204508 | LINC01105,LOC400940 |
| rs1524007 | 2 | 13699487 |  |
| rs6419085 | 2 | 18286852 |  |
| rs7422512 | 2 | 18439946 |  |
| rs6707939 | 2 | 28487706 | BRE,LOC100505716 |
| rs2005181 | 2 | 28549757 | BRE,LOC100505716,FLJ31356,FOSL2 |
| rs11127142 | 2 | 28559717 | BRE,LOC100505716,FLJ31356,FOSL2 |
| rs6732543 | 2 | 28562703 | BRE,LOC100505716,FLJ31356,FOSL2 |
| rs7578465 | 2 | 29743733 | ALK |
| rs17015608 | 2 | 35005992 |  |
| rs1432295 | 2 | 61066666 | PAPOLG,LINC01185,REL |
| rs6726160 | 2 | 61164729 | LINC01185,REL,PUS10,PEX13 |
| rs2970889 | 2 | 88411904 | KRCC1,SMYD1,MIR4780,FABP1,THNSL2 |
| rs12467940 | 2 | 123065343 |  |
| rs1459166 | 2 | 126034009 |  |
| rs1436303 | 2 | 126159025 |  |
| rs1836858 | 2 | 126159556 |  |
| rs1436304 | 2 | 126166731 |  |
| rs10200381 | 2 | 126168988 |  |
| rs12472307 | 2 | 126201064 |  |
| rs6710520 | 2 | 126288060 |  |
| rs539588 | 2 | 135212329 | MGAT5,TMEM163 |
| rs544050 | 2 | 135212819 | MGAT5,TMEM163 |
| rs503562 | 2 | 135260071 | MGAT5,TMEM163 |
| rs599076 | 2 | 135263902 | MGAT5,TMEM163 |
| rs669746 | 2 | 135274766 | MGAT5,TMEM163 |
| rs624817 | 2 | 135278207 | MGAT5,TMEM163 |
| rs6430545 | 2 | 135602601 | ACMSD,CCNT2-AS1,CCNT2 |
| rs2053726 | 2 | 135610154 | ACMSD,CCNT2-AS1,CCNT2 |
| rs1446523 | 2 | 135616621 | ACMSD,CCNT2-AS1,CCNT2 |
| rs6706537 | 2 | 135618982 | ACMSD,CCNT2-AS1,CCNT2 |
| rs7593370 | 2 | 135623517 | ACMSD,CCNT2-AS1,CCNT2,MAP3K19 |
| rs12469941 | 2 | 135629927 | ACMSD,CCNT2-AS1,CCNT2,MAP3K19 |
| rs1446524 | 2 | 135631331 | ACMSD,CCNT2-AS1,CCNT2,MAP3K19 |
| rs2166480 | 2 | 135637338 | ACMSD,CCNT2-AS1,CCNT2,MAP3K19 |
| rs4954193 | 2 | 135642180 | ACMSD,CCNT2-AS1,CCNT2,MAP3K19 |
| rs7589297 | 2 | 135652399 | ACMSD,CCNT2-AS1,CCNT2,MAP3K19 |
| rs12469411 | 2 | 135664556 | ACMSD,CCNT2-AS1,CCNT2,MAP3K19 |
| rs1374289 | 2 | 135694379 | ACMSD,CCNT2-AS1,CCNT2,MAP3K19 |
| rs3814355 | 2 | 135711516 | ACMSD,CCNT2-AS1,CCNT2,MAP3K19,RAB3GAP1 |
| rs1530559 | 2 | 135755629 | ACMSD,CCNT2-AS1,CCNT2,MAP3K19,RAB3GAP1 |
| rs309164 | 2 | 136691825 | LCT,MCM6,DARS,LOC101928243 |
| rs12615624 | 2 | 136721603 | MCM6,DARS,LOC101928243 |
| rs309134 | 2 | 136755684 | DARS,LOC101928243 |
| rs13392503 | 2 | 137431134 |  |
| rs6751451 | 2 | 154731306 | GALNT13 |
| rs1348588 | 2 | 154731612 | GALNT13 |
| rs1348587 | 2 | 154731793 | GALNT13 |
| rs7576957 | 2 | 154846176 | GALNT13 |
| rs7580732 | 2 | 154847177 | GALNT13 |
| rs10490534 | 2 | 154937930 | GALNT13 |
| rs10497204 | 2 | 159950898 | TANC1,MIR6888 |
| rs6433083 | 2 | 169499314 | CERS6,MIR4774 |
| rs7591406 | 2 | 169499335 | CERS6,MIR4774 |
| rs6757373 | 2 | 169499352 | CERS6,MIR4774 |
| rs1834271 | 2 | 169506219 | CERS6,MIR4774 |
| rs7584977 | 2 | 173332725 | ITGA6,PDK1 |
| rs2303537 | 2 | 179366093 | MIR548N,LOC101927027,PRKRA,DFNB59,FKBP7,PLEKHA3,TTN-AS1,TTN |
| rs2293554 | 2 | 202131587 | CFLAR,CASP10,CASP8,ALS2CR12 |
| rs7607406 | 2 | 207579995 | ADAM23,LOC200726,DYTN,MDH1B,FASTKD2,MIR3130-2,MIR3130-1 |
| rs1519017 | 2 | 216486353 | LINC00607 |
| rs4586623 | 2 | 233939615 | NGEF,NEU2,INPP5D |
| rs12639107 | 3 | 1018269 |  |
| rs1682914 | 3 | 3844751 | LRRN1 |
| rs1682913 | 3 | 3846526 | LRRN1 |
| rs9857030 | 3 | 4636821 | ITPR1 |
| rs4073664 | 3 | 4682456 | ITPR1 |
| rs4142942 | 3 | 4841657 | ITPR1,EGOT,BHLHE40-AS1 |
| rs4685934 | 3 | 5490593 |  |
| rs2574720 | 3 | 11660412 | ATG7,VGLL4 |
| rs4132228 | 3 | 64708114 | ADAMTS9,ADAMTS9-AS2,MIR548A2 |
| rs6784514 | 3 | 71319746 | FOXP1 |
| rs2398878 | 3 | 103372647 |  |
| rs1839077 | 3 | 103745341 |  |
| rs9816529 | 3 | 124905511 | SLC12A8,MIR5092,ZNF148 |
| rs11920780 | 3 | 134642667 | EPHB1 |
| rs9830186 | 3 | 149184314 | TM4SF1,TM4SF1-AS1,TM4SF4,WWTR1 |
| rs12485821 | 3 | 149185990 | TM4SF1,TM4SF1-AS1,TM4SF4,WWTR1 |
| rs11928696 | 3 | 149188538 | TM4SF1,TM4SF1-AS1,TM4SF4,WWTR1 |
| rs4681521 | 3 | 149230137 | TM4SF4,WWTR1 |
| rs13086215 | 3 | 150427673 | SELT,ERICH6,SIAH2 |
| rs6778736 | 3 | 153220021 | C3orf79 |
| rs2922407 | 3 | 158967089 | IQCJ-SCHIP1,IQCJ,SCHIP1,MIR3919 |
| rs7624604 | 3 | 158975626 | IQCJ-SCHIP1,IQCJ,SCHIP1,MIR3919 |
| rs1449009 | 3 | 158977883 | IQCJ-SCHIP1,IQCJ,SCHIP1,MIR3919 |
| rs3911092 | 3 | 168306363 | EGFEM1P,MIR551B |
| rs520418 | 3 | 172138843 | FNDC3B,GHSR,TNFSF10 |
| rs4593055 | 3 | 172147586 | FNDC3B,GHSR,TNFSF10 |
| rs2442057 | 3 | 172149691 | FNDC3B,GHSR,TNFSF10 |
| rs509505 | 3 | 172153170 | FNDC3B,GHSR,TNFSF10 |
| rs530893 | 3 | 172155963 | FNDC3B,GHSR,TNFSF10 |
| rs545495 | 3 | 174351626 |  |
| rs9864104 | 3 | 185357531 | LIPH,SENP2,IGF2BP2,C3orf65 |
| rs7633866 | 3 | 187936974 | LPP-AS2,LPP,FLJ42393 |
| rs7623253 | 3 | 188405480 | LPP |
| rs6784434 | 3 | 192079832 | FGF12 |
| rs980797 | 4 | 24723481 | SOD3,CCDC149 |
| rs2130249 | 4 | 26504912 | RBPJ,CCKAR,TBC1D19 |
| rs4833022 | 4 | 38251292 |  |
| rs7667070 | 4 | 38379833 | LINC01258 |
| rs13149231 | 4 | 38716483 | KLF3-AS1,KLF3,TLR10,TLR1 |
| rs1481816 | 4 | 54218672 | SCFD2,FIP1L1 |
| rs10024153 | 4 | 54251658 | SCFD2,FIP1L1,LNX1 |
| rs10014172 | 4 | 54251718 | SCFD2,FIP1L1,LNX1 |
| rs10025091 | 4 | 54264543 | SCFD2,FIP1L1,LNX1 |
| rs11133274 | 4 | 54299380 | SCFD2,FIP1L1,LNX1,LNX1-AS1 |
| rs6810557 | 4 | 54351687 | FIP1L1,LNX1,LNX1-AS1 |
| rs6554109 | 4 | 54352035 | FIP1L1,LNX1,LNX1-AS1 |
| rs7656012 | 4 | 57696652 | SPINK2,REST |
| rs12645408 | 4 | 60879952 |  |
| rs2165482 | 4 | 61011853 |  |
| rs11131228 | 4 | 61080750 |  |
| rs9996745 | 4 | 61115698 |  |
| rs717253 | 4 | 61163846 |  |
| rs6827408 | 4 | 61177831 |  |
| rs6848703 | 4 | 61181195 |  |
| rs6857452 | 4 | 77317124 | FAM47E-STBD1,STBD1,CCDC158,SHROOM3 |
| rs4272041 | 4 | 77330682 | FAM47E-STBD1,STBD1,CCDC158,SHROOM3 |
| rs1922286 | 4 | 80357377 | GK2,LINC00989 |
| rs994285 | 4 | 86659114 | ARHGAP24,MIR4451 |
| rs10516755 | 4 | 86666041 | ARHGAP24,MIR4451 |
| rs2589511 | 4 | 86925912 | ARHGAP24,MAPK10 |
| rs2604209 | 4 | 89621072 | HERC3,NAP1L5,FAM13A-AS1,FAM13A |
| rs13130 | 4 | 89648845 | HERC3,NAP1L5,FAM13A-AS1,FAM13A |
| rs12510722 | 4 | 100147101 | LOC100507053,ADH4,PCNAP1,ADH6,ADH1A,ADH1B |
| rs2173199 | 4 | 100171379 | LOC100507053,PCNAP1,ADH6,ADH1A,ADH1B,ADH1C |
| rs6532814 | 4 | 100173968 | LOC100507053,PCNAP1,ADH6,ADH1A,ADH1B,ADH1C |
| rs1354856 | 4 | 134620098 |  |
| rs1605424 | 4 | 134621563 |  |
| rs6535730 | 4 | 151264359 | DCLK2,LRBA |
| rs6823091 | 4 | 153069783 |  |
| rs7671283 | 4 | 153076053 |  |
| rs7684789 | 4 | 188031286 |  |
| rs9995329 | 4 | 188032207 |  |
| rs28628755 | 4 | 188915984 | ZFP42,TRIML2 |
| rs2641215 | 5 | 2355878 | LOC100506858 |
| rs12522439 | 5 | 2775889 | IRX2,C5orf38 |
| rs460083 | 5 | 3080520 | LOC102467074 |
| rs7713096 | 5 | 4590072 |  |
| rs4702858 | 5 | 4614313 |  |
| rs11949458 | 5 | 23385700 |  |
| rs2914255 | 5 | 23465827 | PRDM9 |
| rs35391 | 5 | 33955673 | ADAMTS12,RXFP3,SLC45A2,AMACR,C1QTNF3-AMACR,C1QTNF3 |
| rs28117 | 5 | 33962770 | ADAMTS12,RXFP3,SLC45A2,AMACR,C1QTNF3-AMACR,C1QTNF3 |
| rs149359 | 5 | 34035240 | RXFP3,SLC45A2,AMACR,C1QTNF3-AMACR,C1QTNF3 |
| rs13160147 | 5 | 36191407 | LMBRD2,MIR580,SKP2,NADK2,RANBP3L |
| rs1320306 | 5 | 76174519 | F2RL1,S100Z,CRHBP |
| rs1978837 | 5 | 105949415 |  |
| rs4429894 | 5 | 109499203 |  |
| rs11744416 | 5 | 109513342 |  |
| rs4246005 | 5 | 109515646 |  |
| rs1438673 | 5 | 110467499 | TSLP,WDR36,CAMK4 |
| rs2407398 | 5 | 122012354 | LOC101927379,SNX2 |
| rs6885488 | 5 | 122026428 | LOC101927379,SNX2 |
| rs17622208 | 5 | 131717050 | SLC22A4,LOC553103,MIR3936,SLC22A5,C5orf56 |
| rs10079542 | 5 | 171269021 | SMIM23,FBXW11 |
| rs2434195 | 5 | 174341887 | FLJ16171 |
| rs2731672 | 5 | 176842474 | LMAN2,RGS14,SLC34A1,PFN3,F12,GRK6,PRR7-AS1,PRR7,DBN1,PDLIM7,DOK3,DDX41 |
| rs751546 | 5 | 178623518 | ADAMTS2 |
| rs13219486 | 6 | 15801167 |  |
| rs1511467 | 6 | 23918873 |  |
| rs12215330 | 6 | 38270915 | BTBD9 |
| rs12208912 | 6 | 38272883 | BTBD9 |
| rs7748599 | 6 | 38363213 | BTBD9 |
| rs4711546 | 6 | 38366186 | BTBD9 |
| rs11961988 | 6 | 53883240 | LRRC1,MLIP-IT1,MLIP |
| rs16884583 | 6 | 53883490 | LRRC1,MLIP-IT1,MLIP |
| rs10484653 | 6 | 53884126 | LRRC1,MLIP-IT1,MLIP |
| rs10484652 | 6 | 53884210 | LRRC1,MLIP-IT1,MLIP |
| rs1570912 | 6 | 53885918 | LRRC1,MLIP-IT1,MLIP |
| rs9442831 | 6 | 73360633 | KCNQ5 |
| rs1408766 | 6 | 102198914 | GRIK2 |
| rs864691 | 6 | 107944495 | SOBP,SCML4 |
| rs7771618 | 6 | 111131329 | CDK19,AMD1 |
| rs476366 | 6 | 128044051 | THEMIS |
| rs1970352 | 6 | 143861900 | ADAT2,PEX3,FUCA2,LOC285740,PHACTR2 |
| rs17368239 | 6 | 143934587 | LOC285740,PHACTR2 |
| rs10872705 | 6 | 154616419 | OPRM1,IPCEF1 |
| rs2178628 | 7 | 3613667 | SDK1 |
| rs2041407 | 7 | 14861101 | DGKB |
| rs17370286 | 7 | 14916881 | DGKB |
| rs7787411 | 7 | 14972884 | DGKB |
| rs1474347 | 7 | 22768124 | IL6,TOMM7 |
| rs10229457 | 7 | 22800800 | IL6,TOMM7,SNORD93 |
| rs10254065 | 7 | 26388552 | SNX10,LOC441204 |
| rs17171385 | 7 | 38532286 | AMPH |
| rs39200 | 7 | 89734057 | STEAP2-AS1,DPY19L2P4,STEAP1 |
| rs39207 | 7 | 89735499 | STEAP2-AS1,DPY19L2P4,STEAP1 |
| rs6957658 | 7 | 95897563 | SLC25A13,MIR591 |
| rs799618 | 7 | 110485378 | IMMP2L |
| rs725272 | 7 | 110586127 | IMMP2L |
| rs2041531 | 7 | 110606862 | IMMP2L |
| rs7796806 | 7 | 110618415 | IMMP2L |
| rs12673425 | 7 | 113541607 | PPP1R3A |
| rs6974649 | 7 | 130813097 | LINC-PINT,MKLN1 |
| rs6947916 | 7 | 130815342 | LINC-PINT,MKLN1 |
| rs2536067 | 7 | 151425449 | PRKAG2 |
| rs3843924 | 8 | 4078053 | CSMD1 |
| rs1714821 | 8 | 4084879 | CSMD1 |
| rs13277114 | 8 | 8807288 | MFHAS1,ERI1,MIR4660 |
| rs17751178 | 8 | 10116730 | MSRA |
| rs9969635 | 8 | 10331636 | MSRA,PRSS55 |
| rs11784167 | 8 | 12623671 | LONRF1,MIR3926-1,MIR3926-2,LOC340357,LINC00681 |
| rs6530980 | 8 | 12633235 | LONRF1,MIR3926-1,MIR3926-2,LOC340357,LINC00681 |
| rs10101001 | 8 | 17041428 | MICU3,ZDHHC2,CNOT7,VPS37A |
| rs17506026 | 8 | 17556514 | PDGFRL,MTUS1 |
| rs7460727 | 8 | 17640706 | MTUS1,FGL1 |
| rs739341 | 8 | 17708154 | MTUS1,FGL1,PCM1 |
| rs13273924 | 8 | 33126999 |  |
| rs4422800 | 8 | 49536901 | LOC101929268,LOC101929217,EFCAB1 |
| rs6471945 | 8 | 49563320 | LOC101929268,LOC101929217,EFCAB1 |
| rs7011862 | 8 | 49817333 | SNAI2 |
| rs10504155 | 8 | 54429080 |  |
| rs1483538 | 8 | 54766847 | ATP6V1H,RGS20 |
| rs7827611 | 8 | 54788251 | ATP6V1H,RGS20,TCEA1 |
| rs1384797 | 8 | 54793928 | ATP6V1H,RGS20,TCEA1 |
| rs11991952 | 8 | 55069061 | LYPLA1,MRPL15 |
| rs10435604 | 8 | 62152674 | CLVS1 |
| rs3864670 | 8 | 62152901 | CLVS1 |
| rs3864671 | 8 | 62153049 | CLVS1 |
| rs6980924 | 8 | 62156050 | CLVS1 |
| rs9643526 | 8 | 62167697 | CLVS1 |
| rs2919308 | 8 | 62195086 | CLVS1 |
| rs3852344 | 8 | 62207427 | CLVS1 |
| rs4562361 | 8 | 66456252 | LOC286186,ARMC1 |
| rs4281146 | 8 | 66457596 | LOC286186,ARMC1,MTFR1 |
| rs12541366 | 8 | 97398842 | PTDSS1 |
| rs17709854 | 8 | 97399572 | PTDSS1 |
| rs750500 | 8 | 97402724 | PTDSS1 |
| rs7813186 | 8 | 110856481 |  |
| rs10110311 | 8 | 112327334 |  |
| rs1508557 | 8 | 118730636 | EXT1 |
| rs5010752 | 8 | 122061168 |  |
| rs7835412 | 8 | 122061527 |  |
| rs10505389 | 8 | 122062152 |  |
| rs2043375 | 8 | 122096261 |  |
| rs4301437 | 8 | 125460073 | TMEM65,TRMT12,RNF139-AS1,RNF139,TATDN1,MIR6844,NDUFB9 |
| rs3750232 | 8 | 125570528 | RNF139-AS1,RNF139,TATDN1,MIR6844,NDUFB9,MTSS1 |
| rs10959088 | 9 | 10390416 | PTPRD |
| rs10121181 | 9 | 12637203 | TYRP1 |
| rs10960928 | 9 | 13077072 | MPDZ |
| rs484555 | 9 | 14085583 | NFIB |
| rs10810590 | 9 | 16689291 | BNC2 |
| rs10511625 | 9 | 16693969 | BNC2 |
| rs10511624 | 9 | 16695690 | BNC2 |
| rs1888207 | 9 | 16696187 | BNC2 |
| rs10738446 | 9 | 16696510 | BNC2 |
| rs10962542 | 9 | 16698234 | BNC2 |
| rs10810593 | 9 | 16699008 | BNC2 |
| rs2297176 | 9 | 16706012 | BNC2 |
| rs10810611 | 9 | 16756377 | BNC2 |
| rs7026582 | 9 | 20287948 | MLLT3 |
| rs683946 | 9 | 25924754 |  |
| rs11141229 | 9 | 88707719 | NAA35,GOLM1,LOC101927623 |
| rs4877118 | 9 | 92258048 | GADD45G,UNQ6494 |
| rs10819700 | 9 | 102635520 | LOC101928438,NR4A3,LOC441461,STX17 |
| rs2416939 | 9 | 102648036 | LOC101928438,NR4A3,LOC441461,STX17,ERP44 |
| rs7024182 | 9 | 102654447 | LOC101928438,NR4A3,LOC441461,STX17,ERP44 |
| rs6477728 | 9 | 112539661 | PALM2,PALM2-AKAP2 |
| rs10984107 | 9 | 121355679 |  |
| rs563864 | 9 | 135955672 | GFI1B,GTF3C5,MIR6877,CEL,CELP,RALGDS,GBGT1 |
| rs11254339 | 10 | 17081153 | CUBN |
| rs7079066 | 10 | 18286639 | MRC1,SLC39A12,LOC100129213 |
| rs943335 | 10 | 18292493 | MRC1,SLC39A12,LOC100129213 |
| rs2007909 | 10 | 18292621 | MRC1,SLC39A12,LOC100129213 |
| rs220366 | 10 | 24592764 | KIAA1217,MIR603 |
| rs7086023 | 10 | 28384098 | ARMC4,MPP7 |
| rs11006992 | 10 | 28582697 | MPP7,MIR8086 |
| rs306583 | 10 | 30710057 | MTPAP,MIR7162,MAP3K8 |
| rs2175918 | 10 | 50231840 | WDFY4,MIR4294,VSTM4,FAM170B-AS1 |
| rs10740279 | 10 | 69360766 | CTNNA3 |
| rs11000828 | 10 | 75733299 | CAMK2G,C10orf55,PLAU,VCL |
| rs2579785 | 10 | 78066337 | C10orf11 |
| rs772830 | 10 | 80616723 | ZMIZ1-AS1 |
| rs2148022 | 10 | 91901364 |  |
| rs10785942 | 10 | 91902898 |  |
| rs7923831 | 10 | 91904968 |  |
| rs1274391 | 10 | 92186243 | LOC101926942 |
| rs2259433 | 10 | 94147300 | CPEB3,MARCH5,MARK2P9,IDE |
| rs2259430 | 10 | 94147345 | CPEB3,MARCH5,MARK2P9,IDE |
| rs3814163 | 10 | 98742605 | LCOR,C10orf12,SLIT1 |
| rs2418776 | 10 | 107545496 |  |
| rs7901993 | 10 | 108241234 | SORCS1 |
| rs11193407 | 10 | 109161645 |  |
| rs10787024 | 10 | 109171971 |  |
| rs10509835 | 10 | 109205283 |  |
| rs11193528 | 10 | 109287585 |  |
| rs1896413 | 10 | 123181038 | FGFR2 |
| rs11245258 | 10 | 126211692 | NKX1-2,LHPP,FAM53B |
| rs10902223 | 11 | 817786 | EPS8L2,TALDO1,PDDC1,NS3BP,CEND1,SLC25A22,PIDD,RPLP2,SNORA52,PNPLA2,EFCAB4A,CD151,POLR2L,TSPAN4,CHID1 |
| rs1138714 | 11 | 825110 | EPS8L2,TALDO1,PDDC1,NS3BP,CEND1,SLC25A22,PIDD,RPLP2,SNORA52,PNPLA2,EFCAB4A,CD151,POLR2L,TSPAN4,CHID1 |
| rs6597973 | 11 | 893299 | SLC25A22,PIDD,RPLP2,SNORA52,PNPLA2,EFCAB4A,CD151,POLR2L,TSPAN4,CHID1,AP2A2 |
| rs368019 | 11 | 5569528 | OR51B5,OR51I2,OR52D1,UBQLN3,UBQLNL,OR52H1,OR52B6,TRIM6,TRIM6-TRIM34,TRIM34 |
| rs4485112 | 11 | 11270088 | GALNT18 |
| rs2707100 | 11 | 19935832 | NAV2 |
| rs2061596 | 11 | 37187913 |  |
| rs11033999 | 11 | 37189731 |  |
| rs972353 | 11 | 61717715 | FADS2,FADS3,MIR6746,RAB3IL1,BEST1,FTH1 |
| rs2736597 | 11 | 61718036 | FADS2,FADS3,MIR6746,RAB3IL1,BEST1,FTH1 |
| rs1800007 | 11 | 61719387 | FADS2,FADS3,MIR6746,RAB3IL1,BEST1,FTH1 |
| rs1675131 | 11 | 61895741 | INCENP,SCGB1D1,SCGB2A1 |
| rs1792946 | 11 | 61896215 | INCENP,SCGB1D1,SCGB2A1 |
| rs1792944 | 11 | 61897197 | INCENP,SCGB1D1,SCGB2A1 |
| rs1675061 | 11 | 61898756 | INCENP,SCGB1D1,SCGB2A1 |
| rs1675062 | 11 | 61898859 | INCENP,SCGB1D1,SCGB2A1 |
| rs1675063 | 11 | 61900740 | INCENP,SCGB1D1,SCGB2A1 |
| rs586616 | 11 | 64707978 | CDC42BPG,EHD1,MIR192,MIR194-2,ATG2A,MIR6750,MIR6749,PPP2R5B,GPHA2,C11orf85,BATF2,ARL2-SNX15,ARL2,MIR6879,SNX15 |
| rs596321 | 11 | 64713485 | EHD1,MIR192,MIR194-2,ATG2A,MIR6750,MIR6749,PPP2R5B,GPHA2,C11orf85,BATF2,ARL2-SNX15,ARL2,MIR6879,SNX15,SAC3D1,NAALADL1 |
| rs602404 | 11 | 64726701 | EHD1,MIR192,MIR194-2,ATG2A,MIR6750,MIR6749,PPP2R5B,GPHA2,C11orf85,BATF2,ARL2-SNX15,ARL2,MIR6879,SNX15,SAC3D1,NAALADL1 |
| rs654772 | 11 | 64731438 | EHD1,MIR192,MIR194-2,ATG2A,MIR6750,MIR6749,PPP2R5B,GPHA2,C11orf85,BATF2,ARL2-SNX15,ARL2,MIR6879,SNX15,SAC3D1,NAALADL1 |
| rs659122 | 11 | 64738420 | EHD1,MIR192,MIR194-2,ATG2A,MIR6750,MIR6749,PPP2R5B,GPHA2,C11orf85,BATF2,ARL2-SNX15,ARL2,MIR6879,SNX15,SAC3D1,NAALADL1 |
| rs4944042 | 11 | 71118788 | FLJ42102,DHCR7,NADSYN1,MIR6754 |
| rs12791871 | 11 | 71164544 | FLJ42102,DHCR7,NADSYN1,MIR6754,KRTAP5-7,KRTAP5-8,KRTAP5-9 |
| rs7944926 | 11 | 71165625 | FLJ42102,DHCR7,NADSYN1,MIR6754,KRTAP5-7,KRTAP5-8,KRTAP5-9 |
| rs12785878 | 11 | 71167449 | FLJ42102,DHCR7,NADSYN1,MIR6754,KRTAP5-7,KRTAP5-8,KRTAP5-9 |
| rs2186778 | 11 | 71185518 | FLJ42102,DHCR7,NADSYN1,MIR6754,KRTAP5-7,KRTAP5-8,KRTAP5-9,KRTAP5-10 |
| rs3794060 | 11 | 71187679 | FLJ42102,DHCR7,NADSYN1,MIR6754,KRTAP5-7,KRTAP5-8,KRTAP5-9,KRTAP5-10 |
| rs4944998 | 11 | 71207205 | FLJ42102,DHCR7,NADSYN1,MIR6754,KRTAP5-7,KRTAP5-8,KRTAP5-9,KRTAP5-10,KRTAP5-11 |
| rs10793294 | 11 | 77996403 | KCTD21,USP35,GAB2 |
| rs7125819 | 11 | 115228297 | CADM1 |
| rs7947456 | 11 | 115280498 | CADM1 |
| rs220847 | 11 | 115286117 | CADM1 |
| rs7122693 | 11 | 115304363 | CADM1 |
| rs220859 | 11 | 115312071 | CADM1 |
| rs10047420 | 11 | 115329152 | CADM1 |
| rs1508099 | 11 | 116338514 |  |
| rs679736 | 11 | 126041204 | RPUSD4,FAM118B,SRPR,FOXRED1 |
| rs7120806 | 11 | 129277029 | BARX2 |
| rs11607460 | 11 | 129285912 | BARX2 |
| rs10842349 | 12 | 8976764 | RIMKLB,A2ML1,PHC1 |
| rs4768081 | 12 | 45519421 | DBX2,RACGAP1P,PLEKHA8P1,RNY5,ANO6 |
| rs1118582 | 12 | 45540934 | DBX2,RACGAP1P,PLEKHA8P1,RNY5,ANO6 |
| rs11183053 | 12 | 45850656 | ANO6 |
| rs7964616 | 12 | 45893296 | ANO6 |
| rs303817 | 12 | 52176235 | SCN8A,FIGNL2 |
| rs303816 | 12 | 52183485 | SCN8A,FIGNL2,ANKRD33 |
| rs303815 | 12 | 52184271 | SCN8A,FIGNL2,ANKRD33 |
| rs6580907 | 12 | 53169676 | KRT1,KRT77,KRT76,KRT3,KRT4,KRT79,KRT78 |
| rs10506321 | 12 | 54224324 | CISTR |
| rs12822602 | 12 | 54224680 | CISTR |
| rs11170706 | 12 | 54226257 | CISTR |
| rs7298991 | 12 | 54869149 | ZNF385A,ITGA5,GTSF1,NCKAP1L,PDE1B |
| rs1463652 | 12 | 55351215 | MUCL1,TESPA1,NEUROD4 |
| rs7978952 | 12 | 55397643 | TESPA1,NEUROD4 |
| rs4140779 | 12 | 68516129 | IFNG,IL26 |
| rs4334090 | 12 | 76313528 |  |
| rs1991853 | 12 | 76349064 | PHLDA1,NAP1L1 |
| rs10850923 | 12 | 118327525 | KSR2 |
| rs10850924 | 12 | 118327544 | KSR2 |
| rs830120 | 12 | 122427654 | PSMD9,WDR66,BCL7A,MLXIP |
| rs10848032 | 12 | 130681300 | FZD10-AS1,FZD10 |
| rs1359948 | 13 | 27472758 |  |
| rs9590972 | 13 | 31583488 | TEX26-AS1,MEDAG,TEX26 |
| rs277155 | 13 | 32088155 |  |
| rs1410816 | 13 | 32094193 |  |
| rs1006096 | 13 | 32189143 |  |
| rs9533297 | 13 | 43475191 | EPSTI1 |
| rs7982150 | 13 | 72237748 | DACH1 |
| rs9542732 | 13 | 72247171 | DACH1 |
| rs7322922 | 13 | 72319865 | DACH1 |
| rs9593517 | 13 | 81594077 |  |
| rs7335457 | 13 | 94324708 | GPC6 |
| rs11840833 | 13 | 94378307 | GPC6,GPC6-AS2 |
| rs9556321 | 13 | 94397620 | GPC6,GPC6-AS2 |
| rs12018446 | 13 | 100094840 | UBAC2,MIR548AN,FKSG29,MIR623,LINC01232,TM9SF2 |
| rs4325408 | 13 | 100115662 | UBAC2,MIR548AN,LINC01232,TM9SF2 |
| rs1253636 | 14 | 52421595 | GNG2,C14orf166,NID2 |
| rs4901520 | 14 | 55022454 | GMFB,CGRRF1,SAMD4A |
| rs9783664 | 14 | 55030847 | GMFB,CGRRF1,SAMD4A |
| rs1307289 | 14 | 55042615 | GMFB,CGRRF1,SAMD4A |
| rs2104718 | 14 | 57325374 | OTX2,OTX2-AS1 |
| rs2039082 | 14 | 61505657 | MNAT1,TRMT5,SLC38A6 |
| rs2039081 | 14 | 61506054 | MNAT1,TRMT5,SLC38A6 |
| rs8022194 | 14 | 66707190 |  |
| rs7145240 | 14 | 66708299 |  |
| rs1434901 | 14 | 66712342 |  |
| rs7160830 | 14 | 73139738 | DPF3 |
| rs4325488 | 14 | 86626254 | LOC101928767 |
| rs10131159 | 14 | 90313173 | EFCAB11 |
| rs7156674 | 14 | 90315328 | EFCAB11 |
| rs9972236 | 14 | 94571340 | LINC00521,OTUB2,DDX24,IFI27L1,IFI27,IFI27L2,PPP4R4 |
| rs10130697 | 14 | 94608442 | OTUB2,DDX24,IFI27L1,IFI27,IFI27L2,PPP4R4 |
| rs10138647 | 14 | 94612496 | OTUB2,DDX24,IFI27L1,IFI27,IFI27L2,PPP4R4 |
| rs10134615 | 14 | 94627188 | DDX24,IFI27L1,IFI27,IFI27L2,PPP4R4 |
| rs7149473 | 14 | 107223238 |  |
| rs2594894 | 15 | 28140844 | OCA2 |
| rs12442147 | 15 | 28292178 | OCA2,HERC2 |
| rs4778241 | 15 | 28338713 | OCA2,HERC2 |
| rs2414433 | 15 | 56044189 | PRTG,NEDD4 |
| rs28890483 | 15 | 59719169 | MYO1E,FAM81A |
| rs6494120 | 15 | 59894617 | FAM81A,GCNT3,GTF2A2,BNIP2 |
| rs6494121 | 15 | 59894636 | FAM81A,GCNT3,GTF2A2,BNIP2 |
| rs11637648 | 15 | 59895431 | FAM81A,GCNT3,GTF2A2,BNIP2 |
| rs191368 | 15 | 64348858 | DAPK2,FAM96A,SNX1,SNX22,PPIB |
| rs2062250 | 15 | 64672002 | CSNK1G1,KIAA0101,TRIP4 |
| rs547818 | 15 | 64866329 | ZNF609 |
| rs615278 | 15 | 64893542 | ZNF609,OAZ2 |
| rs662142 | 15 | 64912091 | ZNF609,OAZ2 |
| rs2249914 | 15 | 64926130 | ZNF609,OAZ2 |
| rs1531163 | 15 | 75113083 | CYP1A1,CYP1A2,CSK,MIR4513,LMAN1L,CPLX3,ULK3,MIR6882,SCAMP2,MPI,FAM219B,COX5A |
| rs7163390 | 15 | 75280426 | MPI,FAM219B,COX5A,RPP25,SCAMP5,PPCDC |
| rs6495135 | 15 | 75295030 | FAM219B,COX5A,RPP25,SCAMP5,PPCDC |
| rs7174129 | 15 | 75305040 | COX5A,RPP25,SCAMP5,PPCDC |
| rs7342601 | 15 | 75306795 | COX5A,RPP25,SCAMP5,PPCDC |
| rs7342591 | 15 | 75306863 | COX5A,RPP25,SCAMP5,PPCDC |
| rs13056 | 15 | 75312187 | COX5A,RPP25,SCAMP5,PPCDC |
| rs2304903 | 15 | 75315778 | COX5A,RPP25,SCAMP5,PPCDC |
| rs8042558 | 15 | 75320433 | COX5A,RPP25,SCAMP5,PPCDC |
| rs8028632 | 15 | 75321262 | COX5A,RPP25,SCAMP5,PPCDC |
| rs2120019 | 15 | 75334184 | RPP25,SCAMP5,PPCDC |
| rs8043088 | 15 | 81490320 | C15orf26,IL16 |
| rs12903128 | 15 | 81651651 | IL16,STARD5,TMC3 |
| rs7169250 | 15 | 81661639 | IL16,STARD5,TMC3 |
| rs8032164 | 15 | 81664087 | IL16,STARD5,TMC3 |
| rs1567085 | 15 | 84758698 | ADAMTSL3,EFTUD1P1,UBE2Q2L |
| rs11857939 | 15 | 92424655 | SLCO3A1 |
| rs10163001 | 15 | 94024458 |  |
| rs4777893 | 15 | 94024743 |  |
| rs6497080 | 15 | 94033802 |  |
| rs6600231 | 16 | 766485 | RAB40C,WFIKKN1,C16orf13,FAM195A,WDR90,RHOT2,RHBDL1,STUB1,JMJD8,WDR24,FBXL16,METRN,FAM173A,CCDC78,HAGHL,NARFL,MSLN,MIR662,RPUSD1,CHTF18,GNG13,PRR25 |
| rs442234 | 16 | 12072161 | GSPT1,TNFRSF17,SNX29 |
| rs7500910 | 16 | 12385491 | SNX29 |
| rs9937570 | 16 | 17262065 | XYLT1 |
| rs1542421 | 16 | 17270944 | XYLT1 |
| rs7190310 | 16 | 17271862 | XYLT1 |
| rs8047616 | 16 | 28967688 | SH2B1,ATP2A1,LOC100289092,RABEP2,CD19,NFATC2IP,MIR4517,SPNS1,LAT |
| rs8044999 | 16 | 28974658 | SH2B1,ATP2A1,LOC100289092,RABEP2,CD19,NFATC2IP,MIR4517,SPNS1,LAT |
| rs8060015 | 16 | 28982227 | SH2B1,ATP2A1,LOC100289092,RABEP2,CD19,NFATC2IP,MIR4517,SPNS1,LAT |
| rs7202093 | 16 | 28998949 | ATP2A1,RABEP2,CD19,NFATC2IP,MIR4517,SPNS1,LAT,RRN3P2 |
| rs7199660 | 16 | 50900311 | CYLD |
| rs1345390 | 16 | 54044515 | FTO,FTO-IT1 |
| rs860713 | 16 | 54069465 | FTO,FTO-IT1 |
| rs13333116 | 16 | 59224825 |  |
| rs13335924 | 16 | 59226850 |  |
| rs9934151 | 16 | 59302754 |  |
| rs17185893 | 16 | 59310833 |  |
| rs1117306 | 16 | 59321826 |  |
| rs9939608 | 16 | 59392020 |  |
| rs17258247 | 16 | 59394794 |  |
| rs11641340 | 16 | 78551936 | WWOX |
| rs9924445 | 16 | 86746818 |  |
| rs9921147 | 16 | 87475422 | FBXO31,MAP1LC3B,ZCCHC14,LOC101928737 |
| rs4465613 | 16 | 87892271 | KLHDC4,SLC7A5,MIR6775,CA5A,BANP |
| rs2286881 | 17 | 2267694 | SMG6,SRR,TSR1,SNORD91B,SNORD91A,SGSM2,MNT,LOC284009,METTL16 |
| rs8071333 | 17 | 2815724 | RAP1GAP2,LOC101927911 |
| rs17825515 | 17 | 5864270 | LOC339166 |
| rs729355 | 17 | 15349114 | CDRT4,TVP23C-CDRT4,TVP23C |
| rs3818717 | 17 | 17707105 | RAI1,SMCR5,SREBF1,MIR6777,MIR33B,TOM1L2 |
| rs11657423 | 17 | 17728574 | RAI1,SMCR5,SREBF1,MIR6777,MIR33B,TOM1L2 |
| rs9907246 | 17 | 17770965 | RAI1,SMCR5,SREBF1,MIR6777,MIR33B,TOM1L2 |
| rs950966 | 17 | 17783748 | RAI1,SREBF1,MIR6777,MIR33B,TOM1L2,LRRC48 |
| rs4925123 | 17 | 17784374 | RAI1,SREBF1,MIR6777,MIR33B,TOM1L2,LRRC48 |
| rs11650649 | 17 | 17790319 | RAI1,SREBF1,MIR6777,MIR33B,TOM1L2,LRRC48 |
| rs8070128 | 17 | 17804725 | RAI1,SREBF1,MIR6777,MIR33B,TOM1L2,LRRC48 |
| rs4925129 | 17 | 17807186 | RAI1,SREBF1,MIR6777,MIR33B,TOM1L2,LRRC48 |
| rs4925130 | 17 | 17807858 | RAI1,SREBF1,MIR6777,MIR33B,TOM1L2,LRRC48 |
| rs8078138 | 17 | 17811251 | RAI1,SREBF1,MIR6777,MIR33B,TOM1L2,LRRC48 |
| rs7222480 | 17 | 17818817 | SREBF1,TOM1L2,LRRC48 |
| rs5002487 | 17 | 17832460 | SREBF1,TOM1L2,LRRC48,ATPAF2 |
| rs8080823 | 17 | 17864048 | TOM1L2,LRRC48,ATPAF2,GID4 |
| rs2955382 | 17 | 17947710 | TOM1L2,LRRC48,ATPAF2,GID4,DRG2,MYO15A |
| rs4925138 | 17 | 17992793 | LRRC48,ATPAF2,GID4,DRG2,MYO15A,ALKBH5 |
| rs854813 | 17 | 18003845 | LRRC48,ATPAF2,GID4,DRG2,MYO15A,ALKBH5 |
| rs8064539 | 17 | 18903576 | PRPSAP2,SLC5A10,FAM83G,GRAP |
| rs7211874 | 17 | 18904830 | PRPSAP2,SLC5A10,FAM83G,GRAP |
| rs16966358 | 17 | 39029805 | KRT27,KRT28,KRT10,TMEM99,KRT12,KRT20,KRT23,KRT39 |
| rs634370 | 17 | 47287109 | B4GALNT2,GNGT2,ABI3,PHOSPHO1,FLJ40194,MIR6129,ZNF652 |
| rs8081200 | 17 | 59959765 | BRIP1,INTS2,MED13 |
| rs9910451 | 17 | 60097081 | INTS2,MED13 |
| rs3176975 | 17 | 64210757 | CEP112,APOH,PRKCA |
| rs4791075 | 17 | 64238065 | CEP112,APOH,PRKCA |
| rs9896077 | 17 | 64263585 | CEP112,APOH,PRKCA |
| rs6504406 | 17 | 64271724 | CEP112,APOH,PRKCA |
| rs9894043 | 17 | 64274133 | CEP112,APOH,PRKCA |
| rs12452592 | 17 | 70188525 | SOX9-AS1,LOC101928205,SOX9 |
| rs8094014 | 18 | 5940341 | MIR3976,TMEM200C,L3MBTL4 |
| rs8093868 | 18 | 27587686 |  |
| rs2850905 | 18 | 75019994 | GALR1 |
| rs10403842 | 19 | 14857891 | EMR3,ZNF333,EMR2,OR7C1,OR7A5,OR7A10 |
| rs999967 | 19 | 14863522 | EMR3,ZNF333,EMR2,OR7C1,OR7A5,OR7A10 |
| rs10417806 | 19 | 17488141 | BABAM1,ANKLE1,ABHD8,MRPL34,DDA1,ANO8,GTPBP3,PLVAP,BST2,MVB12A,TMEM221,NXNL1,SLC27A1 |
| rs12972060 | 19 | 33537096 | CEP89,C19orf40,RHPN2,GPATCH1,WDR88 |
| rs10411529 | 19 | 33616256 | RHPN2,GPATCH1,WDR88,LRP3,SLC7A10 |
| rs2599472 | 19 | 43951421 | PRG1,CD177,TEX101,LYPD3,PHLDB3,ETHE1,ZNF575,XRCC1 |
| rs157810 | 20 | 450704 | TRIB3,RBCK1,TBC1D20,CSNK2A1 |
| rs6081944 | 20 | 2078823 | STK35 |
| rs6079391 | 20 | 14306953 | MACROD2,FLRT3 |
| rs6079401 | 20 | 14346508 | MACROD2,FLRT3 |
| rs169226 | 20 | 22374540 | LOC284788 |
| rs1570841 | 20 | 34048161 | UQCC1,GDF5,CEP250,C20orf173,ERGIC3,FER1L4 |
| rs8116304 | 20 | 34054409 | UQCC1,GDF5,CEP250,C20orf173,ERGIC3,FER1L4 |
| rs1886696 | 20 | 34055706 | UQCC1,GDF5,CEP250,C20orf173,ERGIC3,FER1L4 |
| rs6060431 | 20 | 34059337 | UQCC1,GDF5,CEP250,C20orf173,ERGIC3,FER1L4 |
| rs2236165 | 20 | 34096725 | UQCC1,GDF5,CEP250,C20orf173,ERGIC3,FER1L4 |
| rs2236164 | 20 | 34097353 | UQCC1,GDF5,CEP250,C20orf173,ERGIC3,FER1L4 |
| rs2236160 | 20 | 34101821 | GDF5,CEP250,C20orf173,ERGIC3,FER1L4 |
| rs224415 | 20 | 34135629 | CEP250,C20orf173,ERGIC3,FER1L4,SPAG4,CPNE1 |
| rs224440 | 20 | 34159447 | CEP250,C20orf173,ERGIC3,FER1L4,SPAG4,CPNE1,RBM12,NFS1 |
| rs2585450 | 20 | 52747506 | BCAS1,MIR4756,CYP24A1,PFDN4 |
| rs2829803 | 21 | 26948310 | MIR155HG,MIR155,LINC00515,MRPL39,JAM2 |
| rs2329577 | 21 | 45227299 | PDXK,CSTB,RRP1,LOC284837,AGPAT3 |
| rs564493 | 22 | 20977077 | MED15,POM121L4P,TMEM191A,PI4KA |
| rs3966269 | 22 | 24867882 | SPECC1L,SPECC1L-ADORA2A,ADORA2A,ADORA2A-AS1,UPB1,GUCD1,SNRPD3 |
| rs4821475 | 22 | 36669095 | APOL4,APOL2,APOL1,MYH9,MIR6819 |
| rs2016013 | 22 | 45121864 | PRR5,PRR5-ARHGAP8,ARHGAP8 |
| rs5770881 | 22 | 50985269 | SBF1,ADM2,MIOX,LMF2,NCAPH2,SCO2,TYMP,ODF3B,KLHDC7B,SYCE3,CPT1B,CHKB-CPT1B,CHKB,CHKB-AS1,MAPK8IP2,ARSA |
| rs3915207 | 23 | 4315661 |  |
| rs5935245 | 23 | 12162126 | FRMPD4 |
| rs1476595 | 23 | 13255459 | GS1-600G8.3,ATXN3L,LINC01203 |
| rs5979840 | 23 | 13263172 | GS1-600G8.3,ATXN3L,LINC01203 |
| rs2214203 | 23 | 13912740 | GPM6B |
| rs17300793 | 23 | 13930034 | GPM6B,GEMIN8 |
| rs11798108 | 23 | 13931785 | GPM6B,GEMIN8 |
| rs12011117 | 23 | 15800405 | CA5BP1,CA5B,INE2,ZRSR2,AP1S2 |
| rs6527642 | 23 | 15810604 | CA5BP1,CA5B,INE2,ZRSR2,AP1S2 |
| rs434380 | 23 | 18841271 | PPEF1,PHKA2-AS1,PHKA2 |
| rs2283712 | 23 | 18842784 | PPEF1,PHKA2-AS1,PHKA2 |
| rs6629323 | 23 | 19217859 | GPR64 |
| rs2363130 | 23 | 23367895 | PTCHD1-AS,PTCHD1 |
| rs2707160 | 23 | 23923684 | APOO,CXorf58,KLHL15 |
| rs2520229 | 23 | 23939679 | APOO,CXorf58,KLHL15 |
| rs3813165 | 23 | 24005086 | APOO,CXorf58,KLHL15,EIF2S3 |
| rs5970824 | 23 | 24041718 | CXorf58,KLHL15,EIF2S3 |
| rs11094949 | 23 | 24104646 | KLHL15,EIF2S3,ZFX-AS1,ZFX |
| rs7877712 | 23 | 24145844 | EIF2S3,ZFX-AS1,ZFX |
| rs5949232 | 23 | 24163287 | EIF2S3,ZFX-AS1,ZFX |
| rs2040577 | 23 | 24257499 | ZFX-AS1,ZFX,SUPT20HL2 |
| rs2040576 | 23 | 24260078 | ZFX-AS1,ZFX,SUPT20HL2 |
| rs5949263 | 23 | 24298077 | ZFX,SUPT20HL2,SUPT20HL1 |
| rs1947879 | 23 | 25218185 |  |
| rs6526422 | 23 | 25220716 |  |
| rs5986763 | 23 | 25261728 |  |
| rs5986769 | 23 | 25276181 |  |
| rs1481161 | 23 | 25301167 |  |
| rs6630033 | 23 | 25458023 |  |
| rs5944148 | 23 | 25776639 |  |
| rs5944298 | 23 | 26119617 | MAGEB18,MAGEB6 |
| rs5944301 | 23 | 26124826 | MAGEB18,MAGEB6 |
| rs5944314 | 23 | 26150157 | MAGEB18,MAGEB6,MAGEB5 |
| rs1014723 | 23 | 29419670 | IL1RAPL1 |
| rs1014724 | 23 | 29419910 | IL1RAPL1 |
| rs2079521 | 23 | 30320215 | MAGEB2,MAGEB3,MAGEB4,MAGEB1,NR0B1 |
| rs5928556 | 23 | 34447654 |  |
| rs4272535 | 23 | 34492370 |  |
| rs5971869 | 23 | 34504413 |  |
| rs6629027 | 23 | 36007614 | CXorf22,LOC101928564,CHDC2 |
| rs6632458 | 23 | 36031808 | CXorf22,LOC101928564,CHDC2 |
| rs6632464 | 23 | 36057261 | CXorf22,LOC101928564,CHDC2 |
| rs6632489 | 23 | 36124071 | CHDC2 |
| rs5972036 | 23 | 36298319 | CXorf30,RP11-87M18.2 |
| rs17273931 | 23 | 37905114 | CXorf27,SYTL5 |
| rs206047 | 23 | 41973439 |  |
| rs5991678 | 23 | 43396447 |  |
| rs3810709 | 23 | 43587868 | MAOA,MAOB |
| rs6610846 | 23 | 43589464 | MAOA,MAOB |
| rs5905859 | 23 | 43591500 | MAOA,MAOB |
| rs7065889 | 23 | 44434260 | FUNDC1 |
| rs2745708 | 23 | 45610875 | LOC392452,MIR221,MIR222 |
| rs5905485 | 23 | 45681372 | LOC392452,MIR221,MIR222 |
| rs5905489 | 23 | 45722990 |  |
| rs6521055 | 23 | 45734847 |  |
| rs5906201 | 23 | 46285456 | LINC01186,KRBOX4,ZNF674 |
| rs5936657 | 23 | 68347670 | PJA1,LINC00269 |
| rs6525273 | 23 | 68372827 | PJA1,LINC00269 |
| rs6525292 | 23 | 68534053 |  |
| rs17302855 | 23 | 68543004 |  |
| rs7880460 | 23 | 68546101 |  |
| rs5937091 | 23 | 70745323 | BCYRN1,TAF1,INGX,OGT,ACRC,CXCR3 |
| rs12010107 | 23 | 71503284 | PIN4,ERCC6L,RPS4X,CITED1,HDAC8 |
| rs2984344 | 23 | 71523614 | PIN4,ERCC6L,RPS4X,CITED1,HDAC8 |
| rs3012658 | 23 | 71566745 | PIN4,RPS4X,CITED1,HDAC8 |
| rs5937900 | 23 | 74027649 | KIAA2022 |
| rs6647073 | 23 | 74089464 | KIAA2022 |
| rs6647074 | 23 | 74100589 | KIAA2022 |
| rs1751108 | 23 | 78596731 | ITM2A |
| rs5913235 | 23 | 79479057 | CHMP1B2P |
| rs4826173 | 23 | 79509262 | CHMP1B2P,FAM46D |
| rs5912420 | 23 | 79528472 | CHMP1B2P,FAM46D |
| rs5913245 | 23 | 79537126 | CHMP1B2P,FAM46D |
| rs1927060 | 23 | 79539295 | CHMP1B2P,FAM46D |
| rs5912421 | 23 | 79555020 | CHMP1B2P,FAM46D |
| rs4497095 | 23 | 79567952 | CHMP1B2P,FAM46D |
| rs2088057 | 23 | 79651712 | CHMP1B2P,FAM46D |
| rs2063579 | 23 | 79925246 | BRWD3 |
| rs3106407 | 23 | 79994465 | BRWD3 |
| rs3923574 | 23 | 80000302 | BRWD3 |
| rs3123257 | 23 | 80036065 | BRWD3 |
| rs4567187 | 23 | 80058167 | BRWD3 |
| rs242839 | 23 | 85470416 | DACH2 |
| rs5923516 | 23 | 85489261 | DACH2 |
| rs1039890 | 23 | 85491168 | DACH2 |
| rs6524604 | 23 | 85493745 | DACH2 |
| rs1606012 | 23 | 85496068 | DACH2 |
| rs958350 | 23 | 85498835 | DACH2 |
| rs5967693 | 23 | 85504672 | DACH2 |
| rs7061659 | 23 | 85506190 | DACH2 |
| rs1848905 | 23 | 85506917 | DACH2 |
| rs5968843 | 23 | 85508456 | DACH2 |
| rs6618944 | 23 | 91538648 | PCDH11X |
| rs6522742 | 23 | 93326808 |  |
| rs5966608 | 23 | 97742503 |  |
| rs7059785 | 23 | 97748817 |  |
| rs11798552 | 23 | 97763035 |  |
| rs5921138 | 23 | 97833775 |  |
| rs5920672 | 23 | 97833811 |  |
| rs2185737 | 23 | 97845602 |  |
| rs3827415 | 23 | 99904216 | TNMD,TSPAN6,SRPX2,SYTL4 |
| rs5921620 | 23 | 99919476 | TNMD,TSPAN6,SRPX2,SYTL4 |
| rs5951354 | 23 | 100813196 | ARMCX4,ARMCX1,ARMCX6,ARMCX3,ARMCX2 |
| rs5951355 | 23 | 100813327 | ARMCX4,ARMCX1,ARMCX6,ARMCX3,ARMCX2 |
| rs7878331 | 23 | 102175437 | LINC00630,RAB40AL |
| rs6523686 | 23 | 102179286 | LINC00630,RAB40AL |
| rs5987542 | 23 | 102798895 | RAB40A,TCEAL4,TCEAL3,TCEAL1 |
| rs5985319 | 23 | 108271926 |  |
| rs6642856 | 23 | 108284637 |  |
| rs6642481 | 23 | 108286494 |  |
| rs4893489 | 23 | 108287984 |  |
| rs4243373 | 23 | 108288299 |  |
| rs5985603 | 23 | 108289150 |  |
| rs5942729 | 23 | 108294623 |  |
| rs5942733 | 23 | 108297180 |  |
| rs4893410 | 23 | 108316376 |  |
| rs5985628 | 23 | 108316921 |  |
| rs5942752 | 23 | 108322872 |  |
| rs6413651 | 23 | 108332533 |  |
| rs4893413 | 23 | 108339383 |  |
| rs5985679 | 23 | 108363694 |  |
| rs6643013 | 23 | 108400107 |  |
| rs6642519 | 23 | 108402452 |  |
| rs1857536 | 23 | 108406338 |  |
| rs1936492 | 23 | 108414092 |  |
| rs6643039 | 23 | 108417457 |  |
| rs6643045 | 23 | 108421100 |  |
| rs6642615 | 23 | 108513405 |  |
| rs1205524 | 23 | 108544388 | GUCY2F |
| rs471900 | 23 | 108545876 | GUCY2F |
| rs504284 | 23 | 108547100 | GUCY2F |
| rs669418 | 23 | 108563970 | GUCY2F |
| rs668981 | 23 | 108564053 | GUCY2F |
| rs618821 | 23 | 108580639 | GUCY2F |
| rs2179585 | 23 | 108587885 | GUCY2F |
| rs2473638 | 23 | 108593683 | GUCY2F |
| rs2480514 | 23 | 108594053 | GUCY2F |
| rs5987956 | 23 | 114871655 | PLS3-AS1,PLS3 |
| rs1859673 | 23 | 116499173 |  |
| rs2335752 | 23 | 116506100 |  |
| rs1558022 | 23 | 116508319 |  |
| rs6646846 | 23 | 116512782 |  |
| rs5956581 | 23 | 116515049 |  |
| rs2040959 | 23 | 116587448 |  |
| rs5911899 | 23 | 116595019 |  |
| rs6645775 | 23 | 116618312 |  |
| rs4825546 | 23 | 116950556 | KLHL13 |
| rs4825695 | 23 | 119254837 | RHOXF2B,RHOXF2,RHOXF1 |
| rs6608068 | 23 | 122425522 | GRIA3 |
| rs1781115 | 23 | 123320332 | STAG2 |
| rs7888274 | 23 | 123322590 | STAG2 |
| rs1781105 | 23 | 123322741 | STAG2 |
| rs2858443 | 23 | 123690015 | TENM1 |
| rs2843525 | 23 | 123696648 | TENM1 |
| rs2858405 | 23 | 123716255 | TENM1 |
| rs156688 | 23 | 126346566 |  |
| rs11095691 | 23 | 136391940 |  |
| rs5976361 | 23 | 138469749 | SRD5A1P1 |
| rs3848924 | 23 | 138486733 | SRD5A1P1 |
| rs2367651 | 23 | 138486815 | SRD5A1P1 |
| rs3859961 | 23 | 138487372 | SRD5A1P1 |
| rs3903643 | 23 | 138487531 | SRD5A1P1 |
| rs3903645 | 23 | 138487695 | SRD5A1P1 |
| rs4083445 | 23 | 138488800 | SRD5A1P1 |
| rs5908107 | 23 | 141136519 |  |
| rs859905 | 23 | 143413997 |  |
| rs5919644 | 23 | 145234818 |  |
| rs2233048 | 23 | 148797348 | TMEM185A,MAGEA11,HSFX1,HSFX2,MAGEA9B,MAGEA9 |
| rs7879285 | 23 | 150847294 | PASD1,PRRG3,FATE1,CNGA2 |
| rs6571298 | 23 | 152704232 | ZNF275,ZFP92,TREX2,HAUS7,BGN,ATP2B3 |
| rs762734 | 23 | 152705317 | ZNF275,ZFP92,TREX2,HAUS7,BGN,ATP2B3 |
| rs5987170 | 23 | 152706348 | ZNF275,ZFP92,TREX2,HAUS7,BGN,ATP2B3 |
| rs4646263 | 23 | 153140449 | PLXNB3,SRPK3,IDH3G,SSR4,PDZD4,L1CAM,AVPR2,ARHGAP4,NAA10,RENBP,HCFC1,HCFC1-AS1,TMEM187 |
| rs7350355 | 23 | 153247745 | L1CAM,AVPR2,ARHGAP4,NAA10,RENBP,HCFC1,HCFC1-AS1,TMEM187,MIR3202-1,MIR3202-2,IRAK1,MIR718,MECP2 |
| rs5945384 | 23 | 153260414 | AVPR2,ARHGAP4,NAA10,RENBP,HCFC1,HCFC1-AS1,TMEM187,MIR3202-1,MIR3202-2,IRAK1,MIR718,MECP2 |
| rs2239464 | 23 | 153348431 | TMEM187,IRAK1,MIR718,MECP2,OPN1LW,OPN1MW,OPN1MW2 |
| rs5945431 | 23 | 153701716 | FLNA,EMD,RPL10,SNORA70,DNASE1L1,TAZ,LOC158960,ATP6AP1,GDI1,FAM50A,MIR6858,PLXNA3,LAGE3,UBL4A,SLC10A3,FAM3A,G6PD,IKBKG,FAM223B,FAM223A |
