# Supplementary material for: Evolutionary triangulation: informing genetic association studies with evolutionary evidence
Source: BioData Min. 2016 Apr 2;9:12. doi: 10.1186/s13040-016-0091-7 (PMC4818851; doi:10.1186/s13040-016-0091-7)
Supplement: Additional file 7: — Distribution of recombination hotspots surrounding ET SNP rs28117 near melanoma associated gene SLC45A2. (PDF 15 kb) [file 13040_2016_91_MOESM7_ESM.pdf]

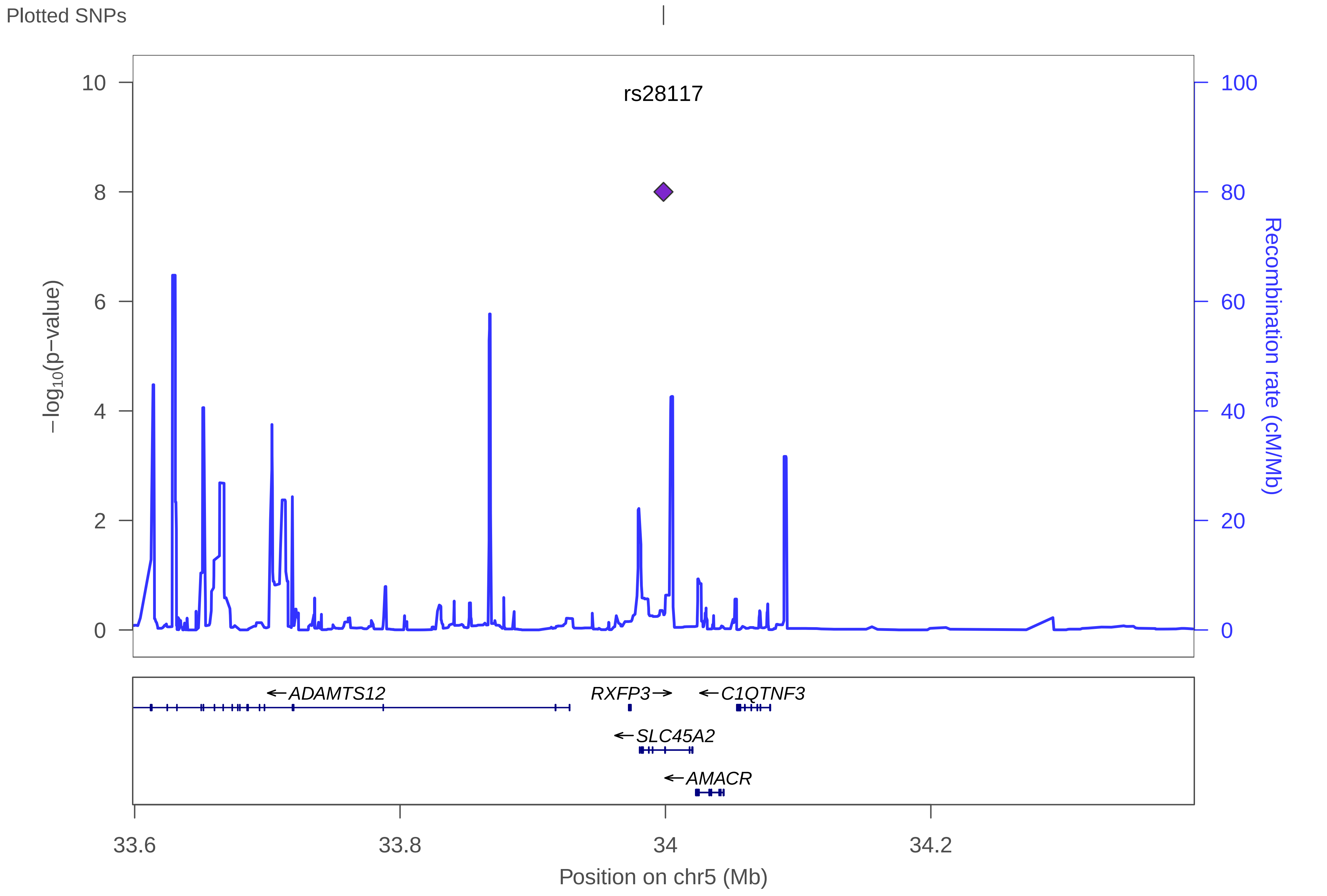

Plotted SNPs
rs28117
10
8
6
4
2
0
−log10(p−value)
100
80
60
40
20
0
Recombination rate (cM/Mb)
ADAMTS12
RXFP3
C1QTNF3
SLC45A2
AMACR
33.6
33.8
34
34.2
Position on chr5 (Mb)

date:	Sun Dec 6 18:26:08 2015
build:	hg18
display range:	chr5:33598527−34398527 [33598527−34398527]
hilite range:	0 − 0 [0 − 0]
reference SNP:	chr5:33998527
number of SNPs plotted:	1
max P−Value:	1E−8 [chr5:33998527]
min P−Value:	1E−8 [chr5:33998527]
Warning:	No usable LD information for reference SNP.

Make more plots at http://csg.sph.umich.edu/locuszoom/
