# Supplementary material for: Evolutionary triangulation: informing genetic association studies with evolutionary evidence
Source: BioData Min. 2016 Apr 2;9:12. doi: 10.1186/s13040-016-0091-7 (PMC4818851; doi:10.1186/s13040-016-0091-7)
Supplement: Additional file 8: Table S6. — Genetic associations of 85th/15th cutoff ET genes with diseases other than index diseases based on CEU-YRI-GIH comparisons1. (DOCX 145 kb) [file 13040_2016_91_MOESM8_ESM.docx]

**Table S6. Genetic associations of 85^th^/15^th^ cutoff ET genes with diseases other than index diseases based on CEU-YRI-GIH comparisons^1^**

| Disease | Associating ET Genes | 5^th 2^ | 10^th 2^ | Odds Ratio^3^ |
| --- | --- | --- | --- | --- |
| Albinism, Oculocutaneous | *OCA2* |  |  | Mendelian |
|  | *SLC45A2* | Y | Y | Mendelian |
| Alpha-Thalassemia | *G6PD* |  |  | Mendelian |
| Alzheimer Disease | *CTNNA3* |  |  | 1.18 [[1](#_ENREF_1)] |
|  | *GAB2* |  | Y | 4.06 [[2](#_ENREF_2)] |
|  | *GOLM1* |  |  | 1.40 [[3](#_ENREF_3)] |
|  | *IDE* |  |  | 2.72 [[4](#_ENREF_4)] |
|  | *IL6* |  | Y | 3.30 [[5](#_ENREF_5)] |
|  | *MAOA* |  |  | 2.79 [[6](#_ENREF_6)] |
|  | *PLAU* |  |  | 1.89 [[7](#_ENREF_7)] |
|  | *SORCS1* |  |  | 0.42 [[8](#_ENREF_8)] |
| Anemia, Sickle Cell | *G6PD* |  |  | Mendelian |
| Antiphospholipid Syndrome | *APOH* |  |  | 2.817 [[9](#_ENREF_9)] |
| Arthritis, Juvenile Rheumatoid/Arthritis, Rheumatoid | *IL6* |  | Y | 0.33 [[10](#_ENREF_10)] |
|  | *REL* |  |  | 1.21 [[11](#_ENREF_11)] |
| Asthma | *IFNG* |  |  | 0.50 [[12](#_ENREF_12)] |
|  | *IL6* |  | Y | 3.20 [[13](#_ENREF_13)] |
|  | *TLR10* |  |  | 0.58 [[14](#_ENREF_14)] |
|  | *TSLP* |  |  | 2.00 [[15](#_ENREF_15)] |
| Atrial Fibrillation | *IL6* |  | Y | 4.74 [[16](#_ENREF_16)] |
| Autistic Disorder | *MAOA* |  |  | 2.25 [[17](#_ENREF_17)] |
|  | *MECP2* |  | Y | P = 0.001 [[18](#_ENREF_18)] |
| Brain Ischemia/Stroke | *IL6* |  | Y | 8.60 [[19](#_ENREF_19)] |
| Brain Neoplasms/ Glioma | *XRCC1* |  |  | 3.18 [[20](#_ENREF_20)] |
| Breast Neoplasms | *ADH1C* |  |  | 1.5 [[21](#_ENREF_21)] |
|  | *BRIP1* |  |  | 2.00 [[22](#_ENREF_22)] |
|  | *CASP8* |  | Y | 1.74 [[23](#_ENREF_23)] |
|  | *CYP1A1* |  | Y | 8.07 [[24](#_ENREF_24)] |
|  | *CYP1A2* |  | Y | 2.75 [[25](#_ENREF_25)] |
|  | *FGFR2* |  |  | 3.97 [[26](#_ENREF_26)] |
|  | *IFNG* |  |  | 2.03 [[27](#_ENREF_27)] |
|  | *IL6* |  | Y | 4.40 [[28](#_ENREF_28)] |
|  | *XRCC1* |  |  | 8.74 [[29](#_ENREF_29)] |
| Brucellosis | *IFNG* |  |  | 3.45 [[30](#_ENREF_30)] |
| Celiac Disease | *LPP* |  |  | 2.53 [[31](#_ENREF_31)] |
| Colitis, Ulcerative/Crohn Disease/Inflammatory Bowel Diseases | *SLC22A4* |  |  | 7.50 [[32](#_ENREF_32)] |
|  | *SLC22A5* |  |  | 7.50 [[32](#_ENREF_32)] |
|  | *IL6* |  | Y | 6.10 [[33](#_ENREF_33)] |
| Colorectal Neoplasms/Rectal Neoplasms/Colonic Neoplasms | *ADH1B* |  |  | 3.44 [[34](#_ENREF_34)] |
|  | *ADH1C* |  |  | 2.32 [[35](#_ENREF_35)] |
|  | *CYP1A1* |  | Y | 9.14 [[36](#_ENREF_36)] |
|  | *CYP1A2* |  | Y | 6.16 [[36](#_ENREF_36)] |
|  | *IL6* |  | Y | 1.97 [[37](#_ENREF_37)] |
|  | *RHPN2* |  |  | 1.15 [[38](#_ENREF_38)] |
|  | *XRCC1* |  |  | 10.5 [[39](#_ENREF_39)] |
| Diabetes Mellitus, Type 1 | *IL6* |  | Y | 3.36 [[40](#_ENREF_40)] |
| Endometrial Neoplasms | *CYP1A1* |  | Y | 4.58 [[41](#_ENREF_41)] |
|  | *XRCC1* |  |  | 4.41 [[42](#_ENREF_42)] |
| Glucosephosphate Dehydrogenase Deficiency | *G6PD* |  |  | Mendelian |
| Hepatitis B/Hepatitis B, Chronic | *IFNG* |  |  | P= 0.003 [[43](#_ENREF_43)] |
| Kidney DiseasesKidney/Failure, Chronic | *APOL1* |  |  | 7.30 [[44](#_ENREF_44)] |
|  | *MYH9* |  |  | 4.59 [[45](#_ENREF_45)] |
| Leukemia/Leukemia, Myeloid/Leukemia, Lymphocytic, Acute | *CYP1A1* |  | Y | 0.054 [[46](#_ENREF_46)] |
| Lung Neoplasms/Carcinoma, Non-Small-Cell Lung | *ALK* |  |  | *p* = 0.0018 [[47](#_ENREF_47)] |
|  | *CASP8* |  | Y | 4.62 [[48](#_ENREF_48)] |
|  | *CYP1A1* |  | Y | 4.85 [[49](#_ENREF_49)] |
|  | *CYP1A2* |  | Y | 6.02 [[50](#_ENREF_50)] |
|  | *IL6* |  | Y | 1.91 [[51](#_ENREF_51)] |
|  | *XRCC1* |  |  | 5.75 [[52](#_ENREF_52)] |
| Lupus Erythematosus, Systemic | *IFNG* |  |  | 2.27 [[53](#_ENREF_53)] |
|  | *IL6* |  | Y | 1.98 [[54](#_ENREF_54)] |
|  | *IRAK1* |  | Y | 1.44 [[55](#_ENREF_55)] |
|  | *MECP2* |  | Y | 1.43 [[56](#_ENREF_56)] |
| Malaria/Malaria, Falciparum | *G6PD* |  |  | Mendelian |
| Multiple Sclerosis | *IL6* |  | Y | 2.88 [[57](#_ENREF_57)] |
| Obstetric Labor, Premature/Premature Birth | *IL6* |  | Y | 0.68 [[58](#_ENREF_58)] |
|  | *CYP1A1* |  | Y | 15.6 [[59](#_ENREF_59)] |
| Opioid-Related Disorders | *OPRM1* |  |  | 0.13 [[60](#_ENREF_60)] |
| Ovarian Neoplasms | *BRIP1* |  |  | 8.13 [[61](#_ENREF_61)] |
|  | *CYP1A1* |  | Y | 8.77 [[62](#_ENREF_62)] |
|  | *CYP1A2* |  | Y | 0.34 [[63](#_ENREF_63)] |
| Pain Threshold | *OPRM1* |  |  | *p* < 0.05 [[64](#_ENREF_64)] |
| Panic Disorder | *MAOA* |  |  | 1.23 [[65](#_ENREF_65)] |
| Parkinson Disease | *MAOB* |  |  | 2.07 [[66](#_ENREF_66)] |
| Prostatic Neoplasms | *AMACR* | Y | Y | 0.47 [[67](#_ENREF_67)] |
|  | *CYP1A1* |  | Y | 2.38 [[68](#_ENREF_68)] |
|  | *IL6* |  | Y | 2.46 [[69](#_ENREF_69)] |
|  | *ITGA6* |  |  | 1.33 [[70](#_ENREF_70)] |
|  | *XRCC1* |  |  | 4.31 [[71](#_ENREF_71)] |
| Pulmonary Disease, Chronic Obstructive | *FAM13A* |  | Y | 0.76 [[72](#_ENREF_72)] |
|  | *SOD3* |  |  | 2.23 [[73](#_ENREF_73)] |
| Smith-Lemli-Opitz Syndrome | *DHCR7* | Y | Y | Mendelian |
| Tuberculosis/Tuberculosis, Pulmonary | *IFNG* |  |  | 3.75 [[74](#_ENREF_74)] |
| Urinary Bladder Neoplasms | *CYP1A1* |  | Y | 1.23 [[75](#_ENREF_75)] |
|  | *XRCC1* |  |  | 2.99 [[76](#_ENREF_76)] |
| Uterine Cervical Neoplasms/Uterine Neoplasms | *IFNG* |  |  | 3.30 [[77](#_ENREF_77)] |
|  | *XRCC1* |  |  | 4.13 [[78](#_ENREF_78)] |
|  | *CYP1A1* |  | Y | 11.3 [[79](#_ENREF_79)] |
| Vitamin D Deficiency | *CYP24A1* |  |  | 2.47 [[80](#_ENREF_80)] |
|  | *DHCR7* | Y | Y | 2.47 [[80](#_ENREF_80)] |

^1^ Other diseases also having the same prevalence distribution among selected populations as that of index diseases

^2^ 5^th^/10^th^: Y indicates ET genes identified under the 95^th^/5^th^ or the 90^th^/10^th^ cutoff

^3^ Largest reported Odds ratio or smallest p value.

**References**

1. Morgan, A.R., et al., *Association analysis of 528 intra-genic SNPs in a region of chromosome 10 linked to late onset Alzheimer's disease.* Am J Med Genet B Neuropsychiatr Genet, 2008. **147B**(6): p. 727-31.

2. Reiman, E.M., et al., *GAB2 alleles modify Alzheimer's risk in APOE epsilon4 carriers.* Neuron, 2007. **54**(5): p. 713-20.

3. Yuan, Q., C. Chu, and J. Jia, *Association studies of 19 candidate SNPs with sporadic Alzheimer's disease in the North Chinese Han population.* Neurol Sci, 2012. **33**(5): p. 1021-8.

4. Cui, P.J., et al., *The association between two single nucleotide polymorphisms within the insulin-degrading enzyme gene and Alzheimer's disease in a Chinese Han population.* J Clin Neurosci, 2012. **19**(5): p. 745-9.

5. Wang, M. and J. Jia, *The interleukin-6 gene -572C/G promoter polymorphism modifies Alzheimer's risk in APOE epsilon 4 carriers.* Neurosci Lett, 2010. **482**(3): p. 260-3.

6. Takehashi, M., et al., *Association of monoamine oxidase A gene polymorphism with Alzheimer's disease and Lewy body variant.* Neurosci Lett, 2002. **327**(2): p. 79-82.

7. Finckh, U., et al., *Association of late-onset Alzheimer disease with a genotype of PLAU, the gene encoding urokinase-type plasminogen activator on chromosome 10q22.2.* Neurogenetics, 2003. **4**(4): p. 213-7.

8. Wang, H.F., et al., *SORCS1 and APOE polymorphisms interact to confer risk for late-onset Alzheimer's disease in a Northern Han Chinese population.* Brain Res, 2012. **1448**: p. 111-6.

9. Lee, Y.H., et al., *Association between the valine/leucine247 polymorphism of beta2-glycoprotein I and susceptibility to anti-phospholipid syndrome: a meta-analysis.* Lupus, 2012. **21**(8): p. 865-71.

10. Lee, Y.H., et al., *The association between interleukin-6 polymorphisms and rheumatoid arthritis: a meta-analysis.* Inflamm Res, 2012. **61**(7): p. 665-71.

11. Gregersen, P.K., et al., *REL, encoding a member of the NF-kappaB family of transcription factors, is a newly defined risk locus for rheumatoid arthritis.* Nat Genet, 2009. **41**(7): p. 820-3.

12. Kumar, A. and B. Ghosh, *A single nucleotide polymorphism (A --&gt; G) in intron 3 of IFNgamma gene is associated with asthma.* Genes Immun, 2008. **9**(4): p. 294-301.

13. Settin, A., et al., *Gene polymorphisms of IL-6(-174) G/C and IL-1Ra VNTR in asthmatic children.* Indian J Pediatr, 2008. **75**(10): p. 1019-23.

14. Kormann, M.S., et al., *Toll-like receptor heterodimer variants protect from childhood asthma.* J Allergy Clin Immunol, 2008. **122**(1): p. 86-92, 92 e1-8.

15. Liu, M., et al., *Genetic variants of TSLP and asthma in an admixed urban population.* PLoS One, 2011. **6**(9): p. e25099.

16. Li, J., et al., *Interleukin-6 promoter polymorphisms and susceptibility to atrial fibrillation in elderly Han Chinese patients with essential hypertension.* J Interferon Cytokine Res, 2012. **32**(11): p. 542-7.

17. Salem, A.M., et al., *Genetic variants of neurotransmitter-related genes and miRNAs in Egyptian autistic patients.* ScientificWorldJournal, 2013. **2013**: p. 670621.

18. Loat, C.S., et al., *Methyl-CpG-binding protein 2 polymorphisms and vulnerability to autism.* Genes Brain Behav, 2008. **7**(7): p. 754-60.

19. Pola, R., et al., *Synergistic effect of -174 G/C polymorphism of the interleukin-6 gene promoter and 469 E/K polymorphism of the intercellular adhesion molecule-1 gene in Italian patients with history of ischemic stroke.* Stroke, 2003. **34**(4): p. 881-5.

20. Kiuru, A., et al., *XRCC1 and XRCC3 variants and risk of glioma and meningioma.* J Neurooncol, 2008. **88**(2): p. 135-42.

21. Terry, M.B., et al., *ADH3 genotype, alcohol intake and breast cancer risk.* Carcinogenesis, 2006. **27**(4): p. 840-7.

22. Seal, S., et al., *Truncating mutations in the Fanconi anemia J gene BRIP1 are low-penetrance breast cancer susceptibility alleles.* Nat Genet, 2006. **38**(11): p. 1239-41.

23. Catucci, I., et al., *The CASP8 rs3834129 polymorphism and breast cancer risk in BRCA1 mutation carriers.* Breast Cancer Res Treat, 2011. **125**(3): p. 855-60.

24. Shen, Y., et al., *Joint effects of the CYP1A1 MspI, ERalpha PvuII, and ERalpha XbaI polymorphisms on the risk of breast cancer: results from a population-based case-control study in Shanghai, China.* Cancer Epidemiol Biomarkers Prev, 2006. **15**(2): p. 342-7.

25. Sangrajrang, S., et al., *Genetic polymorphisms of estrogen metabolizing enzyme and breast cancer risk in Thai women.* Int J Cancer, 2009. **125**(4): p. 837-43.

26. Murillo-Zamora, E., et al., *Association between rs2981582 polymorphism in the FGFR2 gene and the risk of breast cancer in Mexican women.* Arch Med Res, 2013. **44**(6): p. 459-66.

27. Kamali-Sarvestani, E., A. Merat, and A.R. Talei, *Polymorphism in the genes of alpha and beta tumor necrosis factors (TNF-alpha and TNF-beta) and gamma interferon (IFN-gamma) among Iranian women with breast cancer.* Cancer Lett, 2005. **223**(1): p. 113-9.

28. Slattery, M.L., et al., *Active and passive smoking, IL6, ESR1, and breast cancer risk.* Breast Cancer Res Treat, 2008. **109**(1): p. 101-11.

29. Smith, T.R., et al., *Polymorphisms of XRCC1 and XRCC3 genes and susceptibility to breast cancer.* Cancer Lett, 2003. **190**(2): p. 183-90.

30. Hedayatizadeh-Omran, A., et al., *Interferon-gamma low producer genotype +5644 over presented in patients with focal brucellosis.* Pak J Biol Sci, 2010. **13**(21): p. 1036-41.

31. Izzo, V., et al., *Improving the estimation of celiac disease sibling risk by non-HLA genes.* PLoS One, 2011. **6**(11): p. e26920.

32. Newman, B., et al., *A risk haplotype in the Solute Carrier Family 22A4/22A5 gene cluster influences phenotypic expression of Crohn's disease.* Gastroenterology, 2005. **128**(2): p. 260-9.

33. Guerreiro, C.S., et al., *Fatty acids, IL6, and TNFalpha polymorphisms: an example of nutrigenetics in Crohn's disease.* Am J Gastroenterol, 2009. **104**(9): p. 2241-9.

34. Gao, C.M., et al., *Polymorphisms of alcohol dehydrogenase 2 and aldehyde dehydrogenase 2 and colorectal cancer risk in Chinese males.* World J Gastroenterol, 2008. **14**(32): p. 5078-83.

35. Bongaerts, B.W., et al., *Alcohol consumption, alcohol dehydrogenase 1C (ADH1C) genotype, and risk of colorectal cancer in the Netherlands Cohort Study on diet and cancer.* Alcohol, 2011. **45**(3): p. 217-25.

36. Yoshida, K., et al., *Association of CYP1A1, CYP1A2, GSTM1 and NAT2 gene polymorphisms with colorectal cancer and smoking.* Asian Pac J Cancer Prev, 2007. **8**(3): p. 438-44.

37. Yu, Y., et al., *IL6 gene polymorphisms and susceptibility to colorectal cancer: a meta-analysis and review.* Mol Biol Rep, 2012. **39**(8): p. 8457-63.

38. Houlston, R.S., et al., *Meta-analysis of genome-wide association data identifies four new susceptibility loci for colorectal cancer.* Nat Genet, 2008. **40**(12): p. 1426-35.

39. Krupa, R. and J. Blasiak, *An association of polymorphism of DNA repair genes XRCC1 and XRCC3 with colorectal cancer.* J Exp Clin Cancer Res, 2004. **23**(2): p. 285-94.

40. Settin, A., et al., *Gene polymorphisms of TNF-alpha-308 (G/A), IL-10(-1082) (G/A), IL-6(-174) (G/C) and IL-1Ra (VNTR) in Egyptian cases with type 1 diabetes mellitus.* Autoimmunity, 2009. **42**(1): p. 50-5.

41. Hirata, H., et al., *CYP1A1, SULT1A1, and SULT1E1 polymorphisms are risk factors for endometrial cancer susceptibility.* Cancer, 2008. **112**(9): p. 1964-73.

42. Sobczuk, A., T. Poplawski, and J. Blasiak, *Polymorphisms of DNA repair genes in endometrial cancer.* Pathol Oncol Res, 2012. **18**(4): p. 1015-20.

43. Ben-Ari, Z., et al., *Cytokine gene polymorphisms in patients infected with hepatitis B virus.* Am J Gastroenterol, 2003. **98**(1): p. 144-50.

44. Genovese, G., et al., *Association of trypanolytic ApoL1 variants with kidney disease in African Americans.* Science, 2010. **329**(5993): p. 841-5.

45. Behar, D.M., et al., *African ancestry allelic variation at the MYH9 gene contributes to increased susceptibility to non-diabetic end-stage kidney disease in Hispanic Americans.* Hum Mol Genet, 2010. **19**(9): p. 1816-27.

46. Bolufer, P., et al., *Profile of polymorphisms of drug-metabolising enzymes and the risk of therapy-related leukaemia.* Br J Haematol, 2007. **136**(4): p. 590-6.

47. Zhang, X., et al., *Fusion of EML4 and ALK is associated with development of lung adenocarcinomas lacking EGFR and KRAS mutations and is correlated with ALK expression.* Mol Cancer, 2010. **9**: p. 188.

48. Hart, K., et al., *A combination of functional polymorphisms in the CASP8, MMP1, IL10 and SEPS1 genes affects risk of non-small cell lung cancer.* Lung Cancer, 2011. **71**(2): p. 123-9.

49. Hung, R.J., et al., *CYP1A1 and GSTM1 genetic polymorphisms and lung cancer risk in Caucasian non-smokers: a pooled analysis.* Carcinogenesis, 2003. **24**(5): p. 875-82.

50. B'Chir, F., et al., *CYP1A2 genetic polymorphisms and adenocarcinoma lung cancer risk in the Tunisian population.* Life Sci, 2009. **84**(21-22): p. 779-84.

51. Nie, W., et al., *Interleukin-6 -634C/G polymorphism is associated with lung cancer risk: a meta-analysis.* Tumour Biol, 2014. **35**(5): p. 4581-7.

52. Park, J.Y., et al., *Polymorphism of the DNA repair gene XRCC1 and risk of primary lung cancer.* Cancer Epidemiol Biomarkers Prev, 2002. **11**(1): p. 23-7.

53. Kim, K., et al., *Interferon-gamma gene polymorphisms associated with susceptibility to systemic lupus erythematosus.* Ann Rheum Dis, 2010. **69**(6): p. 1247-50.

54. Asano, N.M., et al., *Interleukin-6 promoter polymorphisms -174 G/C in Brazilian patients with systemic lupus erythematosus.* Hum Immunol, 2013. **74**(9): p. 1153-6.

55. Zhai, Y., et al., *Association of interleukin-1 receptor-associated kinase (IRAK1) gene polymorphisms (rs3027898, rs1059702) with systemic lupus erythematosus in a Chinese Han population.* Inflamm Res, 2013. **62**(6): p. 555-60.

56. Kaufman, K.M., et al., *Fine mapping of Xq28: both MECP2 and IRAK1 contribute to risk for systemic lupus erythematosus in multiple ancestral groups.* Ann Rheum Dis, 2013. **72**(3): p. 437-44.

57. Mirowska-Guzel, D., et al., *Association of IL1A, IL1B, ILRN, IL6, IL10 and TNF-alpha polymorphisms with risk and clinical course of multiple sclerosis in a Polish population.* J Neuroimmunol, 2011. **236**(1-2): p. 87-92.

58. Wu, W., et al., *Effect of interleukin-6 polymorphism on risk of preterm birth within population strata: a meta-analysis.* BMC Genet, 2013. **14**: p. 30.

59. Tsai, H.J., et al., *Maternal cigarette smoking, metabolic gene polymorphisms, and preterm delivery: new insights on GxE interactions and pathogenic pathways.* Hum Genet, 2008. **123**(4): p. 359-69.

60. Nielsen, D.A., et al., *Genotype patterns that contribute to increased risk for or protection from developing heroin addiction.* Mol Psychiatry, 2008. **13**(4): p. 417-28.

61. Rafnar, T., et al., *Mutations in BRIP1 confer high risk of ovarian cancer.* Nat Genet, 2011. **43**(11): p. 1104-7.

62. Aktas, D., et al., *CYP1A1 gene polymorphism and risk of epithelial ovarian neoplasm.* Gynecol Oncol, 2002. **86**(2): p. 124-8.

63. Gulyaeva, L.F., et al., *Comparative analysis of SNP in estrogen-metabolizing enzymes for ovarian, endometrial, and breast cancers in Novosibirsk, Russia.* Adv Exp Med Biol, 2008. **617**: p. 359-66.

64. Bruehl, S., O.Y. Chung, and J.W. Burns, *The mu opioid receptor A118G gene polymorphism moderates effects of trait anger-out on acute pain sensitivity.* Pain, 2008. **139**(2): p. 406-15.

65. Reif, A., et al., *Meta-analysis argues for a female-specific role of MAOA-uVNTR in panic disorder in four European populations.* Am J Med Genet B Neuropsychiatr Genet, 2012. **159B**(7): p. 786-93.

66. Wu, R.M., et al., *The COMT L allele modifies the association between MAOB polymorphism and PD in Taiwanese.* Neurology, 2001. **56**(3): p. 375-82.

67. Lee, S.J., et al., *Genetic variations of alpha -methylacyl-CoA racemase are associated with sporadic prostate cancer risk in ethnically homogenous Koreans.* Biomed Res Int, 2013. **2013**: p. 394285.

68. Suzuki, K., et al., *Association of the genetic polymorphism in cytochrome P450 (CYP) 1A1 with risk of familial prostate cancer in a Japanese population: a case-control study.* Cancer Lett, 2003. **195**(2): p. 177-83.

69. Lu, X.M., L.X. Hua, and J.F. Wang, *[Association of IL-6-572C &gt; G polymorphism with the susceptibility to prostate cancer in the Chinese Han population in Jiangsu and Anhui area].* Zhonghua Nan Ke Xue, 2011. **17**(8): p. 707-11.

70. Eeles, R.A., et al., *Identification of seven new prostate cancer susceptibility loci through a genome-wide association study.* Nat Genet, 2009. **41**(10): p. 1116-21.

71. Xu, Z., et al., *Relationship between XRCC1 polymorphisms and susceptibility to prostate cancer in men from Han, Southern China.* Asian J Androl, 2007. **9**(3): p. 331-8.

72. Cho, M.H., et al., *Variants in FAM13A are associated with chronic obstructive pulmonary disease.* Nat Genet, 2010. **42**(3): p. 200-2.

73. Korytina, G.F., et al., *[Polymorphism of the genes for antioxidant defense enzymes and their association with the development of chronic obstructive pulmonary disease in the population of Bashkortostan].* Genetika, 2009. **45**(7): p. 967-76.

74. Lopez-Maderuelo, D., et al., *Interferon-gamma and interleukin-10 gene polymorphisms in pulmonary tuberculosis.* Am J Respir Crit Care Med, 2003. **167**(7): p. 970-5.

75. Wang, Y., et al., *Relationships between CYP1A1 genetic polymorphisms and bladder cancer risk: a meta-analysis.* DNA Cell Biol, 2014. **33**(3): p. 171-81.

76. Liu, C., et al., *XRCC1 Arg194Trp and Arg280His polymorphisms in bladder cancer susceptibility: a meta-analysis.* Crit Rev Eukaryot Gene Expr, 2013. **23**(4): p. 339-54.

77. Kordi Tamandani, M.K., et al., *Expression and polimorphism of IFN-gamma gene in patients with cervical cancer.* Exp Oncol, 2008. **30**(3): p. 224-9.

78. Settheetham-Ishida, W., et al., *Genetic risk of DNA repair gene polymorphisms (XRCC1 and XRCC3) for high risk human papillomavirus negative cervical cancer in Northeast Thailand.* Asian Pac J Cancer Prev, 2011. **12**(4): p. 963-6.

79. Taskiran, C., et al., *CYP1A1 gene polymorphism as a risk factor for cervical intraepithelial neoplasia and invasive cervical cancer.* Gynecol Oncol, 2006. **101**(3): p. 503-6.

80. Wang, T.J., et al., *Common genetic determinants of vitamin D insufficiency: a genome-wide association study.* Lancet, 2010. **376**(9736): p. 180-8.
