# Supplementary material for: Evolutionary triangulation: informing genetic association studies with evolutionary evidence
Source: BioData Min. 2016 Apr 2;9:12. doi: 10.1186/s13040-016-0091-7 (PMC4818851; doi:10.1186/s13040-016-0091-7)
Supplement: Additional file 10: Table S8. — SNP-based iHS score of CEU-GIH-YRI ET SNPs under 95th/5th percentile cutoff. (DOCX 15 kb) [file 13040_2016_91_MOESM10_ESM.docx]

**Table S8. SNP-based iHS score of CEU-GIH-YRI ET SNPs under 95^th^/5^th^ percentile cutoff**

| SNP | iHS in CEU | iHS in ASN^1^ | iHS in YRI |
| --- | --- | --- | --- |
| rs3794060 | -1.171 | -0.872 | -1.027 |
| rs7944926 | 2.234 | 1.732 | 1.202 |
| rs11603330 | -1.207 | -1.233 | -0.488 |
| rs12422045 | -1.207 | -1.233 | -0.533 |
| rs12785878 | -1.246 | -0.942 | -0.399 |
| rs2318792 | -0.658 | -0.258 | -2.194 |
| rs3813165 | -2.287 | -0.565 | -0.2 |
| rs4969557 | -0.658 | -0.258 | -2.196 |
| rs5925968 | -2.257 | -0.664 | 0.028 |
| rs5970824 | -1.344 | -0.444 | 0.348 |
| rs5986749 | 1.296 | -0.089 | -0.755 |
| rs1888207 | -2.093 | -0.638 | -0.721 |
| rs2297176 | -2.503 | -0.577 | -1.016 |
| rs4961736 | 3.174 | 1.386 | 1.328 |
| rs10511624 | -2.093 | -0.638 | -0.89 |
| rs10511625 | -2.052 | -0.514 | -0.47 |
| rs10733314 | -2.278 | -0.522 | -0.47 |
| rs10738445 | -2.388 | -0.627 | -0.746 |
| rs10738446 | -2.179 | -0.638 | -0.738 |
| rs10810593 | -2.354 | -0.908 | -0.738 |
| rs10962542 | -2.093 | -0.639 | -0.88 |

^1^ ASN is used as a proxy for GIH, as the online database only provides ASN.
